# Insights into the evolution, biogeography and natural history of the acorn ants, genus *Temnothorax* Mayr (hymenoptera: Formicidae)

**DOI:** 10.1186/s12862-017-1095-8

**Published:** 2017-12-13

**Authors:** Matthew Prebus

**Affiliations:** Department of Entomology & Nematology, University of California, Davis, Davis, CA 95616 USA

**Keywords:** Ants, *Leptothorax*, Phylogenomics, Rapid radiations, Social parasite, Ultraconserved elements

## Abstract

**Background:**

*Temnothorax* (Formicidae: Myrmicinae) is a diverse genus of ants found in a broad spectrum of ecosystems across the northern hemisphere. These diminutive ants have long served as models for social insect behavior, leading to discoveries about social learning and inspiring hypotheses about the process of speciation and the evolution of social parasitism. This genus is highly morphologically and behaviorally diverse, and this has caused a great deal of taxonomic confusion in recent years. Past efforts to estimate the phylogeny of this genus have been limited in taxonomic scope, leaving the broader evolutionary patterns in *Temnothorax* unclear. To establish the monophyly of *Temnothorax*, resolve the evolutionary relationships, reconstruct the historical biogeography and investigate trends in the evolution of key traits, I generated, assembled, and analyzed two molecular datasets: a traditional multi-locus Sanger sequencing dataset, and an ultra-conserved element (UCE) dataset. Using maximum likelihood, Bayesian, and summary-coalescent based approaches, I analyzed 22 data subsets consisting of 103 ingroup taxa and a maximum of 1.8 million base pairs in 2485 loci.

**Results:**

The results of this study suggest an origin of *Temnothorax* at the Eocene-Oligocene transition, concerted transitions to arboreal nesting habits in several clades during the Oligocene, coinciding with ancient global cooling, and several convergent origins of social parasitism in the Miocene and Pliocene. As with other Holarctic taxa, *Temnothorax* has a history of migration across Beringia during the Miocene.

**Conclusions:**

*Temnothorax* is corroborated as a natural group, and the notion that many of the historical subgeneric and species group concepts are artificial is reinforced. The strict form of Emery’s Rule, in which a socially parasitic species is sister to its host species, is not well supported in *Temnothorax*.

**Electronic supplementary material:**

The online version of this article (10.1186/s12862-017-1095-8) contains supplementary material, which is available to authorized users.

## Background

Ants, the most diverse eusocial insects, are among the most successful arthropod groups worldwide, inhabiting and often dominating a broad spectrum of terrestrial ecosystems [1]. The subfamily Myrmicinae is a success story within a success story: it contains nearly half of all described ant species, and six of the top ten most species-rich genera [2, 3]. *Temnothorax,* with approximately 390 named species, is one of these hyper-diverse myrmicine groups [4].

**Fig. 1:**
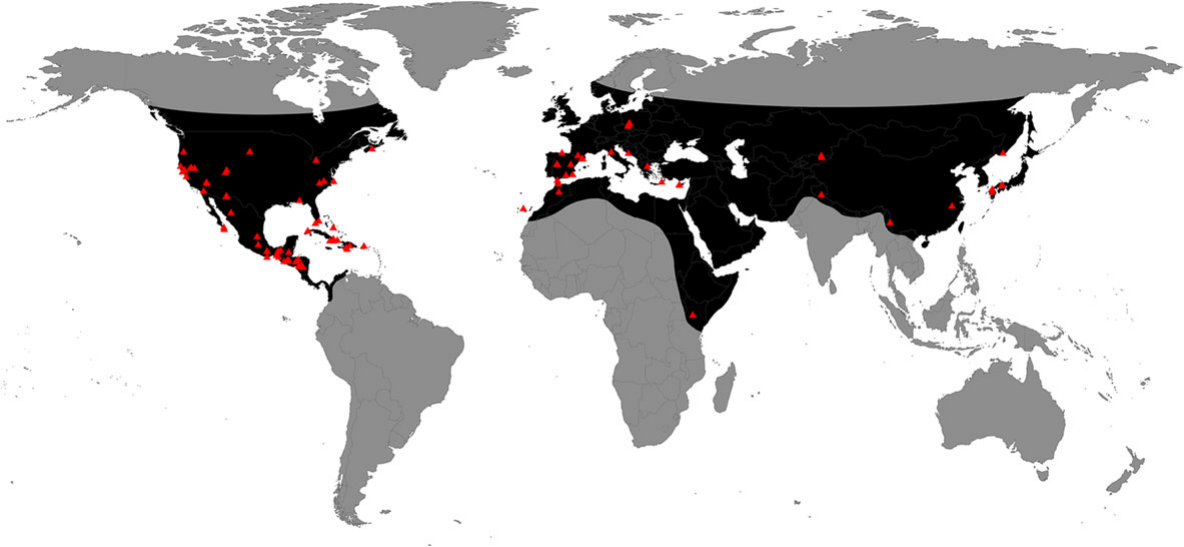
Approximate global distribution of the ant genus *Temnothorax,* indicated in black*.* Red triangles represent collection localities of specimens used in this study for gene sequencing

While the biology of most species of *Temnothorax* is poorly known, several taxa are frequently used as model organisms for social insect behavior [5, 6] and ecology [7, 8]. Studies on this genus have led investigators to discover new forms of social learning [9] and inspired hypotheses about speciation and the origins of parasitism [10]. *Temnothorax* has a primarily Holarctic distribution (see Fig. 1), but notable exceptions include several species in Sub-Saharan Africa [11] and many species in Mesoamerica, including the islands of the Caribbean [12]. Generally, *Temnothorax* ants are encountered in the mesic forests of the Northern hemisphere at mid-to-high elevations, where they are typically found under rocks, in leaf litter, or as arboreal foragers. However, these ants have adapted to a broad variety of habitats, from arid deserts to tropical rain forests, and are found at elevations from sea level up to 4000 m. The workers of most species in the Holarctic region are diminutive, timid, slow moving, and cryptically colored. Because of their inconspicuous nature, these ants are often overlooked despite their broad geographical distribution. The islands of the Greater Antilles harbor many *Temnothorax* endemics, however, that provide an exception to this habitus: the islands Cuba and Hispaniola, for example, have more than thirty described endemic species [12, 13], which are large, brightly colored, and capable of delivering powerful stings [14].

*Temnothorax* ants are believed to be trophic generalists, and have been observed scavenging for a variety of food items, including dead insects [14, 15], honeydew on leaves without tending aphids [16, 17], honeydew from tended aphids and membracids [14], the axillary nectaries of bracken ferns [18] and *Baccharis* (an Asteraceae genus) [14], seeds [14, 19], and elaiosomes [20]. In competitions at baits, they appear to be opportunistic foragers, often absconding as more aggressive species arrive, and occasionally insinuating themselves into baits dominated by other species (Prebus, pers. obs.). Recruitment of workers to food and nest sites often appears to involve tandem running [21, 22].

Nests of *Temnothorax* are generally small, often with fewer than 200 workers [23], which may be distributed among several satellite nests [24–26]. With the worker caste of most species measuring less than four millimeters in length, these ants often take advantage of small cavities for their nesting sites, such as crevices in rocks, hollow nut shells, dead twigs, or directly in the soil. *Temnothorax* colonies tend to be monogynous, with only one queen per nest. This is hardly the rule, however: this genus displays a remarkable diversity of sociometry, with many species being either functionally monogynous (with several queens present, but only one actively producing eggs), or facultatively polygynous, both within and among species [27–32].

Many species appear to be arboreal specialists, nesting in dead hollow twigs on live trees, under bark, in galls, or under the roots of epiphytes.

Several taxa have intriguing life histories: it appears that *Temnothorax* and its sister group, the *Leptothorax* genus group (LGG, composed of *Formicoxenus*, *Harpagoxenus*, and *Leptothorax*) are unusually prone to developing a set of interrelated lifestyles collectively known as ‘social parasitism’. In general, social parasites are species that depend upon a free-living social host to complete their life cycle. In social insects, this often takes the form of exploiting the host for nesting space, brood care, and trophallaxis. This feature is found broadly in the aculeate Hymenoptera [33], but is especially prevalent in the ants. However, all recorded parasitic species are known only from the subfamilies of the formicoid clade [1, 34, 35]. Several different forms of social parasitism occur within the ants [36]:

1. Xenobiosis, in which a parasitic species depends upon the host for nutrition and shelter, but cares for its own brood.
2. Temporary parasitism, in which a parasitic species depends upon the host only during its founding stage, eventually co-opting the nest site of the host.
3. Dulosis, or slave-making, in which a parasitic species produces workers, but is usually unable to forage or care for its own brood. Such species depend upon a host throughout their lives, co-opting the nest site as in temporary parasitism, but requiring periodic raids on other host nests for additional host workers, which perform the quotidian tasks within the nest.
4. Inquilinism, in which a parasitic species depends completely upon a host species for nutrition, shelter and brood care throughout its life. Typically, these species do not produce a worker caste, but divert all of their energy into producing sexuals.

All but temporary social parasitism have been recorded from *Temnothorax* and the LGG, with a recent study estimating that dulosis evolved at least six times within this clade [37].

Despite recent taxonomically focused molecular phylogenies [37–39] and extensive taxonomic works [12, 31], *Temnothorax* remains widely renowned as one of the most taxonomically difficult ant genera [40]: its status as a phylogenetically natural group has been repeatedly revised [41] and called into question [42]. This is a product of multiple factors: subtle morphological variation on a global scale among groups of species, striking departures from the morphological norm in others, morphological convergence with distantly related genera [2], weak diagnostic characters for the genus as a whole, and regional treatments of species in the absence of global understanding of the clade structure (see Additional file 1 for a more detailed account of recent molecular phylogenetic work and the taxonomic history of *Temnothorax*). The current absence of an infra-generic classification system for *Temnothorax* is not for a lack of effort, though: past taxonomists erected five subgenera to compartmentalize the species diversity of this genus, none of which are currently recognized due to a lack of clear diagnostic characters [41]. In this study, I estimate the phylogeny of *Temnothorax* on a global scale to gain insight into the structure of relationships within the genus and, furthermore, to verify that the genus is monophyletic as currently diagnosed. To maximize within-genus lineage diversity, I sampled broadly from the species groups that have been formally designated in the literature. I simultaneously examine several key aspects of the historical biogeography and evolution in the genus, which I present below.

One of the most persistent biases in the history of biogeography are the ‘out-of-the-Palearctic’ and the ‘out-of-the-Holarctic’ hypotheses, which have their origins the theories of Linnaeus and Buffon, respectively. For the ants, much progress has been done to elucidate biogeographic origins of most of the large subfamilies and several genera [2, 34, 43–53], but these biases persist for some of the common lineages in the Northern Hemisphere. For example, Bernard [54] proposed that the species of the soil-nesting Palearctic *Temnothorax rottenbergi* group were the most ‘primitive’ taxa, implying that the that the Palearctic was the center of origin for the genus. This ‘out of the Palearctic’ hypothesis has remained unchallenged, despite recent works that have recognized extremely divergent *Temnothorax* occurring elsewhere, for example the Neotropical species of the former genus *Macromischa* (referred to as the *salvini* clade, the *sallei* subclade [*allardycei + purpuratus + sallei* + *splendens* groups] and the *iris* sublclade [*iris* + *versicolor* groups] in this study). See Additional file 1 for a discussion of the *Macromischa* syndrome. To address this issue, I reconstructed the biogeographic history of the ‘core formicoxenines’ (CF, composed of *Gauromyrmex*, LGG, *Temnothorax,* and *Vombisidris*), paying special attention to dispersal between the Palearctic and the Nearctic, as well as between the islands of the Greater Antilles and the New World mainland.

There are several dispersal routes that have been proposed for movements between the Palearctic and the Nearctic. In this study, I rule out *a priori* the north Atlantic De Geer route between Northwestern Palearctic and the Northeastern Nearctic, which operated from 71 to 63 million years old (Ma) [55], as the origin of the CF is estimated at 46.7 to 59.3 Ma [2]. The Thulean route, which may have been important for early movements in the CF between the Western Palearctic and the Eastern Nearctic, opened further south than the De Geer route, and was exposed briefly 57 Ma and again 56 Ma [55]. Another route for early dispersal is between the Eastern Palearctic and Western Nearctic along an early exposure of Beringia, which first emerged for a brief period 66 Ma, again 58.5 Ma, and then was exposed intermittently from 56 Ma onward, with an extended closure from the late Miocene to the Pliocene [55, 56]. The history of land connections to the Greater Antilles, as currently understood, is much simpler: these islands have been isolated from North America since their emergence in the early Cretaceous ca. 120–125 Ma, and were briefly connected to South America along the Greater Antilles-Aves Ridge during the late Eocene to the early Oligocene, ca. 33–35 Ma [57]. This leaves overwater dispersal from mainland North or Central America as the most likely scenario. Given the morphological diversity of the Greater Antillean species, I make a first attempt to investigate the geographic origin, timing, and number of dispersals to these islands.

Another topic of particular interest is the placement of the morphologically divergent socially parasitic taxa, formerly known as the genera *Chalepoxenus*, *Myrmoxenus*, and *Protomognathus* (referred to as the *muellerianus, corsicus,* and *americanus* species groups, respectively, in this study)*.* Carlo Emery observed that insect social parasites are nearly always closely related to their hosts [58]. This generalization has come to be known as Emery’s Rule, and tends to be interpreted in two ways. In the ‘loose’ form, social parasites are congeneric with their host species, exploiting similarities between themselves and their host. Having evolved in allopatry, they are not necessarily close relatives of their hosts. In the ‘strict’ form, social parasites are the sister taxon to their hosts, and are in some cases thought to be the products of sympatric speciation [59–62]. Despite evidence that the parasitic former genera noted above are nested within *Temnothorax*, the degree to which they are related to their hosts (whether they adhere to the ‘loose’ or ‘strict’ form of Emery’s Rule), and whether they should retain status as valid genera remains a persistent topic of debate [63, 64].

The habitat under which ancestor of all ants inhabited has long been a topic of interest [65–67]. Long thought to have arisen from terrestrial habitats, the most widely accepted model of early evolution of the ants [68] suggests that they arose in the leaf litter of tropical angiosperm forests and subsequently spread to other strata. Recent work has largely supported this hypothesis [34, 47], but with the caveat that the most recent common ancestor of all crown ants was likely soil-dwelling [69]. Yet this ‘out-of-the-ground’ hypothesis has often been extended to the arboreal species in some of the most species-rich genera of ants (e.g. *Camponotus*, *Crematogaster*, *Polyrhachi*s) without much justification, as noted by Baroni Urbani [70]. For example, Bernard [54] proposed that the *Temnothorax rottenbergi* group was plesiotypic within *Temnothorax* because the species nest directly in the soil, like many of the ant species in Northern and Western Europe (and in contrast to many of the *Temnothorax* species in this region). Until the present study, the implication that *Temnothorax*, which contains many arboreal species, is ancestrally ground-nesting and later transitioned to arboreal nesting habits has not been formally examined. A recent phylogeny of the Myrmicinae suggests that two arboreal lineages, *Gauromyrmex* and *Vombisidris*, are closely related to *Temnothorax* and its sister group, the LGG [2]. In this study, I consider an alternative ‘out-of-the-trees’ hypothesis, in which *Temnothorax* arose from a terrestrially nesting ancestor, and subsequently spread to arboreal habitats.

## Methods

### Taxon sampling: Sanger dataset

An extended version of all methods can be found in Additional file 2. My taxon sampling strategy was designed to capture all the morphological, geographical, and life history variation within *Temnothorax*, with several exemplars chosen from each species group when possible. I selected 103 *Temnothorax* species, representing the recently synonymized satellite genera and 41 out of the 53 species groups currently in use, and 24 taxa of uncertain species-group affinity (see Additional file 3: Table S1 for a synopsis of the species groups). For outgroups, I used seven exemplars of the LGG, as well as representative species of the Indomalayan and Australasian genera *Gauromyrmex* and *Vomibisidris,* all of which have been shown to be close relatives of *Temnothorax* [2]. To provide additional calibration points for divergence dating analyses and for rooting the tree, I added several more taxa which encompass the phylogenetic diversity of the subfamily Myrmicinae. Voucher specimens used for DNA extractions in this study were deposited in the University of California, Davis Bohart Museum of Entomology collection; unique specimen identifiers and collection data for these can be found in Additional file 4: Table S2 and at Antweb (http://antweb.org).

### Sequence generation: Sanger dataset

DNA was extracted nondestructively or destructively from adult worker ants or pupae using a DNeasy Blood & Tissue Kit (Qiagen, Inc.) following the manufacturer’s protocols. Eight nuclear and two mitochondrial markers commonly used in ant systematics were chosen for amplification (for a complete list of primers used for amplification see Additional file 5: Table S3: 28S rDNA (*28S*), abdominal-A (*abdA*), arginine kinase (*argK*) including intron, elongation factor 1-alpha F2 (*EF1aF2*), long-wavelength rhodopsin (*LW Rh*) including intron, wingless (*wg*) including intron, rudimentary (*CAD*) including two introns, topoisomerase 1 (*Top1*), cytochrome c oxidase subunit I (*COI*) and cytochrome c oxidase subunit II (*COII*), including the spacer region between these two genes. Amplifications were performed using the PCR protocols described in Ward and Downie [71]. Sequencing was performed using PCR primers and BigDye Terminator v3.1 Cycle Sequencing chemistry, and amplicons were analyzed on an ABI 3730 Capillary Electrophoresis Genetic Analyzer (Life Technologies) at the College of Biological Sciences DNA Sequencing Facility, University of California, Davis.

### Processing and alignment: Sanger dataset

Sequence base calling and primer trimming was performed with Sequencher v 5.2.2 (Gene Codes Corporation) and exported for alignment as FASTA files. Because it has been suggested that arbitrary choice of alleles can bias phylogenetic results in some data sets [72], I retained ambiguous base calls (R or Y) for any potentially heterozygous sites. Sequences of the outgroup taxa *Myrmica striolagaster, Solenopsis xyloni, Pogonomyrmex subdentatus, Tetheamyrma subspongia, Harpagoxenus sublaevis, Formicoxenus diversipilosus* and *Vombisidris bilongrudi* were retrieved from Genbank. Identifiers for these and all new sequences generated by this study are listed in Additional file 6: Table S4.

Because intron sequence in the outgroup taxa diverged strongly from the ingroup, I maximized the number of informative sites for ingroup taxa by removing the introns from all outgroup taxa prior to alignment, retaining introns only in *Temnothorax*. Sequences were aligned with MAFFT v7.273 using the L-INS-I algorithm [73]. Alignment confidence was assessed with the program ZORRO [74]; sites that fell below an arbitrary threshold of five were trimmed from the alignment using a custom Python script developed by Fernández et al. [75]. Chi-squared test for nucleotide homogeneity were calculated with BaCoCa v 1.1.r [76]. Because the third codon position of *COI + COII* failed the chi-squared test (*P* < 0.01); it was RY-coded for downstream analyses.

### Phylogenetic inference: Sanger dataset

I used PartitionFinder 1.1.1 [77] to select data partitions and simultaneously estimate best fitting substitution models for downstream maximum likelihood (ML) and Bayesian inference (BI) analyses. ML analyses were carried out with the programs RAxML v8.2.4 [78] and IQ-TREE [79], using both best tree and bootstrap searches. BI was performed on the full dataset using MrBayes 3.2.6 [80], using the reversible jump Markov chain Monte Carlo algorithm. I ran MrBayes with two independent runs for 50 million generations, sampling every 5000 generations, and summarizing the run with a consensus tree after discarding a burnin of 25%. MCMC convergence was diagnosed by examining PRSF and average standard deviation of split frequencies between runs in the MrBayes ‘.stat’ output files. I also inspected trace plots and ESS with the program Tracer v1.6 [81] for signs of adequate parameter mixing.

To investigate the possibility of gene tree-species tree conflict, I performed single gene BI analyses with MrBayes using the same settings as for the full concatenated analysis, but reducing the number of generations and sampling frequency to 10 million and 1000, respectively. I used the program BUCKy 1.4.4 [82, 83] to conduct a Bayesian concordance analysis, using the individual gene trees as input.

### Taxon sampling: UCE dataset

Because the phylogeny inferred with Sanger sequencing data showed little support for deep relationships within the genus (see Additional file 7), I selected several exemplars of each of the well supported lineages for further analysis using ultra-conserved elements (UCEs). In total, I used 13 outgroup and 37 ingroup species in an effort to resolve the poorly supported backbone of the phylogeny.

### Sequence generation: UCE dataset

I input up to 50 ng of DNA, sheared to a target fragment size of 400–600 bp into a genomic DNA library preparation protocol following Faircloth et al. [84] as modified by Branstetter et al. [85]. I performed enrichments on pooled libraries using a set of 9898 custom designed probes (MYcroarray) targeting 2524 UCE loci in Formicidae. I followed the library enrichment procedures for the MYcroarray MYBaits kit, except that I reduced the standard MYBaits concentration to 0.1X, used custom adapter blockers instead of the standard MYcroarray blockers, and left enriched DNA bound to streptavidin beads during PCR, as described in Faircloth et al. [84]. Following post-enrichment PCR, I purified the resulting pools using magnetic speedbeads and adjusted their volume to 22 μL.

I verified enrichment success and measured size-adjusted DNA concentrations of each pool with qPCR using a SYBR® FAST qPCR kit (Kapa Biosystems) and a Bio-Rad CFX96 (Bio-Rad Laboratories), and combined all pools into an equimolar final pool. The final pool was sent to the High Throughput Genomics Facility at the University of Utah for sequencing as a single lane on an Illumina HiSeq 2500 (125 cycle paired end sequencing v4).

### Processing and alignment: UCE dataset

I demultiplexed the FASTQ data output, then trimmed away adapter contamination and low-quality bases using the program *illumiprocessor,* which is included in the PHYLUCE package. Cleaned reads were assembled de novo with PHYLUCE using a script that employs the program Trinity v 2013–02-25 [86]. I mapped the contigs from the Trinity assembly to UCE loci and extracted FASTA sequences from the matched contigs. I aligned all loci individually using the L-INS-I algorithm in MAFFT. After alignment, I used ZORRO to estimate alignment confidence values for all loci, and trimmed all sites that fell below an arbitrary threshold of five.

### Phylogenetic inference: UCE dataset

To test the sensitivity of tree topology and node support to missing data, partitioning scheme, compositional heterogeneity, evolutionary rates, gene tree-species tree conflict, and tree inference method, I ran a series of 25 analyses on 18 data subsets.

To examine the effect of missing data on phylogenetic inference, I constructed a series of matrices from loci that contained a minimum of 25%, 50%, 75%, 90%, 95%, 99% and 100% of all taxa (‘min25’, ‘min50’, ‘min75’, ‘min90’, ‘min95’, ‘min99’, ‘min100’ datasets). I then calculated summary statistics and estimated a phylogeny for each concatenated matrix with AMAS [87] and RAxML, respectively. For each RAxML tree inference, I used an unpartitioned matrix with 200 rapid bootstrap replicates, the GTR + G substitution model, and estimated the best tree annotated with bootstrap support values for each node. I wrote a custom shell script to calculate average bootstrap support for each tree. By comparing number of parsimony informative sites, proportion of parsimony informative sites, and average ML bootstrap support against percent missing data, I selected the 90% complete matrix (‘min90’) and the 75% complete matrix (‘min75’) for further analyses (see Additional file 8: Table S5 for summary statistics).

To select data partitions for the UCE data on the ‘min90’ matrix, I used the *kmeans* and *rcluster* clustering algorithms as implemented in the program PartitionFinder 2 [77, 88–90] to partition the ‘min90’ matrix into data subsets that share similarly evolving sites, limiting the model search to GTR + G. For the *kmeans* search, I used the complete concatenated dataset as input, for *rcluster* I used the full dataset partitioned by UCE locus. Additionally, I used a script developed by Borowiec [91] to extract protein coding sequence from the ‘min75’ dataset, then trimmed and aligned the extracted sequence using the local version of the program TranslatorX [92] (‘coding’ dataset). I inspected each alignment by eye using Aliview [93], and discarded sequences that were mismatched and most likely contaminants. I then partitioned the protein-coding data from each locus by codon position and used the *rcluster* algorithm in PartitionFinder 2 to gather these into similarly evolving subsets. I used the best partitioning schemes found by PartitionFinder to estimate trees with RAxML, using the same settings as in the missing data analysis above. To investigate the properties of single UCE loci in preparation for downstream analyses, I estimated single, unpartitioned UCE locus trees in RAxML using the settings listed above for the ‘min90’ dataset.

For BI analyses, I used the ‘min90’ dataset partitioned by the *kmeans* and *rcluster* algorithms, and the ‘coding’ dataset partitioned by *rcluster* as input. For each dataset, I used ExaBayes [94] to execute two independent runs, linking branch lengths across partitions and running each analysis for 1 million generations. I assessed convergence among runs and run performance with the program Tracer v1.6.0. Additionally, I inspected PSRF values, calculated average standard deviations of split frequencies, and constructed consensus trees using scripts included in the ExaBayes package.

### Sensitivity analyses: UCE dataset

To inspect the sensitivity of tree topology and node support to subsets of UCE data, I conducted an array of analyses to examine the effects of compositional heterogeneity (‘rcfv’ datasets), evolutionary rate (‘slow’ datasets), random samples of UCE loci (‘rand’ datasets), and gene-tree species-tree conflict (‘ASTRAL’ analysis). See Additional file 2 for more details.

### Constraint analyses

**Fig. 2:**
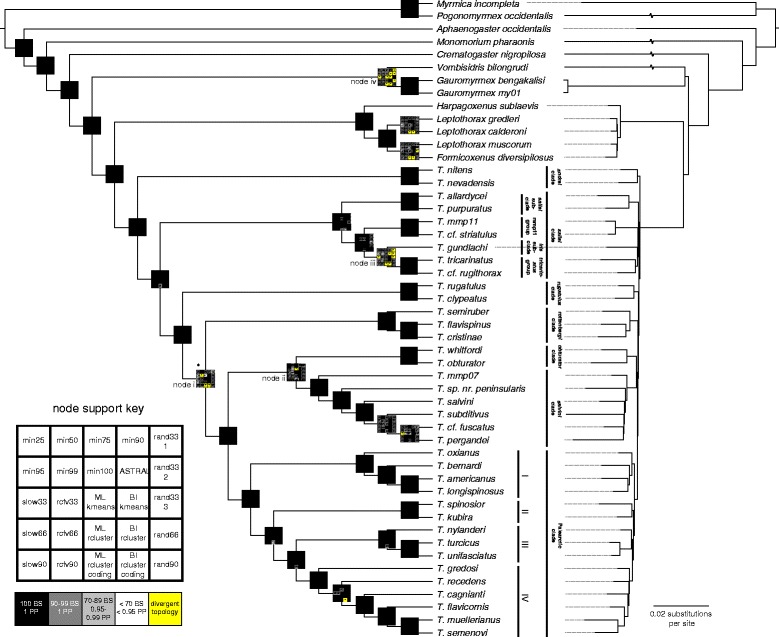
Bayesian inference (BI) phylogeny of the ant genus *Temnothorax*, including myrmicine outgroups, estimated with the ‘rcluster’ partitioned analysis of a concatenated 2098-locus ultra-conserved element (UCE) dataset. Cladogram with support values from 25 distinct analyses represented at each node (left) contrasted with a phylogram from the same analysis (right). Yellow highlighted cells indicate that the corresponding analysis diverged topologically from the result depicted in the cladogram. Nodes that were subject to topology experiments are indicated with lowercase roman numerals

Four ‘backbone’ nodes were poorly supported among the UCE analyses (labeled i-iv in Fig. 2) and each was subjected to constraint analyses. Because Bayesian analyses are computationally expensive on large datasets, I performed the stepping stone sampling method developed by Xie et al. [95] on the full Sanger sequencing dataset, constraining the trees into two sets of competing hypotheses to calculate Bayes factors, in accordance with the recommendations of Bergsten et al. [96] (see Additional file 2 and Additional file 3: Table S1 for the hypotheses tested).

I ran these analyses in MrBayes 3.2.6 with two independent runs per analysis, setting the value of the alpha-shape parameter of the beta distribution to 0.4, number of chains to four, and sampling 25.5 million MCMC generations every 100th generation for 50 steps between the posterior and the prior, and discarding the initial 500 thousand MCMC steps as burn-in. After initial runs, the marginal likelihoods of the topology tests within *Temnothorax* were all very close; I subsequently increased the number of independent runs for each test at nodes i-iii to four.

**Fig. 3:**
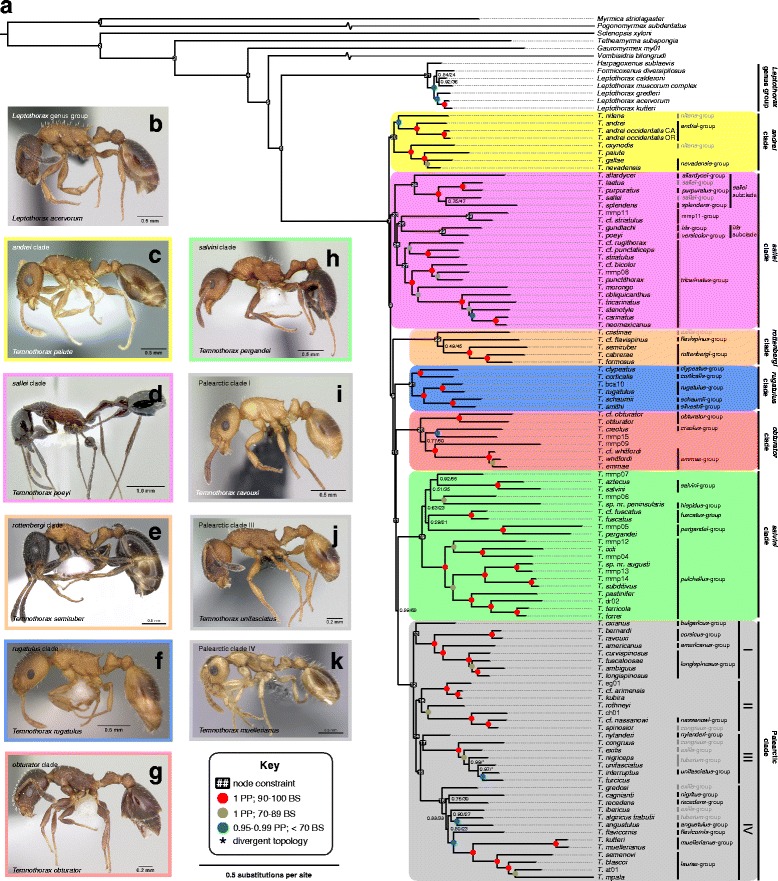
**a** Bayesian inference (BI) phylogeny of the ant genus *Temnothorax*, including myrmicine outgroups, estimated with a constrained, partitioned analysis of a concatenated 10-gene dataset. Node support values are given in Bayesian posterior probability (PP) and maximum likelihood bootstraps (BS); the latter were generated in a separate maximum likelihood (ML) analysis. A node support key is provided in the figure. For all nodes that do not conform to the three categories given, actual support values are noted as PP/BS. The topology of two nodes in the Palearctic III clade were divergent between BI and ML analyses; these nodes are indicated by asterisks. Node constraints used in the BI and ML analyses are indicated by numbers in black boxes. Clade and species group designations suggested by this study or which were supported are shown in black to the right of the phylogram, while species groups from the literature that were found to be paraphyletic are in grey. (b-k) Images of exemplar species of the clades and subclades designated in this study, from antweb.org (**b**) *Leptothorax acervorum* Fabricius 1793, worker, profile view, CASENT0173138, photo: April Nobile. **c** *Temnothorax paiute* Snelling et al. 2014, worker, profile view, CASENT0005932, photo: Marek Borowiec. **d** *T. poeyi* Wheeler 1913, worker, profile view, CASENT0106241, photo: Michael Branstetter. **e** *T. semiruber* André 1881, worker, profile view, CASENT0281557, photo: Estella Ortega. **f** *T. rugatulus* Emery 1895, worker, profile view, CASENT0102843, photo: Jen Fogarty. **g** *T. obturator* Wheeler 1903, worker, profile view, CASENT0104756, photo: April Nobile. **h** *T. pergandei* Emery 1895, worker, profile view, CASENT0172989, photo: April Nobile. **i** *T. ravouxi* André 1896, worker, profile view, CASENT0173641, photo: April Nobile. **j** *T. unifasciatus* Latrielle 1798, worker, profile view, CASENT0173188, photo: April Nobile. **k** *T. muellerianus* Finzi 1922, worker, profile view, CASENT0270722, photo: Shannon Hartman

I reconciled the results of these two sets of analyses by constraining the backbone of the more species-rich Sanger sequencing dataset based on the results of the UCE analyses and stepping stone analyses, and re-estimated the phylogeny in BI and ML frameworks (see Fig. 3 for the final constraints used). For BI, I used MrBayes with the settings for the full, unconstrained dataset analysis. For the ML analyses I used and a beta version of IQ-TREE and RAxML, with the settings used above in the unconstrained analyses, and using the ‘–g’ option for both programs, which uses a multifurcating tree as input for the constrained tree search.

### Morphology

To properly place the fossil taxon *Temnothorax praecreolus* in the phylogeny in preparation for downstream divergence dating analyses, I recorded characters and took measurements from this fossil, in addition to all *Temnothorax* worker specimens used in the phylogeny. The final dataset included 29 characters, which were either inherently discrete, discretized continuous characters, or discretized indices (see Additional file 9 and Additional file 10: Table S6).

### Divergence time analysis

Divergence dates in *Temnothorax* were inferred using a combination of node- and tip-dating approaches in the program BEAST 2.3.0 [97]. I used morphological data to place *Temnothorax praecreolus*, but due to the uncertainty of the placement of Baltic amber specimens (see Additional file 1), I compared two analyses, using these fossils to calibrate: (a) the node subtending the CF (b) the node subtending [LGG + *Temnothorax*] (see Additional file 2 for details on the calibrations used). I used the combined concatenated Sanger molecular and morphology dataset as input, partitioning the molecular data with the scheme suggested by ParititionFinder. I constrained the tree topologically based on the results of the UCE and stepping stone analyses (see Fig. 3 for constraints). Each analysis consisted of two runs of 100 million generations, sampling every 10 thousand generations. Time series plots and ESS values were analyzed with Tracer to assess burn-in and to determine whether runs had converged and chains were mixing adequately. I summarized the tree files with LogCombiner v1.8.0 and TreeAnnotator v1.8.0 after discarding the initial 25% of trees as burn-in.

### Biogeographic analysis

**Fig. 4:**
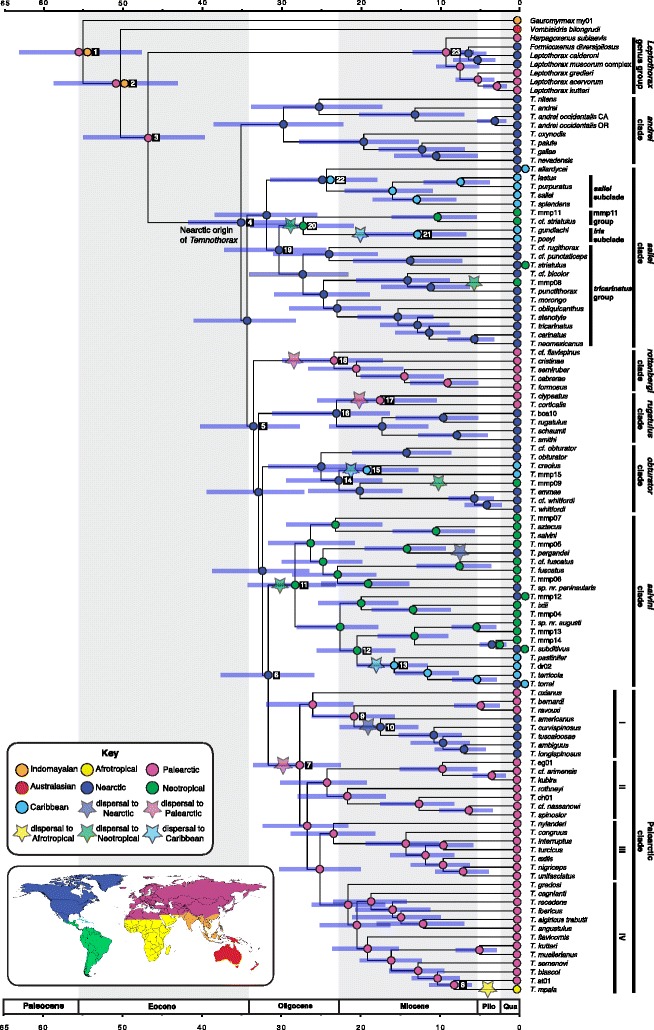
Chronogram of the core formicoxenines, sensu Ward et al. [2], from a BEAST analysis, with *Temnothorax praecreolus* and the myrmicine outgroups not shown. Bars depict the 95% HPD (highest posterior density). Biogeographical history as inferred from an average of six models tested in BioGeoBEARS is superimposed on the chronogram. Nodes with two areas indicate taxon occupancy of both realms

I used the consensus chronogram from the BEAST analysis as input for the likelihood-based R package BioGeoBEARS [98], pruning all outgroup taxa from the tree except for the CF. I used a biogeographical classification scheme following previous studies (e.g. [2]), but discretized the Caribbean islands, including the Bahamas, into a separate biogeographical unit given the high diversity of *Temnothorax* species on these islands, and their historical isolation from the mainland [99] (see the key in Fig. 4 for a summary of the areas used). Because no extant species in the phylogeny inhabits more than two biogeographical areas, I limited the maximum ancestral species range to 2 units. Because the ancestor of the CF is inferred to have arisen between 60 and 50 Ma, relatively little tectonic drift with respect to the current positions of the continents has taken place in the evolutionary history of this group; therefore, I used a single time slice for this analysis. I imposed matrices of dispersal constraints, dispersal multipliers, area adjacencies, and allowed ancestral ranges on the analysis (see script and input files on Dryad). I ran the program over the six models included in the example script, and compared these via AICc score. Because the models offered alternate reconstructions, I averaged the results across all six models using the AICc weights in the output file. Finally, I examined the sensitivity of the ingroup reconstruction to incomplete outgroup sampling (see Additional file 2 for details).

### Ancestral state reconstruction

To reconstruct the historical shifts in nesting habitat and parasitism in *Temnothorax*, as well as the potential effects of these characters on diversification rates, I estimated the ancestral states of these two traits using the hidden state speciation and extinction (HiSSE) model [100]. I gathered nesting habitat and behavioral data from the literature, collection information, and personal observation (see Additional file 11: Table S7), and coded all extant taxa according to whether they are:

(0) arboreal, nesting exclusively in vegetable matter, living or dead, above the soil and leaf litter layer, or
(1) terrestrial, nesting in variety of habitats, but always including the soil, leaf litter layer, and/or fallen, rotten logs.

In the parasitism analysis, species were coded as:

(0) free living, if nests are founded independently, or
(1) social parasites, if new nests are founded exclusively in the nests of other ants; I include the xenobiotic genus *Formicoxenus* in this category.

Because the taxon sampling in this phylogeny is incomplete, I used global sampling fractions for each analysis, modeled as the proportion of species in each state that are included in the phylogeny.

For the nesting habit dataset, I searched among 59 models, composed of BiSSE-like models, variations on the HiSSE model, and null models in which diversification rates are independent of character states (see Additional file 2 and Additional file 12: Table S8 for details on model specification). I included transition-restricted models that disallowed transitions from arboreal to terrestrial nesting and, based on preliminary results, models that disallowed a hidden state within arboreal nesting. HiSSE initially returned a surprising result, showing that a hidden state associated with arboreal nesting was responsible for a two-fold decrease in net diversification rate. I inspected reconstructions of the states on the tree, and found a single transition to the hidden state at the root of the tree, subtending the long-branched outgroups. After trimming *Gauromyrmex* and *Vombisidris* from the dataset, all traces of character dependent diversification rates disappeared.

For the parasitism dataset, I constructed 41 models using most of the models mentioned above, with a subset disallowing transitions from parasitism to free living, but unlike the nesting habit model search, I didn’t include HiSSE models that disallowed hidden states. For both datasets I summarized all reconstructions, accounting for model uncertainty, by averaging over all models after weighting each model by its AIC score.

## Results

### Sanger dataset characteristics

The resulting concatenated data matrix was 8010 bp in length with 4.1% missing data, containing 3469 (43.3%) variable sites and 2608 (32.5%) parsimony-informative sites, with most of the variability (52%) concentrated in the third codon positions of the alignment (see Additional file 13: Table S9). The data matrix is nearly complete, with *COI* + *COII* missing for the outgroups and six ingroup taxa (*Temnothorax* dr02*, T. mpala, T. rothneyi, T. smithi, T.* cf. *striatulus*, and *T. unifasciatus*), the first exon of *Wg* missing for several outgroup taxa (*Formicoxenus diversipilosus, Harpagoxenus sublaevis, Vombisidris bilongrudi*, and *Tetheamyrma subspongia*) and ingroup taxa (*Temnothorax* mmp15, *T. mpala, T. obturator, T. rothneyi, T. semiruber,* and *T. smithi*). In *Temnothorax stenotyle*, the intron of *ArgK* has apparently been lost: both exonic fragments amplified normally, but the intervening intron was missing upon assembly and alignment. I confirmed this by inspecting an argarose gel of the amplicon that usually contains intron; the fragment was shorter by approximately 100 bp in comparison to other taxa.

Among the nuclear protein coding genes, the exons exhibit between 22 and 48% parsimony informative sites (PIS), with *COI + COII* having the greatest proportion, and *abdA* having the smallest. The exonic data contributed the most PIS to the Sanger dataset, containing 2070 sites out of 2608 in the entire matrix, or 79% of all informative sites. Introns contained a high proportion of informative sites as well, with 29–56% PIS, contributing 424 informative sites the overall dataset (16.5% of the total PIS). 28S was the most conserved DNA fragment used in the dataset, contributing only 114 informative sites, or 4.5% of all PIS in the dataset.

Inspection of base frequencies among the data subsets revealed that the third codon position of *COI + COII*, as well as the introns of all fragments except *LWRh* are highly AT biased, with the proportions ranging between 72 and 80%. Conversely, *AbdA* and the third codon positions of all nuclear genes, save *CAD*, were GC rich, with proportions ranging between 60 and 77%. Aside from the third codon position of *COI + COII*, no subset of the data displayed significant compositional heterogeneity among taxa.

### Sanger dataset phylogeny

The best scheme found by PartitionFinder consisted of 14 partitions (see Additional file 14: Table S10). The best tree with branch lengths from the BI analysis of the unconstrained, concatenated and partitioned data is shown in Additional file 7, Fig. H, including support values from both Bayesian and maximum likelihood analyses. The trees from the BI and ML analyses are nearly identical in topology, differing in inferred relationships and branch lengths only at nodes that were weakly supported in both. Both methods recovered *Temnothorax* as a monophyletic group with high support (PP = 1, BS = 100). In concordance with Ward et al. [1], the Indomalayan *Gauromyrmex* my01 was recovered as sister to all other CF in the study, and the LGG was monophyletic across analyses. In contrast to the relationship found in Ward et al. [2], the Australasian species *Vombisidris bilongrudi* was inferred to be sister to *Temnothorax*, but this relationship received only moderate support (PP =1, BS = 53–71). Furthermore, ten well-supported clades were identified within *Temnothorax*, although the relationships among these clades were poorly supported in all analyses based on Sanger data. All ten clades had maximum support (PP =1 and BS =100), except for clade 2 (PP 1, BS 88) and clade 5 (PP 1, BS 99). Within the ‘Palearctic’ clade, three well-supported subclades emerged (PP 1, BS 100), in addition to a fourth subclade with mediocre support (PP 1, BS 63).

In general, where trees resulting from data exclusion experiments differed topologically from analyses using the full dataset, it was almost exclusively at nodes that were weakly supported across analyses. Many of the relationships within the *salvini* clade continued to have low support among data subset analyses, with several highly-supported species pairs and one well-supported subclade. The position of the mmp11 group was extremely unstable among analyses, being placed variously as sister to the *rottenbergi* clade, as sister to [*sallei* subclade + *iris* subclade + *tricarinatus* group], or as sister to all *Temnothorax.* Additionally, the topology within the Palearctic clade was very plastic among analyses, but was consistently composed of the above mentioned four subclades, in addition to the rogue taxa *T. nylanderi* and *T. oxianus*.

The primary concordance tree with branch lengths in units of concordance factors is given in Additional file 7. Concordance factors are interpreted as the estimated proportion of the data that supports a given node, for example, the node subtending *Temnothorax* is supported by a high concordance factor (CF = 0.88). While all major subclades were recovered (except for the mmp11 group and the *gunlachi* group), only five of the major clades had concordance factors over 0.4. The four subclades of clade 10 were recovered as well. Again, the relationships among all the 10 major clades of *Temnothorax* were poorly supported, with the concordance factors of the “backbone” of the phylogeny ranging between 0 and 0.18. After considering single gene consensus trees (see Additional file 15), this is most probably due to a paucity of informative sites in the dataset that resolve the backbone of the phylogeny, rather than gene-tree conflict.

### UCE dataset characteristics

After assembling the data and matching probes to contigs, the mean number of UCE loci per specimen was 2314, with a mean length of 1002 bp and a mean coverage score of 60 X (see Additional file 16: Table S11). After filtering the loci by weighing matrix completeness against proportion of parsimony informative sites and mean bootstrap support, I selected the ‘min90’ dataset for further analysis. The ‘min90’ dataset included 2098 UCE loci with a mean length of 744 bp, which resulted in an aligned, concatenated matrix 1,561,581 bp in length, of which 404,463 sites (26%) were parsimony informative. All sequences have been submitted to the NCBI Sequence Reads Archive (BioProject PRJNA393044).

The dataset was slightly AT rich, with these bases comprising 56% of the dataset. In a chi-squared test, no loci were found to be diverging significantly from base composition homogeneity across taxa.

### UCE dataset phylogeny

A cladogram with the topology from the ExaBayes analysis with the ‘rcluster’ partitioning scheme is contrasted with the phylogram from the same analysis and presented in Fig. 2; see Additional file 17 for a synopsis of all trees resulting from filtering treatments. The ML and BI approaches using the ‘rcluster’ partitioning scheme recovered identical topologies, differing only in their support of certain nodes (PP = 1, BS ≥ 90). Across the sensitivity analyses, however, topology diverged at several nodes. The phylogenetic position of *Vombisidris bilongrudi* was particularly volatile: it was found to be sister to the clade consisting of the LGG + *Temnothorax* in the ‘slow33’, ‘ML kmeans’, ‘BI kmeans’, and ASTRAL analyses, while it was found to be sister to *Gauromyrmex* + the LGG + *Temnothorax* in the coding data only, and two of the ‘rand33’ and the ‘rand66’ datasets each.

Another node that was unstable across a large subset of analyses was the node subtending the *iris* subclade and *tricarinatus* group; in this case, the trees resulting from the ASTRAL, two of the rand33 datasets, and the coding data only datasets recovered the *iris* subclade as sister to the mmp11 group with low-to-moderate support (BS = 47–85). The ‘slow33’ and one of the ‘rand33’ loci datasets recovered the *iris* subclade as sister to [*tricarinatus* group + mmp11 group] with high support (BS = 96–100).

The positions of the *rugatulus* and *rottenbergi* clades were unstable among analyses as well, with the *rugatulus* clade being recovered as sister to [*salvini* group + *obturator* group + Palearctic clade] in the ‘min95’ and coding data only datasets. In the ‘min99’ dataset, the *obturator* clade was recovered as sister to the Palearctic clade. Within the *salvini* clade, the ‘slow33’ dataset recovered *T. pergandei* as being sister to *T.* cf. *fuscatus* + *T. subditivus*. In the BI coding data only analysis, *T. recedens* formed a clade with *T. gredosi*. It is worth noting that in the case of *Vombisidris* and *T. gundlachi,* the datasets composed of a reduced set of random loci often arrived at the same topology as the other reduced datasets, suggesting that in these cases, the topology may rest on a small fulcrum of data.

The subclades of *Temnothorax* that were identified in the analysis of the Sanger sequencing dataset are recovered with maximum support across all analyses, except for the *iris* subclade, which is only represented by *T. gundlachi* in the UCE dataset. Other than the nodes highlighted above, the relationships among the ten subclades that were identified in the Sanger analysis are fully resolved, receiving maximum support across all analyses. Additionally, the rogue taxa *T. oxianus* and *T. nylanderi* are recovered as members of the subclades ‘I’ and ‘III’ of the Palearctic clade, respectively.

### Constraint analyses

The results of the constraint analyses are given in Table 1. I interpret the results of the Bayes factor tests following the suggestions of Kass & Raftery [101], assigning levels of support to the following ranges of 2lnBF: 0–2, not worth explaining; 2–6, positive support; 6–10, strong support; and >10, very strong support.

- node i. *rugatulus* clade sister to [*obturator* clade + Palearctic clade + *salvini* clade] received strong support 2lnBF = 6.96).
- node ii. *obturator* clade being sister to the Palearctic clade vs. the *salvini* clade was equivocal (2lnBF = 0.62).
- node iii. *Iris* subclade sister to the mmp11 group received strong support (2lnBF = 6.94).
- node iv. *Vombisidris* as sister to the LGG + *Temnothorax* was positively supported (2lnBF = 5.76).

**Table 1:** Results of the stepping-stone topology tests on the Sanger sequencing dataset at nodes indicated in Fig. 2

node	hypothesis	run 1	run 2	run 3	run 4	mean	2lnBF
i	[rottenbergi clade[salvini clade, obturator clade, Palearctic clade]] (H0)	−79,370.44	−79,369.39	−79,371.1	−79,376.09	−79,370.35	−6.96
	[rugatulus clade[salvini clade, obturator clade, Palearctic clade]] (H1)	−79,368.98	−79,365.99	−79,366.52	−79,369.67	−79,366.87	
ii	[obturator clade, salvini clade] (H0)	−79,366.2	−79,367.15	−79,371.6	−79,368.39	−79,367.18	−0.62
	[obturator clade, Palearctic clade] (H1)	−79,368.98	−79,365.99	−79,366.52	−79,369.67	−79,366.87	
iii	[iris subclade, tricarinatus group] (H0)	−79,368.98	−79,365.99	−79,366.52	−79,369.67	−79,366.87	−6.94
	[iris subclade, mmp11 group] (H1)	−79,368.52	−79,362.01	−79,371.47	−79,369.57	−79,363.4	
	[iris subclade, tricarinatus group] (H0)	−79,368.98	−79,365.99	−79,366.52	−79,369.67	−79,366.87	2.26
	[tricarinatus group, mmp11 group] (H1)	−79,373.33	−79,366.63	−79,371.94	−79,371.57	−79,368	
	[iris subclade, mmp11 group] (H0)	−79,368.52	−79,362.01	−79,371.47	−79,369.57	−79,363.4	9.2
	[tricarinatus group, mmp11 group] (H1)	−79,373.33	−79,366.63	−79,371.94	−79,371.57	−79,368	
iv	[Gauromyrmex, Vombisidris] (H0)	−79,370.23	−79,372.61	–	–	−79,370.83	−5.76
	[Gauromyrmex[Vombisidris, LGG, Temnothorax]] (H1)	−79,367.86	−79,368.04	–	–	−79,367.95	
	[Gauromyrmex, Vombisidris] (H0)	−79,370.23	−79,372.61	–	–	−79,370.83	7.48
	[Vombisidris[Gauromyrmex, LGG, Temnothorax]] (H1)	−79,375.57	−79,374.08	–	–	−79,374.57	
	[Gauromyrmex[Vombisidris, LGG, Temnothorax]] (H0)	−79,367.86	−79,368.04	–	–	−79,367.95	13.24
	[Vombisidris[Gauromyrmex, LGG, Temnothorax]] (H1)	−79,375.57	−79,374.08	–	–	−79,374.57	

### Divergence dates

The chronogram from the BEAST analysis of the constrained Sanger sequencing and morphology dataset is presented in Fig. 4. *Temnothorax praecreolus,* (not shown) was recovered as sister to a clade of mainland Meso-American species of the *salvini* clade in the BEAST analysis, whereas in the MrBayes analysis it was sister to a clade of mostly Caribbean *salvini* clade species, the *pulchellus* species-group (see Additional files 18 and 19). The BEAST chronogram differs topologically from the MrBayes constrained phylogram (see Fig. 3) at several nodes with low support: in the *rottenbergi* clade, *T. cristinae* and *T.* cf. *flavispinus* switch positions; the positions the non-*pulchellus* group members of the *salvini* clade continue to be unstable, as in the constrained BI and ML analyses; the positions of all species within the Palearctic III subclade except *T. nylanderi* and *T. congruus* are unstable, as well as all non-*flavicornis*, *muellerianus* and *laurae* group species in Palearctic IV subclade.

The crown group of *Temnothorax* is estimated to have arisen 35 Ma (95% highest posterior density (HPD) = 28–41 Ma), with the stem group + LGG emerging roughly 11 Ma earlier: 46 Ma (95% HPD = 39–54 Ma). Immediately following the origin of the crown *Temnothorax* at the Eocene-Oligocene transition, there appears to have been a rapid succession of splitting events. Interestingly, the LGG, and *Temnothorax* to a lesser extent, are both subtended by long branches (38 Ma in the LGG*,* 11 Ma in *Temnothorax*). Similarly, long branches subtending large clades (> 5 Ma) appear in the *rottenberg*i-, *rugatulus-* and *obturator* clades, suggesting extensive pruning if speciation is assumed to be constant over time.

### Biogeography

The BioGeoBEARS analysis of the empirical data identified the dispersal-extinction-cladogenesis + jump dispersal (DEC + J) model as the best fit in comparison to its null model, DEC, and the four other models tested (see Additional file 20: Table S12). The jump (+J) models always performed significantly better than the alternatives, and the results of the models with the second and third highest likelihoods, DIVALIKE + J and BAYAREALIKE + J, largely agree with the DEC + J model, differing mainly in the size of ancestral areas at several nodes (see [Additional file 21: Table S13). The model-averaged results of the empirical data agreed with the results of the sensitivity analysis almost exactly, except for the LGG, which was reconstructed as having a Holarctic MRCA in the sensitivity + empirical average (see Additional file 21: Table S13 and Additional file 22).

The results suggest an ancestral Palearctic-Indomalayan distribution for the origin of the CF (node 1), with *Gauromyrmex* and *Vombisidris* remaining in the Indomalayan realm, with the latter genus presently extending into the Australasian region (node 2). There is some uncertainty surrounding this reconstruction though, with alternative empirical models and sensitivity analyses suggesting a strictly Indomalayan origin, or an Indomalayan Australasian origin (see Additional file 21: Table S13). Among nearly all models and sensitivity analyses, the ancestral range of the LGG + *Temnothorax* is estimated to be Palearctic (node 3). There is quite a bit of uncertainty about the ancestral range of the LGG, with most of the sensitivity analyses suggesting a Holarctic origin, while the empirical data suggests a Palearctic range (node 23). The MRCA of *Temnothorax*, on the other hand, was reconstructed as Nearctic across nearly all the analyses (node 4).

The ancestor of all crown *Temnothorax* dispersed to the Nearctic sometime in the middle to late Eocene, 35–47 Ma (nodes 3, 4). Much of the early diversification of *Temnothorax* is inferred to be in the Nearctic realm, with a striking pattern of dispersal: soon after a rapid series of splitting events, there was a period of large-scale dispersal, spanning the Oligocene to middle Miocene. During this period, at least four separate lineages expanded their range or dispersed into the Greater Antilles. The ancestor of the *sallei* subclade is inferred to have had a Nearctic-Caribbean distribution (node 22), retained in the extant species *T. allardycei*, which has variously been categorized as an exotic species from the Neotropics [102, 103], or a species endemic to the Bahamas and Florida [104–106]. A second dispersal event in the *sallei* clade occurred in the same time frame, represented by the terricolous species *T. gundlachi* and *T. poeyi* (node 21). The ancestor of two members of the *obturator* clade, *T. creolus* and *T.* mmp15, the former found on the island of Hispaniola and the latter an endemic of Puerto Rico, also dispersed to the Caribbean from the Nearctic in the early Miocene (nodes 14, 15). Several members of the *pulchellus* group in the *salvini* clade also dispersed to the Caribbean islands from the mainland Neotropics in the early Miocene (nodes 12, 13), with *T. torrei* extending its range to the Nearctic sub-tropics between the Pliocene and the present.

Another series of Oligocene to middle Miocene dispersals to the mainland Neotropics from the Nearctic occurred in several disparate lineages: the *salvini* clade has an extensive history of diversification in this realm, dispersing from the Nearctic in the early Oligocene (nodes 6, 11) with the ancestors of *T. pergandei, T. peninsularis*, and *T. mmp12* dispersing back to the Nearctic sometime between the middle Miocene and the present. In the *sallei* clade, the ancestor of the mmp11 group and the *iris* subclade dispersed to the Neotropics at some point during the early Oligocene, 27–31 Ma (nodes 19, 20), with the *iris* subclade dispersing to the Caribbean 13–27 Ma (nodes 20, 21); mmp08 dispersed much later, at most 12 Ma, while *T. striatulus* extended its range into the Neotropics during this period. A single lineage from the *obturator* clade is known from cloud forest in the Neotropical region, dispersing from the Nearctic at some point after the early Miocene.

Perhaps the most significant dispersal events during the Oligocene to middle Miocene involve movements between the Nearctic and Palearctic. For example, the ancestor of the extremely diverse Palearctic clade dispersed to Eurasia from the Nearctic between 27 and 31 Ma (nodes 6, 7), the ancestor of *T. clypeatus* and *T. corticalis* dispersed between 16 and 23 Ma nodes 16, 17), and the ancestor of the *rottenbergi* clade dispersed between 23 and 33 Ma (nodes 5, 18). Because the dates for these dispersal events were well after the Eocene, I rule out the early Eocene trans-Atlantic Thulean route [55]; presumably these occurred over the Beringian land bridge which was present intermittently throughout the Paleocene to the late Miocene [56]. A hypothesis of east to west movement to the Palearctic out of the Nearctic is supported with a pectinate series of lineages in the Palearctic clade: the Palearctic II subclade, composed of mostly east Asian taxa, is sister to the mostly central and western Eurasian Palearctic III clade and the mostly Mediterranean Palearctic IV subclade. The Palearctic I subclade, however, is a bit enigmatic in that *T. oxianus*, collected from Kyrgyzstan, is the sister taxon to a clade of western European and eastern North American taxa, suggesting a back-dispersal to the Nearctic (nodes 9, 10). Within the Paleartic IV subclade, dispersal to the Afrotropics from the Palearctic occurred after the late Miocene (node 8), with the split between *T. mpala* and *T.* at01 closely coinciding with the most recent estimates of Saharan aridification, which occurred 7–11 Ma [107].

### Ancestral state estimation

After trimming the long-branched outgroups from the nesting habit dataset, there were two models that fit equally well based on AIC score. Both were relatively simple HiSSE models with three free parameters, in which each had one turnover rate, one extinction fraction for all states, transition rates equal, and no hidden state within arboreal nesting. They differed in that one model allowed transitions from arboreal to terrestrial nesting, and one didn’t. No character dependent diversification was detected for arboreal nesting. The model-averaged ancestral state reconstruction suggests that the MRCA of *Temnothorax* was ground-nesting, with multiple independent transitions to arboreal nesting in the Oligocene (see Fig. 4). Transitions from arboreal to terrestrial nesting were recovered in only one case, in the *rugatulus* clade.

The best model for the parasitism dataset was a complex BiSSE-like model with all parameters free. However, several competing models fell within a ΔAIC of 2 or less, and among these was a character independent diversification model (CID-4, a four-rate model with transition rates equal). Indeed, when the models are averaged, there is little support for character dependent diversification when parasitism is considered (see Additional file 23). The model-averaged ancestral state reconstruction suggested multiple independent transitions to parasitism, all of which were in the middle Miocene or later.

## Discussion

### Phylogeny

The phylogenetic analyses from two sequencing approaches and on multiple data subsets recover identical clades in *Temnothorax* and largely agree on the relationships among them. Although even the large UCE dataset could not resolve some of the relationships in the backbone satisfactorily, constraint analyses on the Sanger dataset resolved most of the difficult nodes. *Temnothorax* was recovered as a natural group with high support, as was the sister group relationship the *Leptothorax* genus group. The species groups, former subgenera, and satellite genera of the core formicoxenines, including *Temnothorax* have a long and controversial history. A phylogenetic framework for understanding the relationships within this intriguing group of ants has been sorely lacking, which has hampered taxonomic revisionary efforts and our understanding of the pace and mode of evolution within this genus. In this study, the inclusion of the former genera *Chalepoxenus*, *Myrmoxenus*, and *Protomognathus* in *Temnothorax* is found to be justified. The former genus *Macromischa* is revealed to be polyphyletic, confirming earlier suppositions [12, 41], and composed of members of the *salvini*, *obturator*, and *sallei* clades, interdigitated with the former subgenus *Myrafant*. A striking pattern within *Temnothorax* is strong morphological conservatism within and between groups (e.g., compare taxa from the *tricarinatus* group and the *andrei, rugatulus*, and Palearctic clades) punctuated by extreme morphological departures (e.g. the Caribbean members of the *sallei* clade, and the *salvini* clade).

### Biogeography

The Cenozoic has been a period of dramatic fluctuations in climate that have substantially affected faunal and floral distributions [108]. The diversification of the CF encompasses much of this era, so any discussion of evolution needs to be viewed in the context of responses to changes in climate regime. Furthermore, the historical biogeographic patterns of Holarctic flora and fauna are some of the most thoroughly studied, and are generally understood as the products of a continuous habitat across the Northern Hemisphere, connected intermittently by the De Geer and Thulean routes across the North Atlantic during the late Cretaceous and early Tertiary [109, 110], or nearly permanently by the Beringian route from the middle Cretaceous through the Tertiary, and then intermittently connected during the Quaternary [110–112]. In the discussion below, I present the historical biogeography and trait evolution of *Temnothorax*, considering a timeline of major geologic events during the evolution of the genus.

The origins of the CF are obscured by uncertainty in the divergence dates, but the biogeographic analysis suggest that the MRCA of this clade arose 47–64 Ma with a Palearctic-Indomalayan range. This period includes late Paleocene Thermal Maximum (LPTM) [108], an event that was marked by a steep 5–6 °C rise in deep-sea temperature over a very short time period, < 10 ka (thousand years) [113–115], and the wide-scale dispersal and radiation of land plants and mammals [116–120], likely facilitated by expansion of broadleaf evergreen forests into high latitudes [121–123]. By 52 Ma, at the Eocene Climatic Optimum, the ancestor of the LGG + *Temnothorax* had a more restricted Palearctic distribution. The ancestor of *Temnothorax* dispersed to the Nearctic during the middle to late Eocene, but there is little evidence to suggest whether this was by either the Thulean or Beringian route.

The results of this study support an origin of the crown-group *Temnothorax* in the Nearctic realm roughly 35 Ma, at the Eocene-Oligocene transition, running contrary to Bernard’s ‘out-of-the-Palearctic’ hypothesis. Early rapid diversification in the Nearctic was followed by large-scale dispersal. By this time, the North Atlantic Thulean route had closed, leaving Beringia as the most probable dispersal route (although Tiffney [110] suggests that a North Atlantic route may have operated as a series of stepping stone islands into the middle Tertiary). The Eocene-Oligocene transition (34 Ma) is characterized by another drastic swing in global temperature, this time with the deep-sea cooling rapidly by 5 °C, initiating the formation of an Antarctic icecap and a glacial period that lasted roughly 400 ka. Like the LPTM, this event had dramatic effects on the global biota, leading to abrupt turnover [124, 125], the origination of many plant and animal lineages [126, 127], retraction of evergreen broadleaf forests toward the equator, expansion of seasonal deciduous forest, and widespread aridification [121, 122]. Given the contemporary habitat affinities of many species in the genus, the common ancestor of *Temnothorax* may have already adapted to living in cool and/or xeric habitats, and thus could fully exploit these habitats as they expanded during the Eocene-Oligocene transition. This also may have facilitated movement across Beringia of the *rottenbergi* and Palearctic clades, a region which, following the transition, was dominated by mixed mesophilic forest [128]. Movement of the ancestor to the *salvini* clade into the Neotropics also occurred during the early Oligocene, where many of the contemporary taxa inhabit mesophilic forests, although some taxa are found primarily in dry forest or littoral scrub (pers. obs.).

*Temnothorax* appears to be one of several speciose Northern Hemisphere social insect lineages that originated or radiated in association with the Eocene-Oligocene transition: these include the cold-adapted *Bombus* bumblebees [129], *Myrmica* ants [130] and *Stenamma* ants  [46]. In each of these cases, the biogeographic history suggests a great fluidity of dispersal across Beringia, with 21 clades of *Bombus* exploiting this route starting in the Miocene (~20 Ma), several dispersal events in the case of *Myrmica* from the Oligocene onwards, and three Beringian dispersals in *Stenamma*, starting in the late Eocene. In the case of *Bombus*, dispersal is asymmetric, with Palearctic lineages moving to the Nearctic at higher rates; in *Stenamma*, two Nearctic-to-Palearctic events are inferred and one Palearctic-to-Nearctic; in the case of *Myrmica*, no formal biogeographic analysis was performed but it appears that dispersal occurred in both directions. Most of the dispersal in *Temnothorax* appears to be in the Nearctic-to-Palearctic direction, with one case of dispersal back to the Neactic occurring in the early Miocene, ~17 Ma in the case of the ancestor of *T. americanus* and the *longispinosus* group.

Global temperatures remained relatively stable until the late Oligocene, when a brief period of warming (ca. 24 Ma) was followed by another steep drop in deep ocean temperature, initiating a second period of glaciation at the Oligocene-Miocene boundary (23 Ma) [108], aridification [131, 132], and faunal turnover in at least some lineages [133]. Several lineages of arboreal *Temnothorax* arose near this transition, notably the Caribbean *sallei* subclade, the *obturator* clade, and members of the *salvini* clade, and continued to diversify throughout the Miocene. The causes behind this concerted pattern are obscure; epiphytic habits evolved repeatedly in the bromeliads during this era [134], and several Neotropical *salvini* clade species are associated with the genus *Tillandsia* [135]. Or, perhaps the abrupt drop in global temperatures opened a niche that was previously filled by a less cold-tolerant taxon.

Glaciation events continued throughout the Miocene, but intermittently and on a smaller scale than the event that marked the Oligocene-Miocene boundary [108]. The early Miocene was a period of generally warm temperatures, which slowly fell starting in the middle Miocene, when the Antarctic ice-sheet became a persistent global feature. Multiple lineages dispersed to the Caribbean during the Miocene, including the ancestors of the *sallei* and *iris* sublclades, the *pulchellu*s group, and members of the *obturator* clade. These events all occurred in the early Oligocene to middle Miocene, 18–30 Ma, and in most of these lineages the ancestral mainland range is inferred to be Nearctic, with the exception of the *pulchellus* group which had a Neotropical ancestral range. Because the evidence suggests that the Greater Antilles have not been connected to mainland North America since the early Eocene [136], and because by the Greater Antilles + Aves Ridge (GAARlandia) land span that connected northern South America to the developing northern Greater Antilles had subsided by the late Oligocene [57], an over-water route is the most plausible method of dispersal for all lineages present on the Caribbean islands today. Caribbean paleoceanographic hypotheses presented by Iturralde-Vinent [99] dovetail with an over-water dispersal route: there appears to have been a counterclockwise loop current in the Gulf of Mexico drawing water down from the Florida peninsula to the Greater Antilles, which has essentially been continually operating from the late Eocene up to the present. Dispersal has been in a single direction, from mainland to islands, but one species has expanded its range back to the subtropical Nearctic in recent times (*T. torrei*).

There also appears to have been fluidity across the Nearctic-Neotropical realms during the Miocene. In the *salvini* clade, *T. pergandei*, *T. sp. nr. peninsularis, T.* mmp12, and *T. subditivus* have traversed this boundary independently, dispersing from the Neotropics to the Nearctic. An interesting observation is that many of these species inhabit, or are tolerant of, dry forest or littoral scrub habitats, which may hold the key to understanding their apparent mobility across the Neotropic-Nearctic realms. In the *sallei* and *obturator* clades, *T*. mmp11*, T.* cf. *striatulus, T. striatulus*, and *T.* mmp08 currently inhabit mesophilic forests in the Neotropics, from mid-to-high elevations. *Temnothorax* mmp08 is only known from the Sierra Morena in Chiapas, Mexico, while the other species are more widespread, ranging from the Sierra Madre del Sur in southern Mexico to Honduras. Given the habitat affinities of these taxa, it may be that these represent relictual *sallei* clade taxa, protected from episodic cooling and warming since the middle Miocene by the complex topography of Mesoamerica.

### Ancestral state estimation

The ancestor of the crown *Temnothorax* is inferred to have had terrestrial nesting behavior, supporting the Bernard [31] hypothesis. The coordinated transitions to arboreal nesting behavior in several clades in the Oligocene are striking, but the mechanism driving this pattern remains unclear. Perhaps the late Oligocene warming facilitated movement into arboreal habitats; alternatively, early Miocene cooling may have increased competition among species for terrestrial nesting sites, with the more cold-tolerant *Temnothorax* species resorting to arboreal habitats. Within the ants, several genera, e.g. *Camponotus*, *Crematogaster*, *Monomorium* contain arboreal and ground-nesting species, but the history of transitions between these states has not been formally estimated in any of these. In *Crematogaster*, though, it appears that the predominant direction of transition is the converse of *Temnothorax,* with the predominantly arboreal and tropical species transitioning to terrestrial nesting in the temperate zones [45].

**Fig. 5:**
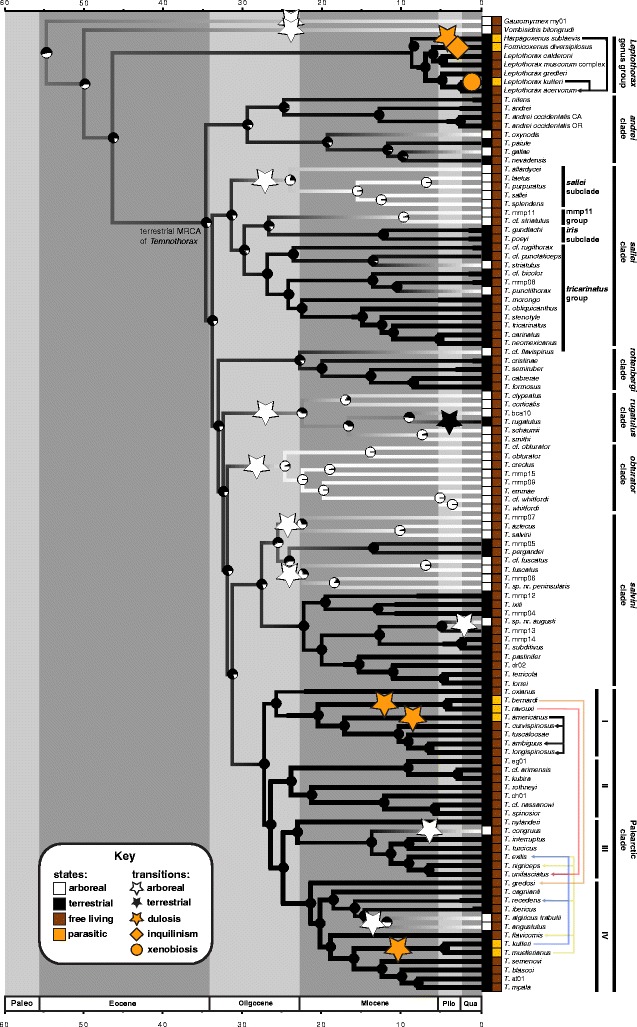
Results from ancestral state estimation of nesting habit and social parasitism in the core formicoxenines sensu Ward et al. [2], as inferred from an average of all models tested with HiSSE. Branches are painted according to transitions between arboreal (white) and terrestrial (black) nesting habitat. Pie diagrams at nodes indicate the likelihood of arboreal vs. terrestrial nesting habitat. Symbols on branches indicate transitions between states, as indicated in the key. Arrows between species indicate parasite/host relationships

In this study, parasitism is inferred to have evolved several times within *Temnothorax*, corroborating the findings of Beibl et al. [37]. Moreover, all of the parasitic taxa and hosts are confined to the Palearctic clade: the parasites are in subclades I and IV, while the hosts are scattered throughout subclades I, III, and IV (see Fig. 5). There appears to be support for both forms of Emery’s rule, but caution is needed when interpreting these results. *Temnothorax americanus* is the sister taxon to its hosts in the *longispinosus* group, but it is difficult to determine whether this is due to the ‘strict’ or ‘loose’ forms of Emery’s rule because a) the parasitic sister taxon is monospecific and b) the host species all have similar nesting habitats, typically within hollow twigs or nutshells in the leaf litter. Other species within the range of *T. americanus* are either parasites themselves (*T. duloticus, T. minutissimus*, and *T. pilagens*), have different ecological requirements (*T. schaumii* is arboreal, and *T. texanus* nests directly in the soil), or are distantly related (*T. pergandei*). Interestingly, *T. tuscaloosae* is not known to be parasitized by *T. americanus*, despite a range overlap in North Carolina.

*T. bernardi* and *T. ravouxi* of the *corsicus* group (formerly genus *Myrmoxenus*) are shown to be distantly related to their hosts in Palearctic subclades III and IV, suggesting the ‘loose form’ holds true in this case. Similarly, the *muellerianus* group (formerly genus *Chalepoxenus*) is the sister taxon to the *laurae* group; the parasitic *muellerianus* group taxa included here (*T. muellerianus* and *T. kutteri*) vary broadly with respect to their host species; but in all cases, they do not adhere to the ‘strict form’ of Emery’s rule. Recent phylogenetic studies of the *muellerianus* and *corsicus* species groups have elucidated the within-group relationships of these multi-species socially parasitic taxa [38]. Although more comprehensive sampling would be desirable to infer the crown ages of these groups, the current study includes the most distantly related *muellerianus* group taxa from Beibl et al. [38]. The results of the BEAST analysis suggest that these groups are young, with median ages of 4.1 and 4.4 Ma, respectively  (see Additional file 24: Table S14).

## Conclusions

This study represents the first effort to establish a time-calibrated phylogenetic framework for *Temnothorax*, elucidating a rich and intriguing history of evolution. Previous subgeneric and many species-group classification schemes were found to be unfounded with taxa distributed broadly among several morphologically convergent clades. *Temnothorax* arose in the Nearctic at the Eocene-Oligocene transition, ~35 Ma. The early evolution of the genus is distinguished by rapid radiation in a pectinate series followed by large-scale dispersal, with many of the major clades attaining their current geographic distributions by the early Miocene. Many arboreal lineages have a deep history dating back to the Oligocene, but they continued to arise in some clades throughout the Miocene. The ‘strict form’ of Emery’s rule for social parasites receives little support in the current study, although this may be case for *T. americanus* and other social parasites in the Palearctic I clade, namely *T. minutissimus*, *T. duloticus*, and *T. pilagens*. More thorough taxon sampling would further clarify the history of these interesting interactions. The new evolutionary framework presented here should simplify this task, having identified the phylogenetic context for the parasitic taxa and their hosts. Moreover, the evolutionary history of the Neotropical taxa is an exciting new frontier opened by this study. The convergence of two distantly related clades on the *Macromischa* syndrome and the radiation of *Temnothorax* on the Greater Antilles are intriguing features that beg further investigation.

## Additional files

Additional file 1: Extended background on the taxonomic history and fossil record of *Temnothorax* and relatives. (PDF 888 kb)

Additional file 2: Extended methods. (DOCX 193 kb)

Additional file 3: Table S1. *Temnothorax* species groups currently used in the literature, with references. (XLSX 32 kb)

Additional file 4: Table S2. List of specimens used in this study, with collection data, depository data and unique specimen identifiers. (XLSX 59 kb)

Additional file 5: Table S3. Primers used with Sanger sequencing in this study, with references. (XLSX 86 kb)

Additional file 6: Table S4. GenBank accession numbers associated with Sanger sequences generated or otherwise used in this study. (XLSX 63 kb)

Additional file 7: Trees inferred from the full Sanger sequencing dataset and data subsetting experiments. (PDF 437 kb)

Additional file 8: Table S5. Statistics of filtered UCE datasets used in this study. (XLSX 53 kb)

Additional file 9: Definitions and illustrations of morphological characters used in this study. (DOCX 101 kb)

Additional file 10: Table S6. Matrix of all morphological characters and indices used in this study, by taxon. (XLSX 50 kb)

Additional file 11: Table S7. List of *Temnothorax* species and core formicoxenines, coded for behavioral characteristics. (XLSX 49 kb)

Additional file 12: Table S8. Results of HiSSE model comparisons for the nesting habit and parasitism datasets. (XLSX 17 kb)

Additional file 13: Table S9. Sanger dataset summary statistics. (XLSX 44 kb)

Additional file 14: Table S10. The 14 partitions and substitution models identified by PartitionFinder and used in the maximum likelihood and Bayesian analyses of the concatenated Sanger sequencing dataset. (XLSX 35 kb)

Additional file 15: Trees inferred from single-gene Sanger sequencing analyses. (PDF 216 kb)

Additional file 16: Table S11. UCE assembly summary statistics. (XLSX 46 kb)

Additional file 17: Trees inferred from UCE dataset subsetting experiments. (PDF 460 kb)

Additional file 18: Chronograms inferred from calibration experiments. (PDF 636 kb)

Additional file 19: BI trees based on morphology, and morphology + molecular data. (PDF 75 kb)

Additional file 20: Table S12. Biogeographic model comparisons from BioGeoBEARS analyses. (XLSX 44 kb)

Additional file 21: Table S13. Biogeographic reconstructions at selected nodes, inferred with BioGeoBEARS using empirical data and sensitivity analyses. (XLSX 34 kb)

Additional file 22: Biogeographic reconstructions inferred from BioGeoBEARS anaylsis of empirical data and sensitivity analyses. (PDF 376 kb)

Additional file 23: Character dependent diversification rates and ancestral state reconstructions inferred with HiSSE models. (PDF 1361 kb)

Additional file 24: Table S14. Support values and 95% HPDs for select nodes across analyses. (XLSX 43 kb)

## Abbreviations

2lnBF: Bayes factor support
BI: Bayesian inference
BiSSE: Binary state speciation and extinction
bp: Nucleotide base pairs
BS: Maximum likelihood bootstrap support
CF: Core formicoxenines, sensu Ward et al. 2015 [2], consisting of the genera *Formicoxenus*, *Gauromyrmex*, *Harpagoxenus*, *Leptothorax, Temnothorax,* and *Vombisidris*.
HiSSE: Hidden state speciation and extinction [100]
HPD: Highest posterior density
Ka: Thousand years
LGG: *Leptothorax* genus group, consisting of the genera *Formicoxenus*, *Harpagoxenus*, and *Leptothorax*.
LPTM: Late Paleocene Thermal Maximum [108]
Ma: Million years ago
ML: Maximum likelihood
MRCA: Most recent common ancestor
PIS: Parsimony informative sites
PP: Bayesian posterior probability
RCFV: Relative composition frequency variability
UCEs: Ultraconserved elements.
