# Supplementary material for: Insights into the evolution, biogeography and natural history of the acorn ants, genus Temnothorax Mayr (hymenoptera: Formicidae)
Source: BMC Evol Biol. 2017 Dec 13;17:250. doi: 10.1186/s12862-017-1095-8 (PMC5729518; doi:10.1186/s12862-017-1095-8)
Supplement: Supplementary file 1 — Extended background on the taxonomic history and fossil record of Temnothorax and relatives. (PDF 888 kb) [file 12862_2017_1095_MOESM1_ESM.pdf]

Additional file 1: extended taxonomic history and fossil record of *Temnothorax* and relatives

Taxonomic history

The genus *Leptothorax,* until recently [1], contained most of the species now classified as *Temnothorax*. Established by Mayr [2], the name *Leptothorax* was applied to several different Palearctic species that are now classified as either *Leptothorax* or *Temnothorax*, without a formally designated type species. In 1861, Mayr described the genus *Temnothorax* [3], using this name for a single species with a Mediterranean distribution, *T. recedens* [Figure 1d]. *Temnothorax* was synonymized with *Leptothorax* by Forel [4], but was variously considered a subgenus of *Leptothorax* or a valid genus by others [5-7]. In an effort to provide taxonomic stability for Mayr's original cconcept of *Leptothorax*, several authors made alternate type designations in parallel, each apparently unaware of the taxonomic changes that the others were proposing, inadvertently compounding the issue. Therefore, while Bingham [8] and Wheeler [9] nominated *L. acervorum* [Figure 2f] as the name bearing species for *Leptothorax*, Emery [10] selected the Palearctic species presently classified as *T. clypeatus* [Figure 1a]; this second genus-concept was then broadly accepted. Around the same time, Ruzsky [11] erected a genus to encompass *L. acervorum* and its close relatives, the genus *Mychothorax,* which is often referred to as *Leptothorax sensu stricto* in the literature. The discrepancies between these parallel taxonomies were noted by M.R. Smith [12], who erected the subgenus *Myrafant* to encompass what he considered to be Emery's concept of *Leptothorax*, distinct from Ruzsky's *Mychothorax,* and subsequently nominated *T. curvispinosus* [Figure 1b], a common Nearctic species, as the type.

This classification scheme remained intact until 2003, when Bolton [1] updated the classification of the tribe Formicoxenini, which in his treatment consisted of twenty-two genera: *Atopomyrmex*, *Chalepoxenus* (= *Temnothorax* [1]*), Cardiocondyla*, *Dilobocondyla*, *Formicoxenus*, *Gauromyrmex*, *Harpagoxenus*, *Leptothorax*, *Myrmoxenus* (= *Temnothorax* [1]*), Nesomyrmex*, *Ochetomyrmex*, *Peronomyrmex*, *Podomyrma*, *Poecilomyrma*, *Protomognathus* (= *Temnothorax* [1]), *Romblonella*, *Rotastruma*, *Stereomyrmex*, *Temnothorax*, *Terataner*, *Vombisidris*, and *Xenomyrmex*. Furthermore, Bolton [1] presented morphological evidence for delineating *Temnothorax* and *Letpothorax s.s.*, observing that the latter is cleanly separated from the former by the presence of a transverse crest on the maxillary stipes [Figure 3b]. As a result, most *Leptothorax* species were transferred to *Temnothorax.* Additionally, Bolton [1] transferred many species to *Nesomyrmex* [Figure 2b] based on clypeal morphology, which in recent studies has been shown to be distantly related to these taxa [13, 14]. Presently, *Leptothorax* consists of nineteen species which vary only slightly from each other morphologically. They are distributed across the boreal habitats of the Northern hemisphere, often nesting in dead wood, under stones, or in close association with other ant species. Closely associated genera include the xenobiotic *Formicoxenus* [Figure 2d] and dulotic *Harpagoxenus* [Figure 2e].

Another former genus that contained many species presently classified as *Temnothorax* was *Macromischa*. Roger [15] established the genus *Macromischa* based, again, on a handful heterogeneous species without designating a type. Wheeler [9] corrected this by naming *T. purpuratus*, the first name appearing in the original article, as the name-bearing species [Figure 4c]. The former genus-or subgenus *Macromischa* was broadly applied to both Baltic amber fossils and extant Neotropical species of *Temnothorax* bearing a peculiar morphological syndrome. The primary diagnostic features of the *Macromischa* syndrome are a thin, elongate

petiole combined with a broad, campaniform post-petiole in dorsal view and a dorsally contiguous, often rounded, mesosoma in profile. Some additional characters that are associated with this syndrome, but not consistently present, are enlarged, spindle-shaped middle and hind femora, and iridescently colored integument. Mann [16] split *Macromischa* into three subgenera to accommodate some of the variation in the group, erecting the subgenera *Croesomyrmex* and *Antillaemyrmex*. Later, Brown [17] provisionally synonymized *Macromischa* with *Leptothorax*, but this name was still in use as a subgenus when Baroni Urbani [18] postulated that *Macromischa* was probably polyphyletic because many of the features that define this syndrome are found independently among clearly different lineages of *Temnothorax.* This taxon name fell out of use when it was formerly synonymized with *Leptothorax* by Snelling [19], who observed that there are many Central American species that are morphologically intermediate between *Leptothorax* and *Macromischa*.

Several subgenera have been proposed specifically for *Temnothorax:* Emery [20] erected the subgenus *Dichothorax* to accommodate the morphologically aberrant ground-nesting Nearctic species *T. pergandei* [Figure 1c]. This species differs from all others in the Nearctic region by its strongly depressed propodeum, massive, rounded promesonotum, and elongate petiole. Several authors have noted that this morphology is also present in several species around the Palearctic Mediterranean and North Africa, namely *T. recedens* [Figure 1d] and *T. schaufussi* [21, 22]. Ashmead [22] raised *Dichothorax* to genus level, but this change was not widely adopted; Hamman & Klemm [23] proposed *Icothorax* for the African species *T. megalops* [Figure 1e]; both names were used as subgenera until Bolton [24] synonymized them with *Leptothorax*. Similarly, Menozzi [25] created the subgenus *Myrmammophilus* to accommodate the Mediterranean *T. finzii* [Figure 1f] because the reproductives lacked a discoidal cell in their forewing, a feature that, in the light of the present study, appears to be plastic within *Temnothorax* and the LGG. This species is not particularly morphologically divergent when the diversity of North African species is considered, and appears to bear close affinities to the *laurae* species group, although it was placed in the *recedens*-group by Salata & Borowiec [26].

As the passages above indicate, the taxonomic history of *Temnothorax* is convoluted: senior synonym of no less than thirteen proposed genera and subgenera, the current definition was only arrived at in 2015 [13], when the species of the erstwhile socially parasitic genera *Chalepoxenus* [Figure 4d], *Myrmoxenus* [Figure 4b] and *Protomognathus* [Figure 4a] were synonymized with *Temnothorax* based on molecular evidence (see [27, 28] for an extensive discussion). Bolton [1, 24] did the bulk of the work updating *Temnothorax* to its modern definition, however, by formally designating the names *Antillaemyrmex*, *Croesomyrmex*, *Dichothorax*, *Icothorax, Macromischa, Myrafant,* and *Myrmammophilus* as junior synonyms of *Leptothorax* and later *Temnothorax*. Moreover, Bolton [24] provided morphological diagnoses to differentiate *Temnothorax*, the LGG, and the morphologically convergent *Nesomyrmex* [1, Figure 2b] based on mouthpart morphology.

Several large-scale species-level taxonomic revisions have been conducted in recent history: Mackay [29] resurrected and revised the North American subgenus *Myrafant* (i.e. all new world *Temnothorax* species excluding *Macromischa*, *Dichothorax* and *Protomognathus*), and Baroni Urbani [18] revised *Macromischa*, which contains most of the Neotropical species. Similar efforts have been made for the Palearctic taxa, but on a considerably finer geographical scale (e.g. [5, 29-39). This genus appears to be most species-rich in the Palearctic region, where there are currently 250 described species, as opposed to the 131 known from the Americas (including the Neotropics) and seven described from the depauperate Afrotropical fauna [40].

The disproportionate number of species among these biogeographic areas may be the product of several factors, namely the lack of a large-scale systematic synopsis in the Palearctic, where the large number of small-scale regional revisions has likely inflated the number of species due to undetected synonymies, and the relative paucity of collection efforts in Northern and Central Mexico where *Temnothorax* diversity is likely to be very high based on the number of undescribed species from southern Arizona (Stefan Cover, pers. comm.). Additionally, recent collecting initiatives in Central America, the Baja California peninsula, and the Dominican Republic have recovered many new species, which are currently in the process of being formally described (Prebus in prep.). Many of the authors cited above have offered informal species-group descriptions, but a formal systematic synthesis has not yet been attempted for any biogeographical region, much less on a global scale.

Fossil record

The fossil record for *Temnothorax* includes one species from Dominican Amber (15-20 Ma) [41, 42, but see 43], six from Baltic Amber (38-47 Ma) [44-48], and eight undescribed species from various European late Eocene and late Oligocene amber deposits (23-47 Ma) [49-51]. *Temnothorax praecreolus* de Andrade 1992*,* from the Dominican amber, is known from a single worker that exhibits several of the features associated with the *Macromischa* syndrome, including an elongate petiole, a broad, campaniform post-petiole, and enlarged mid and hind femora. While somewhat dorso-ventrally compressed, this fossil still possesses a remarkable number of visible characters. I obtained this fossil for inspection from the Naturhistoriches Museum, Basel, Switzerland [Figure 5a]. Mayr [44] described four species of *Macromischa* and one *Leptothorax* species, *L. gracilis* from Baltic amber [Figure 5d]. Wheeler [53] recognized one of Mayr's *Macromischa* species as *Vollenhovia*, and erected a new genus for the remainder of them, *Nothomyrmica*. Wheeler did not explicitly state his reasoning for this transfer, implying that because these species possess only one of the features of the *Macromischa* syndrome, a pedunculate petiole, that this precludes them from placement in any known genus. Wheeler's [53] treatment also introduced four new fossil species of *Leptothorax* from Baltic amber, noting that several of these species bear a close resemblance to *L. acervorum* in general bauplan, but four of the five fossil species have 12-merous antennae, a condition not found in the extant *Leptothorax s.s.*. Bolton [1] transferred all five of the fossil *Lepthothorax* species to *Temnothorax*.

More recently, Dlussky & Radchenko [54] dismantled *Nothomyrmica*, transferring *N. petiolata* to *Temnothorax* [Figure 5b]*,* designating a new genus, *Eocenomyrma* [Figure 5c], for *N. rugostriata* and three newly described fossil species*,* and subsequently synonymized the remainder of *Nothomyrmica* with *Myrmica* [55]. Dlussky & Radchenko [54] state that *Eocenomyrma,* while clearly separable from *Temnothorax* by clypeal structure, is probably a member the tribe Formicoxenini (*sensu* Bolton [1]). This same treatment did not give an explicit reason for transferring *N. petiolata*, only stating that it is 'indistinguishable by all its characteristic features' from *Temnothorax,* without enumerating the characters that lead them to this conclusion. *Temnothorax petiolata* bears long propodeal spines, 12-merous antennae, a metanotal groove, and a pedunculate petiole. These features in combination do indeed suggest that this species cannot be excluded from the crown group of *Temnothorax*, but I am hesitant to make a positive placement due to the lack of information about mandibular dentition and mouthpart morphology. For example, *T. petiolata* also closely resembles extant species of the Indomalayan and Australasian genus *Vombisidris* Bolton [56], which recent molecular work has

shown to be a close relative of [*Temnothorax* + LGG] [13]. *Vombisidris* has unique mandibular morphology, with a large diastema between the preapical mandibular tooth and the basalmost three. I have not yet been able to personally inspect the Baltic Amber fossils of *Temnothorax*, although images of *T. gracilis* and *T. petiolata* are available on AntWeb (http://www.antweb.org) [Figure 5b and d]. In a personal communication with Phillip Barden at the American Museum of Natural History, I inspected images of a Baltic Amber worker specimen identified as *Temnothorax* which had 12 antennomeres and a crest on the maxillary stipes, a condition not known from any extant *Temnothorax* or LGG species [Figure 4d]. This casts considerable doubt on whether *Temnothorax* fossils from Baltic Amber have been accurately identified. Consequently, I conservatively treat all Baltic Amber fossils currently classified as *Temnothorax* as stem lineages of the clade [*Temnothorax* + LGG] until the mouthpart morphology of each described fossil species can be inspected.

Taxonomic results

The constrained analysis of the Sanger sequencing dataset shown in Figure [2] in the main article. *Temnothorax* is composed of seven well-supported major clades in a pectinate series, which display a high degree of biogeographic structure. Each clade generally corresponds to a clade in the unconstrained Sanger dataset analyses Additional File [3], which is noted at the beginning of the description. Support values are given in posterior probability (PP) from the MrBayes analysis, and in bootstraps (BS) from the IQTREE analysis.

andrei-clade

The exclusively western and southwestern North American *andrei*-clade forms the first group in the pectinate series, sister to all other crown *Temnothorax.* This clade is composed of the *andrei*-, *nitens*-, as well as *T. gallae* and *T. nevadensis* of the *tricarinatus*-group proposed by Mackay [29]. The *tricarinatus*-group is found to be paraphyletic: *T. nevadensis* and *T. gallae* are more closely related to *T. andrei* than they are to *T. tricarinatus*, which is nested within the *sallei*-clade (see below). *T. nitens* is found to be sister to the *andrei*-group, but with low support (PP 1; BS 68).

sallei-clade

The *sallei*-clade, found in the southern Nearctic, Neotropics and the Greater Antilles, is the next to branch off. This group shows within-clade geographic structure, with the *sallei* and *iris* subclades distributed among the Greater Antilles and, in the case of *T. allardycei,* southern Florida and the Bahamas. These Caribbean species were informally classified by Baroni Urbani [18] as being members of the *Macromischa allardycei*- (*T. allardycei*), *sallei*- (*T. laetus* and *T. sallei*), *splendens*- (*T. splendens*), *iris*- (*T. gundlachi*), and *versicolor*- (*T. poeyi*) species groups. The *sallei*-group appears to be paraphyletic, with members distributed between the *purpuratus*- and *salvini*-clades (see below). *T.* mmp11 and *T. cf. striatulus* form a third distinct group, known only from the cloud forests of Meso-America. The latter is, at first glance, indistinguishable from *T. striatulus*, but the comparative length of the petiolar peduncle and appendages neatly separate the two taxa. The fourth subgroup within the *purpuratus*-clade is the remainder of the *tricarinatus*-group proposed in Mackay [29] (*T. carinatus, T. neomexicanus, T. obliquicanthus, T. punctithorax, T. cf. rugithorax, T. stenotyle,* and *T. tricarinatus*), within which the *striatulus*- and at least one member of the *andersoni*- species groups arose. This group is broadly distributed

throughout the southern Nearctic, with at least two species (*T. striatulus* and *T.* mmp0*8*) in the cloud forests of Meso-America. Following the *purpuratus*-clade, two smaller clades emerge.

rottenbergi-clade

The first of the smaller clades is the Palearctic *rottenbergi*-clade, composed of members of the nominal species group *sensu* Cagniant & Espadaler [30] (*T. cabrerae, T. formosus,* and *T. semiruber*), which are large, typically ground-nesting species distributed throughout the Mediterranean region, including North Africa and the Canary Islands. Two other species with a Mediterranean distribution are found to be affiliated with the *rottenbergi*-group: *T. cf. flavispinus* of the arboreal *flavispinus*-group (*sensu* Cagniant & Espadaler [30]) and the ground-nesting *T. cristinae*, which has been proposed to be closely related to *T. exilis* [57].

rugatulus-clade

The second of the smaller clades is the *rugatulus*-clade, which contains a mixture of Palearctic and broadly distributed North American species. The arboreal species *T. clypeatus* and *T. corticalis* (*clypeatus*- and *corticalis*-groups *sensu* Radchenko [32]), which are both rarely collected but apparently widely distributed in the western Palearctic, are contained within this group. The nominal species of this clade is commonly encountered nesting under stones in montane western North America (*longispinosus* group *sensu* Mackay [29]), sister to an undescribed species collected from a mesic mountaintop habitat on the southern Baja California peninsula. *T. schaumii* (*schaumii*-group Mackay [29]) and *T. smithi* (*silvestrii*-group Mackay [29]) are somewhat rarely collected arboreal species from eastern North America. The *longispinosus*- and *schaumii*-groups are polyphyletic as originally described: *T. rugatulus* and *T. schaumii* are more closely related to each other than to the other members of the original groups, all of which are monophyletic and nested within the Palearctic-clade (see below).

salvini-clade

The Neotropical and Caribbean *salvini*-clade is the next group to emerge, followed by the *obturator* and Palearctic-clades. The members of the *salvini*-clade are morphologically and behaviorally diverse, including many arboreal species from Meso-America and the enigmatic ground-nesting eastern North American species *T. pergandei* (formerly subgenus *Dichothorax*). Species in this clade have been informally classified as members of the *hispidus*-group *sensu* Mackay [29] (*T. sp. nr. peninsularis*), the arboreal mainland Meso-American 'Macromischa' species of the *sallei*- species group (*T. aztecus, T. salvini* and *T. fuscatus*), and the primarily ground-nesting Meso-American and Caribbean *pulchellus*-group *sensu* Baroni Urbani [18] (*T. augusti, T. subditivus, T. terricola* and *T. torrei*).

obturator-clade

The *obturator*-clade is a small group encompassing the *obturator*- and *emmae*- groups *sensu* Mackay [29], and the *creolus*-group sensu Baroni Urbani [18]. All known species from this group are arboreal, nesting in dead twigs or galls on live trees. Additional undescribed species were recently collected from Puerto Rico and Meso-American cloud forest. The *obturator*- and *salvini*-clades are both broadly distributed, having ranges like that of the *purpuratus*-clade, with members in the southern Nearctic and the Neotropics, including the Greater Antilles.

Palearctic-clade

The Palearctic-clade, as implied by the moniker, is mostly confined to the Palearctic biogeographic realm, with two notable exceptions: the dulotic eastern Nearctic species *T. americanus* (formerly *Protomognathus*) and a group that contains many of its hosts (the remainder of the polyphyletic *longispinosus* and *schaumii*-groups, which will be referred to as the *longispinosus*-group below) are nested within this clade; additionally, the Afrotropical *T. mpala* is nested within the southern Palearctic *laurae*- species group (*sensu* Prebus [40]). The socially parasitic *corsicus*- (formerly genus *Myrmoxenus*) and *muellerianus*- (formerly genus *Chalepoxenus*) species groups are nested within the Palearctic-clade as well, and are sister to [*T. americanus* + *longispinosus*-group] and the *laurae*- species group, respectively. The Palearctic clade is composed of four subclades and is generally, with a few exceptions, ground nesting.

(I) The first subgroup contains the central Palearctic *T. oxianus* (*bulgaricus*-group *sensu* Radchenko [31]), the *corsicus*-group, *T. americanus* and the *longispinosus*-group.

(II) A group consisting of eastern and southern Palearctic species, containing *T. nassanowi* (*nassanowi*-group Radchenko [33]), *T. spinosior*, which is a member of the *congruus*-group proposed by Radchenko [34], *T. kubira* and *T. cf. arimensis* from Japan, and *T. rothneyi* from the Himalyas. Two undescribed species are inferred to be members of this subgroup as well: *T.* eg01, collected from southeastern China and norther Vietnam, and *T.* ch01, from southeastern China.

(III) The third subgroup of the Palearctic-clade comprises species broadly distributed across the Palearctic, including *T. nylanderi* (*nylanderi*-group sensu Radchenko [33]), the arboreal *T. congruus* (*congruus*-group *sensu* Radchenko [34]), two members of the *tuberum*-group *sensu* Cagniant & Espadaler [30] (*T. nigriceps* and *T. unifasciatus*), *T. interruptus* of the *unifasciatus*-group *sensu* Bernard [5], *T. exilis* of the *exilis*-group, and *T. turcicus incertae sedis*. The *tuberum*- and *exilis*-groups as most recently described appear to be polyphyletic, with members distributed between this subclade and the Palearctic IV subclade.

(IV) The last subgroup of the Palearctic-clade contains many species with a circum-Mediterranean, southern Palearctic, and Afrotropical distribution, including members of the apparently polyphyletic *exilis*-group (*T. gredosi* and *T. ibericus*), the *nigritus*-group *sensu* Cagniant & Espadaler [30] (*T. cagnianti*), the recedens-group *sensu* Cagniant & Espadaler [30] (*T. recedens*), the polyphyletic *tuberum*-group (*T. algiricus trabutii*), the *angustulus*-group *sensu* Cagniant & Espadaler [30] (*T. angustulus*), the *flavicornis*-group *sensu* Bernard [5] (*T. flavicornis*), the former genus *Chalepoxenus,* and members of the *laurae*-group *sensu* Prebus [40] (*T. blascoi* and *T. mpala*).

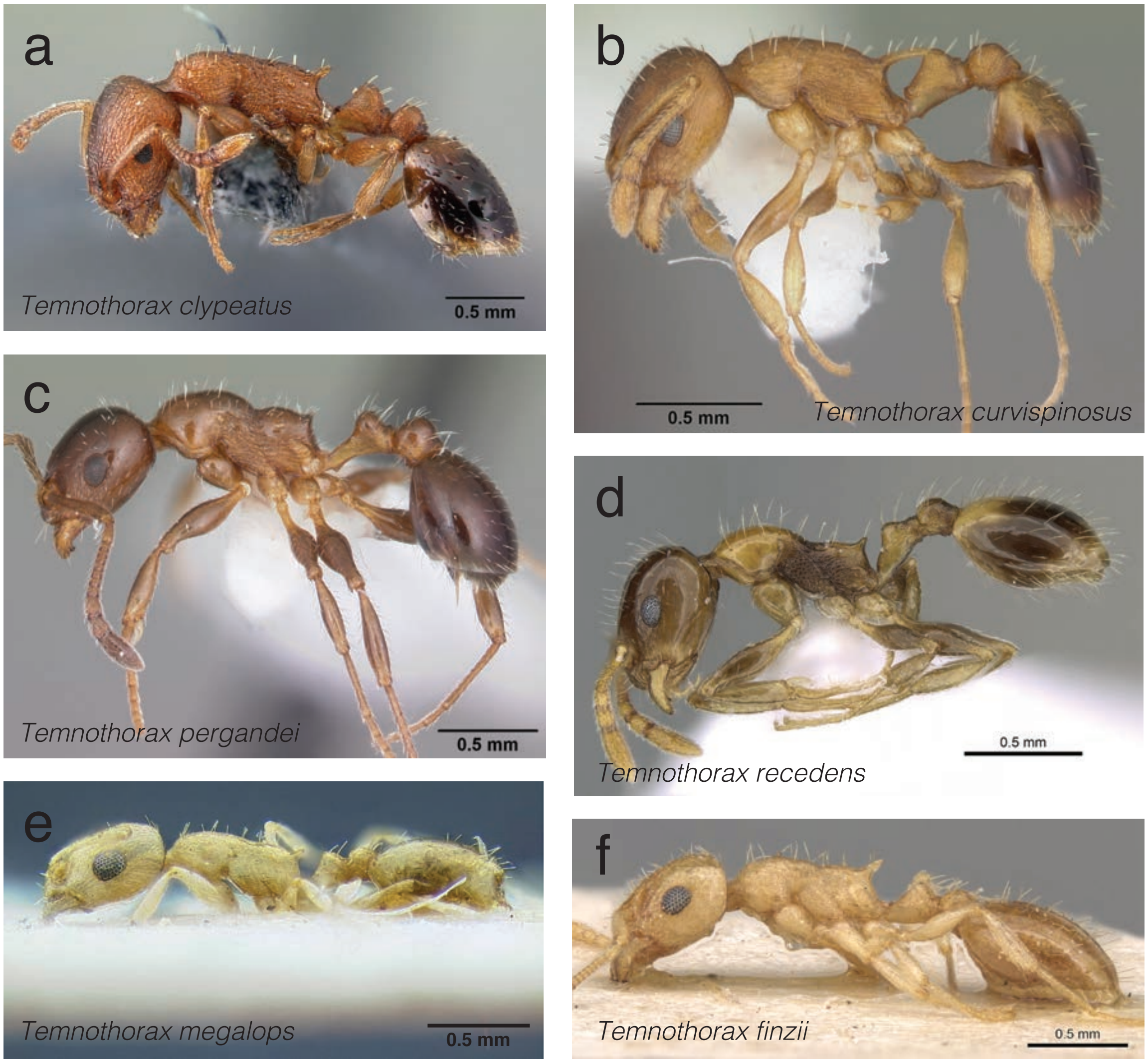

Figure 1. Exemplars of the former subgenera of *Temnothorax* discussed in the text. All images can be found on antweb.org (http://www.antweb.org) (a) *Temnothorax clypeatus* Mayr 1853, worker, profile view, CASENT0179903, photo: Erin Prado. (b) *Temnothorax curvispinosus* Mayr 1866, worker, profile view, CASENT0104040, photo: April Nobile. (c) *Temnothorax pergandei* Emery 1895, worker, profile view, CASENT0104016, photo: April Nobile. (d) *Temnothorax recedens* Nylander 1856, worker, profile view, CASENT0281561, photo: Estella Ortega. (e) *Temnothorax megalops* Hamann & Klemm 1967, worker, profile view, CASENT0712601, photo: Matthew Prebus. (f) *Temnothorax finzii* Menozzi 1925, worker, profile view, CASENT0904766, photo: Will Ericson.

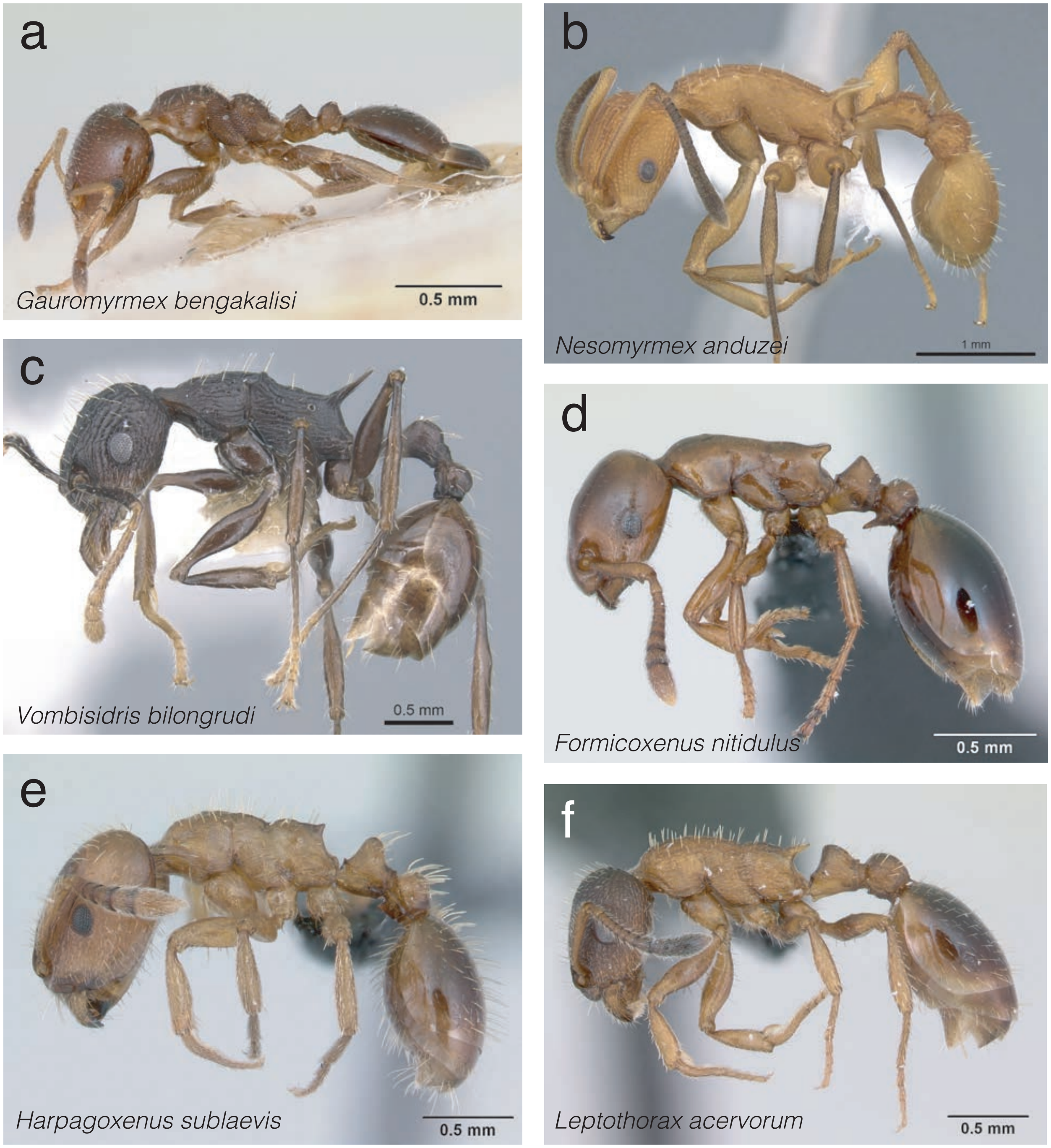

Figure 2. Exemplars of some of the outgroups used in the study, and *Nesomyrmex,* a genus that is morphologically convergent with *Temnothorax.* All images can be found on antweb.org (a) *Gauromyrmex bengakalisi* Menozzi 1933, worker, profile view, CASENT0172783, photo: April Nobile. (b) *Nesomyrmex anduzei* Weber 1943, worker, profile view, CASENT0914984, photo: Zach Lieberman. (c) *Vombisidris bilongrudi* Taylor 1989, worker, profile view, CASENT0901985, photo: Will Ericson. (d) *Formicoxenus nitidulus* Nylander 1846, worker, profile view, CASENT0173159, photo: April Nobile. (e) *Harpagoxenus sublaevis* Nylander 1849, worker, profile view, CASENT0178772, photo: April Nobile. (f) *Leptothorax acervorum* Fabricius 1793, worker, profile view, CASENT0173138, photo: April Nobile.

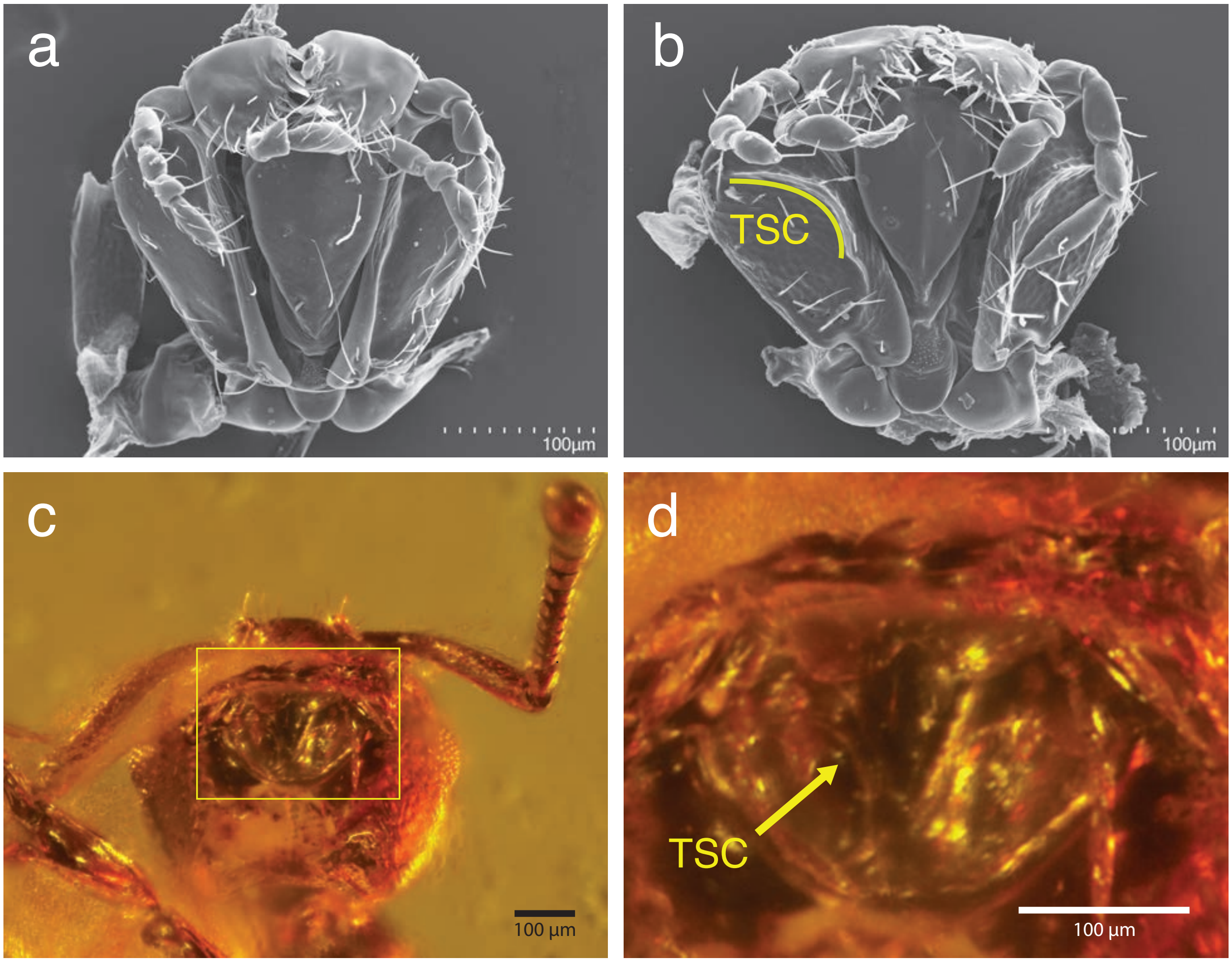

Figure 3. Mouthparts of core formicoxenines *sensu* Ward et al. (2016), illustrating absence (a) and presence (b-d) of the transverse crest of the maxillary stipes (TSC). (a, b) scanning electron micrographs of mouthpart dissections: (a) *Temnothorax cf. flavispinus*, photo: Matthew Prebus (b) *Formicoxenus diversipilosus,* photo: Matthew Prebus. (c, d) light microscope images showing the head capsule venter of a Baltic amber specimen AMNH Baltic 40129-511, photo: Phillip Barden: (d) is a magnification of the area highlighted in yellow in (c).

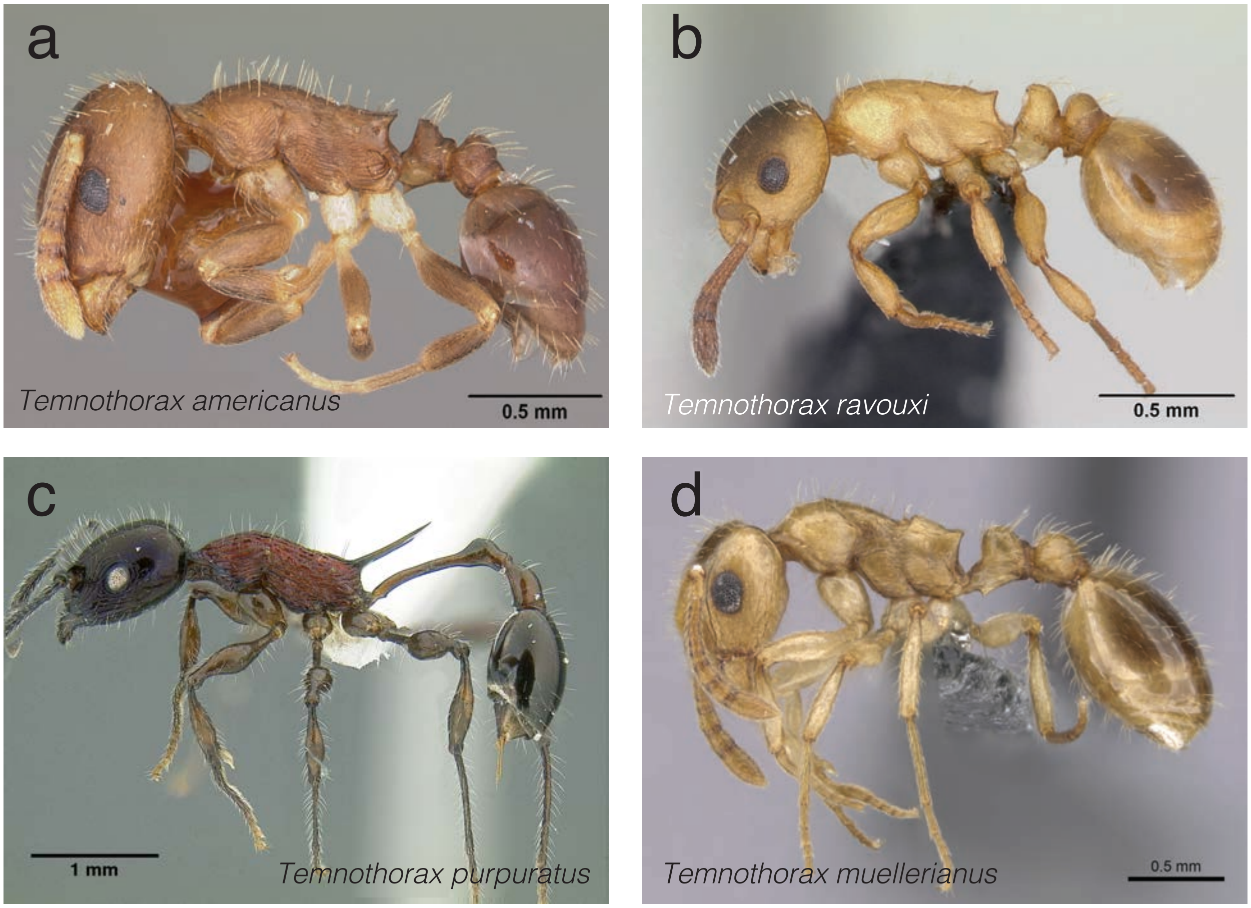

Figure 4. Exemplars of *Temnothorax* social parasites and the former subgenus *Macromischa.* All images can be found on antweb.org (a) *T. americanus* Emery 1895, worker, profile view. CASENT0104553, photo: April Nobile. (b) *T. ravouxi* André 1896, worker, profile view, CASENT017641, photo: April Nobile. (c) *T. purpuratus* Roger 1863, worker, profile view, CASENT0732599, photo: Matthew Prebus. (d) *T. muellerianus* Finzi 1922, worker, profile view, CASENT0270722, photo: Shannon Hartman.

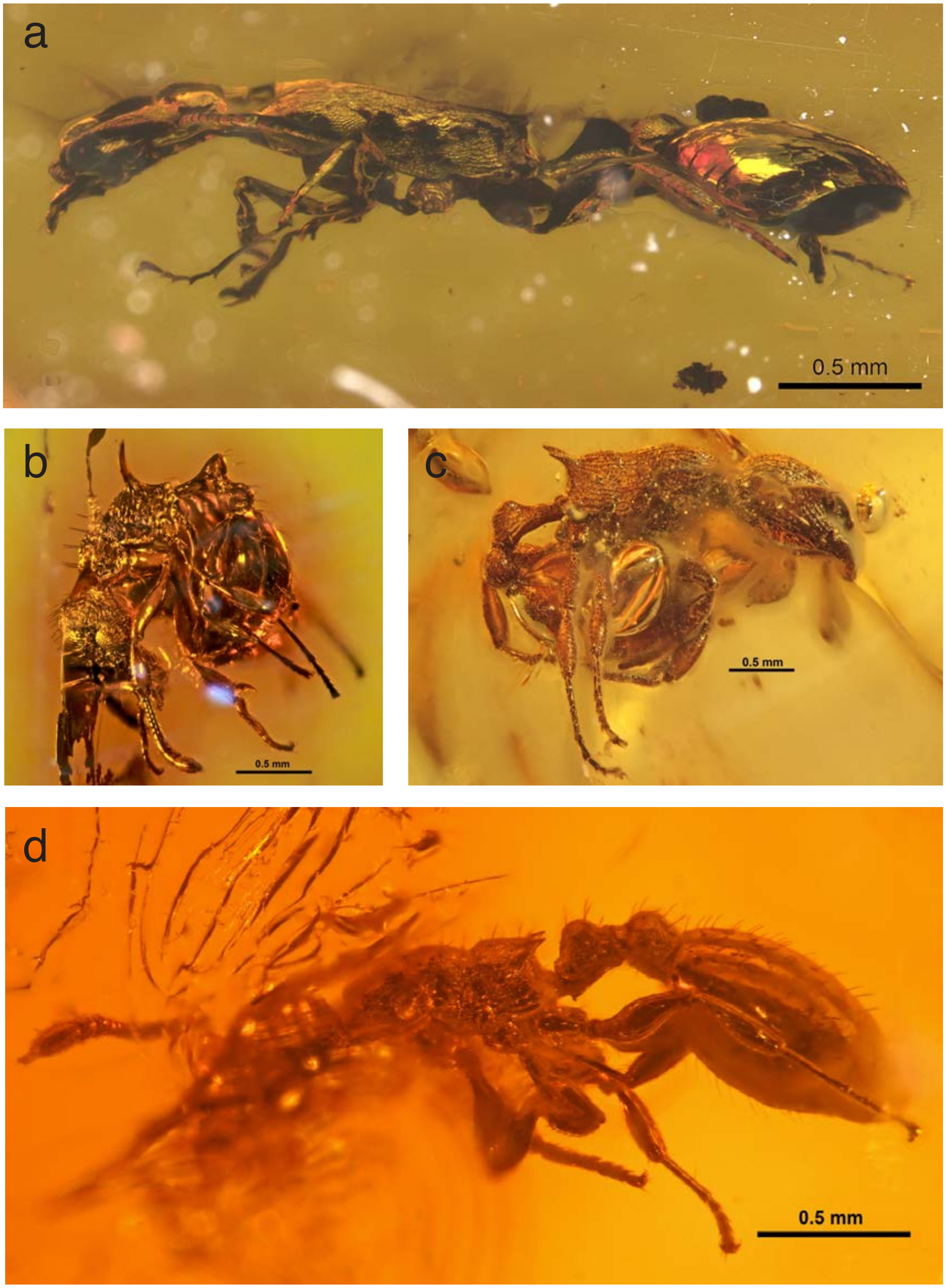

Figure 5. Images of fossil core formicoxenines *sensu* Ward et al. (2016). All images are found on antweb.org (a) *Temnothorax praecreolus* de Andrade 1992, worker, profile view, CASENT0915320, photo: Zach Lieberman. (b) *T. petiolata* Mayr 1868. Profile view, MBIGK001, photo: Vincent Perrichot. (c) *Eocenomyrma elegantula* Dlussky & Radchenko 2006, profile view, GPIH-BE4505, photo: Vincent Perrichot. (d) *T. gracilis* Mayr 1868, profile view, GZG-BST04661, photo: Vincent Perrichot.

References

1. Bolton B. Synopsis and classification of Formicidae. American Entomological Institute 2003;71:1-361.
2. Mayr G. Formicina austriaca. Beschreibung der bisher im österreichischen Kaiserstaate aufgefundenen Ameisen, nebst Hinzufügung jener in Deutschland, in der Schweiz und in Italien vorkommenden Arten. Verh Zool-Bot Ver Wien. 1855;5:273-478.
3. Mayr G. Die europäischen Formiciden. Nach der analytischen Methode bearbeitet. Wien: C. Gerolds Sohn; 1861.
4. Forel A. Fourmis de Tunisie et de l'Algérie orientale. Ann Soc Entomol Belg. 1890;34:lxi-lxxvi.
5. Bernard F. Faune de l'Europe et du Bassin Méditerranéen. 3. Les fourmis (Hymenoptera Formicidae) d'Europe occidentale et septentrionale. Paris: Masson; 1967.
6. Arnol'di KV, Dlussky GM. Superfam. Formicoidea. 1. Fam. Formicidae - ants. In: Medvedev GS, editor. Keys to the insects of the European part of the USSR. Vol. 3. Hymenoptera. Part 1. Opredeliteli Faune SSSR 1978;119:519-56.
7. Dlussky GM, Fedoseeva EB. 1988. Origin and early stages of evolution in ants. In: Ponomarenko AG, editor. Cretaceous biocenotic crisis and insect evolution. Moskva: Nauka; 1988: pp. 70-144.
8. Bingham CT. The fauna of British India, including Ceylon and Burma. Hymenoptera, Vol. II. Ants and Cuckoo-wasps. London: Taylor and Francis; 1903.
9. Wheeler WM. A list of the type species of the genera and subgenera of Formicidae. Ann N Y Acad Sci. 1911;21:157-75.
10. Emery C. Les espèces-type des genres et sous-genres de la famille des Formicides. Ann Soc Entomol Belg. 1912;56:271-3.
11. Ruzsky M. On ants from Archangel province. Zap Imp Rus Geogr Obshch Obshch Geogr. 1904;41:287-94.
12. Smith MR. On the status of *Leptothorax* Mayr and some of its subgenera. Psyche. 1950;57:29-30.
13. Ward PS, Brady SG, Fisher BL, Schultz TR. The evolution of myrmicine ants: phylogeny and biogeography of a hyperdiverse ant clade (Hymenoptera: Formicidae). Syst Ent. 2015;40:61-81.
14. Brady SG, Schultz TR, Fisher BL, Ward PS. Evaluating alternative hypotheses for the early evolution and diversification of ants. Proc Natl Acad Sci. 2006;103:18172-7.
15. Roger J. Die neu aufgeführten Gattungen und Arten meines Formiciden-Verzeichnisses nebst Ergänzung einiger früher gegebenen Beschreibungen. Dtsc Entomol Z. 1863;7:131-214.
16. Mann WM. Additions to the ant fauna of the West Indies and Central America. Bull Am Mus Nat Hist. 1920;42:403-39.
17. Brown WL Jr. A comparison of the Hylean and Congo-West African rain forest ant faunas. In: Meggers BJ, Ayensu ES, Duckworth WD, editors. Tropical forest ecosystems in Africa and South America: a comparative review. Washington, D.C.: Smithsonian Institution Press; 1973. pp. 161-85.
18. Baroni Urbani, C. Materiali per una revisione dei *Leptothorax* neotropicali appartenenti al sottogenere *Macromischa* Roger, n. comb. (Hymenoptera: Formicidae). Ent Basiliensia. 1978;3:395–618.

19. Snelling RR. 1986. New synonymy in Caribbean ants of the genus *Leptothorax* (Hymenoptera: Formicidae). Proc Entomol Soc Wash. 1986;88:154-6.
20. Emery C. Beiträge zur Kenntniss der nordamerikanischen Ameisenfauna. (Schluss). Zool Jahrb Abt Anat Ontog Tiere. 1895;8:257-360.
21. MacKay WP. The status of the ant *Leptothorax pergandei* Emery (Hymenoptera: Formicidae). Sociobiology. 1993;21:287.
22. Ashmead WH. A skeleton of a new arrangement of the families, subfamilies, tribes and genera of the ants, or the superfamily Formicoidea. Can Entomol. 1905;37:381-4.
23. Hamann HHF, Klemm W. Ergebnisse der zoologischen Nubien-Expedition 1962. Teil XXXIV. Hymenoptera - Formicidae. Ann Nat Hist Mus Wien. 1967;70: 411-21.
24. Bolton B. Afrotropical species of the myrmicine ant genera *Cardiocondyla, Leptothorax, Melissotarsus, Messor* and *Cataulacus* (Formicidae). Bull Br Mus Nat Hist (Ent). 1982;45:307-70.
25. Menozzi C. Res mutinenses. Formicidae (Hymenoptera). Atti Soc Nat Mat Modena. 1925;55:22-47.
26. Salata S, Borowiec L. Redescription of *Temnothorax antigoni* (Forel, 1911) and description of its new social parasite *Temnothorax curtisetosus* sp. n. from Turkey (Hymenoptera, Formicidae). ZooKeys. 2015;523:129-48.
27. Seifert B, Buschinger A, Aldawood A, Antonova V, Bharti H, Borowiec L, Dekoninck W, Dubovikoff D, Espadaler X, Flegr J, Georgiadis C, Heinze J, Neumeyer R, Ødegaard F, Oettler J, Radchenko A, Schultz R, Sharaf M, Trager J, Vesnić A, Wiezik M, Zettel H. Banning paraphylies and executing Linnaean taxonomy is discordant and reduces the evolutionary and semantic information content of biological nomenclature. Insectes Soc. 2016;63:237-42.
28. Ward PS, Brady SG, Fisher BL, Schultz TR. 2016. Phylogenetic classifications are informative, stable, and pragmatic: the case for monophyletic taxa. Insectes Soc. 2016;63:489-92.
29. MacKay WP. 2000. A review of the New World ants of the subgenus *Myrafant* (genus *Leptothorax*) (Hymenoptera: Formicidae). Sociobiology. 2000;36.
30. Cagniant H, Espadaler X. Les *Leptothorax*, *Epimyrma* et *Chalepoxenus* du Maroc (Hymenoptera: Formicidae). Clé et catalogue des espèces. Ann Soc Entomol Fr. 1997;33:259-84.
31. Radchenko AG. A review of the ant genus *Leptothorax* (Hymenoptera, Formicidae) of the central and eastern Palearctic. Communication 1. Subdivision into groups. Groups *acervorum* and *bulgaricus*. Vestn Zool. 1994;6:22-8.
32. Radchenko AG. A review of the ant genus *Leptothorax* (Hymenoptera, Formicidae) of the central and eastern Palearctic. Communication 2. Groups *tuberum*, *corticalis*, *affinis*, *clypeatus*, *alinae* and *singularis*. Vestn Zool. 1995;2-3:14-21.
33. Radchenko AG. A review of the ant genus *Leptothorax* (Hymenoptera, Formicidae) of the central and eastern Palearctic. Communication 3. Groups *nylanderi*, *korbi*, *nassonovi*, and *susamyri*. Vestn Zool. 1995;4:3-11.
34. Radchenko AG. A review of the ant genus *Leptothorax* (Hymenoptera, Formicidae) of central and eastern Palearctic. Communication 4. Group *congruus*. Species of uncertain position. Zoogeographical characteristics. Vestn Zool. 1996;1-2:16-22.

35. Radchenko AG. A review of the ant genera *Leptothorax* Mayr and *Temnothorax* Mayr (Hymenoptera, Formicidae) of the eastern Palaearctic. Acta Zool Acad Sci Hung. 2004;50:109-137.
36. Radchenko AG. Monographic revision of the ants (Hymenoptera: Formicidae) of North Korea. Ann Zool. 2005;55:127-221.
37. Bharti H, Gul I, Sasi S. Taxonomy of *Temnothorax simlensis* stat. nov. (Hymenoptera: Formicidae) with first description of sexual castes along with a mention of its plesiobiotic association with Himalayan species of genus *Myrmica*. Sociobiology. 2016;63:748-54.
38. Zhou S, Huang J, Yu D, Liu Z. Eight new species and three newly recorded species of the ant genus *Temnothorax* Mayr (Hymenoptera: Formicidae) from the Chinese mainland, with a key. Sociobiology. 2010;56:7-26.
39. Terayama M, Onoyama K. The ant genus *Leptothorax* Mayr (Hymenoptera: Formicidae) in Japan. Mem Myrmecol Soc Jpn. 1999;1:71-97.
40. Prebus, M. Palearctic elements in the old world tropics: a taxonomic revision of the ant genus *Temnothorax* Mayr (Hymenoptera, Formicidae) for the Afrotropical biogeographical region. ZooKeys. 2015;483:23-57.
41. de Andrade ML. First fossil "true *Macromischa*" in amber from the Dominican Republic (Hymenoptera, Formicidae). Mitt Schweiz Entomol Ges. 1992;65:341-51.
42. Iturralde-Vinent MA, MacPhee RD. Age and paleogeographical origin of Dominican amber. Science. 1996;273:1850.
43. Grimaldi DA. The age of Dominican amber. In: Anderson KB, Crelling JC, editors. Amber, Resinite, and Fossil Resins. American Chemical Society; 1995.
44. Mayr G. Die Ameisen des baltischen Bernsteins. Beitr Naturk Preussens. 1868;1:1-102.
45. Wheeler WM. The ants of the Baltic Amber. Schr Phys Ökonom Gesell Königsberg. 1915;55:1-142.
46. Ritzkowski S. K-Ar-Altersbestimmung der bernsteinführenden Sedimente des Samlandes (Paläogen, Bezirk Kaliningrad). Metalla (Sonderheft). 1997;66:19–23.
47. Kaplan AA, Grigelis AA, Strelnikova NI, Glikman LS. 1977. Stratigraphy and correlation of Palaeogene deposits of South-Western cis-Baltic region. Soviet Geol. 1997;4:30–43.
48. Perkovsky EE, Rasnitsyn AP, Vlaskin AP, Taraschuk MV. A comparative analysis of the Baltic and Rovno amber arthropod faunas: representative samples. Afr Invertebr. 2007;48:229-45.
49. Dlussky GM, Rasnitsyn AP. 2009. Ants (Insecta: Vespida: Formicidae) in the Upper Eocene amber of central and eastern Europe. Paleontol J. 2009;43:1024-42.
50. Perkovsky, EE. Tropical and Holarctic ants in Late Eocene ambers. Vestn Zool. 2016;50:111-22.
51. Knuth G, Koch T, Rappsilber I, Volland L. Concerning amber in the Bitterfeld region—geologic and genetic aspects. Hallesches Jahrb Geowiss. 2002;24:35-46.
52. Mayr G. Die Ameisen des baltischen Bernsteins. Beitr Naturk Preussens. 1868;1:1-102.
53. Wheeler WM. The ants of the Baltic Amber. Schr Phys Ökonom Gesell Königsberg. 1915;55:1-142.
54. Dlussky G, Radchenko A. A new ant genus from the late Eocene European amber. Acta Palaeontol Pol. 2006;51:561-7.

55. Radchenko AG, Dlussky G, Elmes GW. The ants of the genus *Myrmica* (Hymenoptera, Formicidae) from Baltic and Saxonian amber (Late Eocene). J Paleontol. 2007;81:1494-501.
56. Bolton B. New myrmicine ant genera from the Oriental Region Hymenoptera: Formicidae. Syst Ent. 1991;16:1-3.
57. Arcos J, Espadaler X, Catarineu C. Neuvas citas de *Temnothorax cristinae* (Espadaler 1997) para el sureste de la península ibérica y ampliación de su descripción. Iberomyrmex 2013;5:5-8.
