# Supplementary material for: Insights into the evolution, biogeography and natural history of the acorn ants, genus Temnothorax Mayr (hymenoptera: Formicidae)
Source: BMC Evol Biol. 2017 Dec 13;17:250. doi: 10.1186/s12862-017-1095-8 (PMC5729518; doi:10.1186/s12862-017-1095-8)
Supplement: Supplementary file 2 — Extended methods. (DOCX 193 kb) [file 12862_2017_1095_MOESM2_ESM.docx]

**Additional file 2: extended methods**

*Taxon sampling: Sanger dataset*

My taxon sampling strategy was designed to capture all the morphological, geographical, and life history variation within *Temnothorax*, with several exemplars chosen from each species group when possible. I selected 103 *Temnothorax* species, representing the recently synonymized satellite genera and 41 out of the 53 species groups currently in use, and 24 taxa of uncertain species-group affinity (see Additional table [1] for a synopsis of the species groups). For outgroups, I used seven exemplars of the *Leptothorax* genus group (LGG), as well as representative species of the Indomalayan and Australasian genera *Gauromyrmex* and *Vomibisidris,* all of which have been demonstrated to be close relatives of *Temnothorax* [1]. To provide additional calibration points for divergence dating analyses and for rooting the tree, I added several more taxa which encompass the phylogenetic diversity of the subfamily Myrmicinae. Voucher specimens used for DNA extractions in this study were deposited in the University of California, Davis Bohart Museum of Entomology collection; unique specimen identifiers and collection data for these can be found in Additional table [2]; additional data is available online at AntWeb (<http://antweb.org)> by using the specimen identifiers to search the database.

*Sequence generation: Sanger dataset*

DNA was extracted from ant specimens using the Qiagen DNeasy Blood & Tissue Kit (Qiagen, Inc.). To preserve the exoskeletons of extracted specimens as vouchers, all adult workers and queens used in this study were prepared for DNA extraction with a nondestructive method, in which the integument of the mesosoma and gaster on the right side of the specimen were punctured using a flame-sterilized 00-gauge insect pin. All specimens were processed using the standard protocol for the Qiagen DNeasy extraction kit, but I diverged from it at two points: specimens were left overnight (~12 hours) in the Proteinase-K solution during cell lysis, and DNA was eluted with sterilized water instead of the provided buffer in the final step.

Fragments of eight single-copy nuclear and two mitochondrial markers commonly used in ant systematics were chosen for amplification (for a complete list of primers used for amplification see Additional table [3]): 28S rDNA (*28S*), abdominal-A (*abdA*), arginine kinase (*argK*) including intron, elongation factor 1-alpha F2 (*EF1aF2*), long-wavelength rhodopsin (*LW Rh*) including intron, wingless (*wg*) including intron, rudimentary (*CAD*) including two introns, topoisomerase 1 (*Top1*), cytochrome c oxidase subunit I (*COI*) and cytochrome c oxidase subunit II (*COII*), including the spacer region between these two genes. Amplifications were performed using the PCR protocols described in Ward and Downie [2]. Sequencing was performed using BigDye Terminator v3.1 Cycle Sequencing chemistry, and amplicons were analyzed on an ABI 3730 Capillary Electrophoresis Genetic Analyzer (Life Technologies) at the College of Biological Sciences DNA Sequencing Facility, University of California, Davis.

*Processing and alignment: Sanger dataset*

Sequence base calling and primer trimming was performed with Sequencher v 5.2.2 (Gene Codes Corporation) and exported for alignment as FASTA files. Because it has been suggested that arbitrary choice of alleles can bias phylogenetic results in some data sets [3], I retained ambiguous base calls (R or Y) for any potentially heterozygous sites. Sequences of the outgroup taxa *Myrmica striolagaster, Solenopsis xyloni, Pogonomyrmex subdentatus, Tetheamyrma subspongia, Harpagoxenus sublaevis, Formicoxenus diversipilosus* and *Vombisidris bilongrudi* were retrieved from Genbank. GenBank identifiers for these and all new sequences generated by this study are listed in Additional table [4].

*Phylogenetic inference: Sanger dataset*

Because intron sequence in the outgroup taxa diverged strongly from the ingroup, I maximized the number of informative sites for ingroup taxa by removing the introns from all outgroup taxa prior to alignment, and retained introns only in *Temnothorax*. Sequences were aligned with MAFFT v7.273 using the L-INS-I algorithm [4], which has been demonstrated to outperform other alignment programs in terms of accuracy [5-7]. Alignment, codon positions, and intron locations were verified by eye using Mesquite v 3.04 [8]. Alignment confidence values for all sites in non-coding sequence were generated with the program ZORRO [9] and sites that fell below an arbitrary threshold of five were trimmed from the alignment using a custom Python script developed by Fernández *et al.* [10]. Chi-squared test for nucleotide homogeneity, summary and base composition statistics were calculated with BaCoCa v 1.1.r [11]. Because the third codon position of *COI + COII* failed the chi-squared test (P < 0.01); it was RY-coded with Mesquite for downstream analyses.

The assumption that nucleotide substitution rate is homogenous across all sequences is often violated in empirical data [12]; therefore, I partitioned the concatenated matrices into blocks corresponding to each of the three codon positions of each protein-coding gene, introns of each protein coding gene, and 28S rDNA. I then ran PartitionFinder 1.1.1 [13] to group these blocks into sets of similarly evolving sites and estimate a best fitting substitution model for each set. I opted to link branch-lengths among partitions, and used the Bayesian information criterion (BIC) for model selection and the ‘*greedy*’ algorithm for best partitioning scheme selection. It has been noted that substitution models combining an estimation of the proportion of invariable sites and gamma-distributed rate variation (+I+G) may be confounded in certain circumstances [14-16]. For this reason, I set the ‘*models’* option to a subset of ‘*all’* which excluded the combination of these among-site rate variation parameters. The PartitionFinder analysis resulted in a best-scoring scheme with fourteen partitions (see Additional table [10]).

Two programs were used for maximum likelihood (ML) inference, RAxML [17] and IQ-TREE [18]. RAxML, which has a smaller range of substitution models options than IQ-TREE, was used to investigate the effect of introns and the rapidly evolving *COI + COII* genes on tree inference. I created four data subsets to compare the effects of these types of data on the tree topology:

1. all data
2. introns excluded
3. *COI + COII* excluded
4. introns, *COI* + *COII* excluded

I used the best partitioning scheme generated by PartitionFinder, the GTR+GAMMA substitution model, and 200 rapid bootstrap replicates to calculate a phylogeny for each matrix in RAxML.

To investigate the effect of the full spectrum of substitution models suggested by ParitionFinder on tree inference, I used the program IQ-TREE on the full ten gene dataset, employing the partitioning scheme and substitution models selected by PartitionFinder and 200 standard non-parametric bootstrap replicates. For both the RAxML and IQ-TREE approaches, I summarized the runs with the best tree annotated with bootstrap support at each node.

For Bayesian inference (BI), I used MrBayes 3.2.6 [19] as implemented on CIPRES (<http://www.phylo.org/> [20]). Instead of relying on the limited number of named substitution models implemented in MrBayes, I used the reversible jump Markov chain Monte Carlo algorithm (rj-MCMC, called by setting *nst = mixed*) to sample across model space*,* specifying among-site rate variation parameters for each partition as suggested by PartitionFinder. State frequencies, substitution rates, gamma shape and proportion of invariable sites were unlinked and allowed to differ across partitions. I ran MrBayes for 50 million generations, with two independent runs, four chains*,* sampling parameters every 5000 generations. For comparison, I also ran an analysis using the above settings, but employed the substitution models suggested by PartitionFinder instead of the rj-MCMC algorithm.

To investigate the possibility of gene tree-species tree conflict, I also ran single-marker analyses in MrBayes. For these analyses, I used the same settings as for the rj-MCMC concatenated analysis, but reduced the number of generations and sampling frequency to 10 million and 1000, respectively.

To diagnose MCMC convergence, I examined the following statistics for each run in the MRBAYES log file: potential scale reduction factor (PRSF) values, which should be near 1.0, and average standard deviation of split frequencies between runs, which should be below 0.05. I also inspected trace plots and estimated sample size (ESS), which should be >200 with the program Tracer v1.6 [21] for signs of adequate parameter mixing. Additionally, I ran all analyses without data to confirm that the priors were not adversely influencing the results. I summarized each analysis with a consensus tree after discarding the initial 25% of trees as burnin.

Because the Bayesian and ML trees resulting from analysis of full dataset only differed topologically from the reduced datasets at nodes that were poorly supported in both analyses, I selected the full dataset for downstream analyses.

To investigate the possibility of gene tree conflict in the full dataset, I used the program BUCKy 1.4.4 [21, 22] to conduct a Bayesian concordance analysis, using the individual gene trees generated in the MRBAYES analysis above. I first summarized the tree files using the ‘mbsum’ (which is distributed as part of the BUCKy software package), discarding the first 2500 trees from each file as burn-in. The output from the tree file summary was then processed with BUCKy to generate a primary concordance tree with concordance factors for each clade. I changed several settings from the program defaults: I set alpha (the *a priori* level of gene tree discordance) to 5, increased the number of generations to 1 million, increased the number of runs to 4, and implemented 3 heated chains in addition to the cold chain.

*Taxon sampling: UCE dataset*

Because the phylogeny inferred with Sanger sequencing data showed little support for deep relationships within the genus (see Additional file [3]), I selected several exemplars of each of the ten well supported lineages and four subclades of the Palearctic clade for further analysis with UCEs. In total, I used 13 outgroup and 37 ingroup species in an effort to resolve the poorly supported backbone of the phylogeny.

*Sequence generation: UCE dataset*

I used the following protocols, which are based on those written by Brant Faircloth (available from <http://www.ultraconserved.org)> and Faircloth *et al.* [24] to enrich and sequence UCEs. These basic protocols were adjusted following Branstetter *et al.* [25] in the following ways: the Kapa Hyper Prep kit (Kapa Biosystems) and the iTru dual-indexing adapter system (Glenn *et al*. in prep.) were used during library preparation, and iTru blockers were used during the enrichment process. Furthermore, during the post-enrichment PCR step, DNA was left bound to the streptavidin beads, and purifications were performed using a generic SPRI substitute [24, 26] (referred to here as “speedbeads”) at 1.1x.

I measured the DNA concentration of each extract using a Qubit 2.0 fluorometer (High Sensitivity kit, Life Technologies, Inc.) and input up to 50 ng of DNA into shearing and library preparation. I sheared DNA to a target fragment distribution of 400-600 bp using a Diagenode Bioruptor sonicator (Diagenode Inc.). For fresh samples (< 25 years since the specimen was originally collected), which were expected to have relatively intact DNA, I ran a program that sonicated 100 µL samples for four cycles of 15 seconds on and 90 seconds off. For older samples (> 25 years since collection), I sonicated the extract for one cycle of 15 seconds, with a rest of 90 seconds.

Following sonication, I constructed sequencing libraries using the Kapa Hyper Prep Kit (Kapa Biosystems). I performed all library preparation reactions at ¼ of the volume suggested by the manufacturer protocol except for the PCR step, which I assembled at full volume (50 μL). During the adapter ligation step, I used iTru dual-indexing adapter-primers, as opposed to the analogous Illumina TruSeq HT system. At the PCR amplification step, I input 15 μL DNA, 25 μL HiFi HotStart Ready Mix (Kapa Biosystems), 2.5 μL each i5 and i7 iTru primers (5 μL total), and 5 μL double distilled water (ddH_2_O). I used the following thermal cycler protocol: 98ºC for 45 seconds; 12 cycles of 98ºC for 15 seconds, 60ºC for 30 seconds, 72ºC for 60 seconds; and a final extension at 72ºC for 5 minutes. After speedbead cleanup, I rehydrated the PCR product in 23 μL Elution Buffer (EB), I verified library amplification with Qubit (Broad Range kit, Life Technologies, Inc.), and inspected fragment size distribution of the libraries on an agarose gel.

For UCE enrichment I made pools at equimolar concentrations containing 6-10 libraries and measured DNA concentration with a Qubit fluorometer (Broad Range kit). I adjusted pool concentrations to approximately 147 ng/μL using a vacuum centrifuge by dehydrating them completely and rehydrating them with the appropriate volume of water, calculated from the Qubit measurements. A total of 500 ng of DNA (3.4 μL) from each concentration-adjusted pool was input into the enrichment protocol. I performed enrichments using a custom RNA bait library (MYBaits kit) developed for Formicidae [28] and synthesized by MYcroarray (MYcroarray, Inc.), consisting of 9898 probes targeting 2524 UCE loci. I enriched each pool of samples following the protocol described in Faircloth *et al.* [24], except that I reduced the standard MYBaits concentration to 0.1X, used iTru adapter blockers instead of the standard MYcroarray blockers, and streptavidin beads were left bound to enriched DNA during PCR, as described in Faircloth *et al.* [24]. The enrichment was performed at 65˚C for a period of 24 hours, and all pools were subsequently bound to streptavidin beads (Dynabeads MyOne Streptavidin T1, Invitrogen), and washed according to Blumenstiel *et al.* [28]. I combined 15 μL of bead-bound enriched library with 25 μL HiFi HotStart Ready Mix (Kapa Biosystems), 5 μL of Illumina TruSeq Primer Mix (5μM each forward and reverse primers), and 5 μL ddH_2_O. I ran post-enrichment PCR amplification using the following thermal cycler protocol: 98ºC for 45 seconds; 18 cycles of 98ºC for 15 seconds, 60ºC for 30 seconds, and 72ºC for 60 seconds; and a final extension of 72ºC for 5 minutes. I cleaned the resulting enriched, amplified pools using 1.1X speedbeads, and eluted the DNA from the speedbeads in 22 μL EB. Finally, I quantified DNA concentration using a Qubit fluorometer (Broad Range kit).

I verified enrichment success with relative qPCR by comparing amplification profiles of unenriched to enriched pools for seven UCE loci included in the probe set, using a SYBR® FAST qPCR kit (Kapa Biosystems) and a Bio-Rad CFX96 (Bio-Rad Laboratories). I amplified two replicated of 1 ng enriched, amplified DNA from each pool at all seven loci, and compared these results to two replicates of 1 ng unenriched, amplified DNA from each pool. Using the resulting data output, I calculated the average replicate crossing point (Cp) values for each pool at each amplicon, and then computed fold-enrichment values assuming an efficiency of 1.78 and using the formula 1.78abs(enriched Cp-unenriched Cp).

After verification, I used quantitative qPCR to measure the size-adjusted DNA concentration of each pool by creating serial dilutions (1:10^6^, and 1:20^6^) and performed qPCR library quantification, assuming an average library fragment length of 600 bp. Using the quantitative qPCR concentrations, I combined all pools into an equimolar final pool. The final pool was sent to the High Throughput Genomics Facility at the University of Utah. The pool was sequenced as a single lane on an Illumina HiSeq 2500 (125 cycle paired end sequencing v4).

*Processing and alignment: UCE dataset*

Demultiplexed data in the form of fastq files were downloaded via file transfer protocol from the sequencing center and were processed using the software package PHYLUCE v 1.5 [29, 30]. Raw reads were cleaned of adapter contamination and low-quality bases using the program *illumiprocessor,* which employs Trimmomatic [31] and is included in the PHYLUCE package. Cleaned reads were assembled *de novo* using the script *phyluce_assembly_assemblo_trinity*, which is a wrapper around the assembly program Trinity v 2013-02-25 [32]. Trinity has been demonstrated to create better assemblies with hymenopteran UCE data than other assembly programs [24]. I mapped the contigs from Trinity to UCE loci using the script *phyluce_assembly_match_contigs_to_probes*. Then, I used *phyluce_assembly_get_match_counts* and *phyluce_assembly_get_fastas_from_match_counts* to extract FASTA sequences from the matched contigs and concatenate them into one large data matrix, using the incomplete-matrix flag during these steps to allow matrices to have missing data. I aligned all loci individually using *phyluce_align_seqcap_align* with the *no-trim* and *incomplete-matrix* options*,* which runs the default version of MAFFT without trimming or excluding loci with incomplete matrices. Next, I used the L-INS-I algorithm in MAFFT to re-align the individual UCE loci. After alignment, I used ZORRO to estimate alignment confidence values for all loci, and trimmed all sites that fell below a threshold of five.

*Phylogenetic inference: UCE dataset*

To examine the effect of missing data on phylogenetic inference, I constructed a series of matrices from loci that contained a minimum of 25, 50, 75, 90, 95, 99 and 100 percent of all taxa (‘min25’, ‘min50’, ‘min75’, ‘min90’, ‘min95’, ‘min99’, ‘min100’ datasets). I then calculated summary statistics and estimated a phylogeny for each concatenated matrix with AMAS [33] and RAxML, respectively. For each RAxML tree inference, I used an unpartitioned matrix with 200 rapid bootstrap replicates, the GTR+G substitution model, and estimated the best tree annotated with bootstrap support values for each node. I wrote a custom shell script to calculate average bootstrap support for each tree. By comparing number of parsimony informative sites, proportion of parsimony informative sites, and average ML bootstrap support against percent missing data, I selected the minimum 90 percent taxa present matrix (‘min90’) and the 75% complete matrix (‘min75’) for further analyses (see Additional table [11] for summary statistics).

Because UCEs typically consist of a mixture of exons and non-coding DNA data, partitioning these data into coding vs. non-coding and by codon position is often not possible. Additionally, many phylogenetic search programs that are scalable to phylogenomic data only implement the GTR substitution model. To address the issue of substitution rate heterogeneity, I used the *kmeans* and *rcluster* clustering algorithms as implemented in the program PartitionFinder 2 [13, 34-36] to partition the ‘min90’ matrix into data subsets that share similarly evolving sites, limiting the model search to GTR+G. For the *kmeans* search, I used the complete concatenated dataset as input, and for *rcluster* I used the full dataset partitioned by UCE locus. Additionally, I used a script developed by Borowiec [37] to extract protein coding sequence from the ‘min75’ dataset, then trimmed and aligned the extracted sequence using the local version of the program TranslatorX [38] (‘coding’ dataset). I inspected each alignment by eye using Aliview [39], and discarded sequences that were mismatched and most likely contaminants. I then partitioned the protein-coding data from each locus by codon position and used the *rcluster* algorithm in PartitionFinder 2 to gather these into similarly evolving subsets. I used the best partitioning schemes found by PartitionFinder to estimate trees with RAxML, using the same settings as in the missing data analysis above for the missing data analyses. To investigate the properties of single UCE loci in preparation for downstream coalescent analyses, I estimated single UCE locus trees in RAxML using the same settings listed above for the ‘min90’ dataset.

For the BI search, I used the ‘min90’ dataset partitioned by the *kmeans* and *rcluster* algorithms, and the ‘coding’ dataset partitioned by *rcluster* as input. For each dataset, I used ExaBayes [40] to execute two independent runs, each with four coupled chains (one cold and three heated chains). I linked branch lengths across partitions and ran each analysis for one million generations. I assessed convergence among runs and run performance with the program Tracer, and used the *postProcParam,* *sdsf,* and *consense* utilities to summarize sampled parameters, inspect PSRF values, calculate average standard deviations of split frequencies, and constructed consensus trees using scripts included in the ExaBayes package after discarding the initial 25% of trees as burnin.

*Sensitivity analyses: UCE dataset*

To test the sensitivity of tree topology and node support to subsets of UCE data, I conducted an array of analyses to examine the effects of compositional heterogeneity (‘RCFV’ datasets), evolutionary rate (‘slow’ datasets), random samples of UCE loci (‘rand’ datasets), and gene-tree species-tree conflict (‘ASTRAL’ analysis).

Relative composition frequency variability (RCFV) is a measure of absolute deviation from the mean for each nucleotide in each locus [41]. To inspect the effect of compositional heterogeneity on phylogenetic inference, I used BaCoCa v.1.1.r to calculate RCFV scores on the ‘min90’ taxa alignment for each UCE locus and assembled matrices from UCE loci with the 33%, 66% and 90% lowest RCFV scores (‘rcfv33’, ‘rcfv66’, and ‘rcfv90’ datasets) and estimated ML trees in RAxML.

To account for the effect of evolutionary rate on tree inference, I used a python script written by Borowiec [42] to calculate the average branch length of each UCE locus tree estimated by RAxML. I made matrices of 33%, 66% and 90% slowest evolving loci (‘slow33’, ‘slow66’, and ‘slow90’ datasets) and estimated ML trees from these in RAxML.

To evaluate whether the topology and support values of the filtered datasets are a product of the parameters in question or reduced information content, I generated sets of 33%, 66% and 90% loci using the *random.sample* function in Python v2.7 (‘rand33_1’, ‘rand33_2’, ‘rand33_3’, ‘rand66’, and ‘rand90’ datasets), and estimated trees from these data matrices in RAxML. I calculated summary statistics for the data matrices above with AMAS (see Additional Table [5]).

To assess the potential influence of gene-tree conflict on species-tree inference I estimated the species tree using the summary coalescent method implemented in ASTRAL v4.10.12 [43]. Because gene tree estimation error can lead to inaccurate species tree estimation, I employed the weighted statistical binning pipeline developed by Bayzid *et al.* [44], using the single-locus trees as estimated in RAxML as input and setting the support threshold to 75. This grouped the loci into 150 bins, composed of 2 bins of 13 loci and 148 bins of 14 loci. Following binning, I concatenated these genes into supergenes and inferred supergene trees with 100 bootstrap replicates in RAxML. I then weighted the resulting ML trees by the number of loci that they were composed of and used these as input for ASTRAL, conducting the species tree analysis with 100 multi-locus bootstrap replicates.

*Constraint analyses*

Four ‘backbone’ nodes were poorly supported among the UCE analyses (labeled i-iv in Figure [2]) and each was subjected to constraint analyses. Because Bayesian analyses are computationally expensive on large datasets, I performed the stepping stone sampling method developed by Xie et al. [45] on the Sanger sequencing dataset, constraining the trees into two sets of competing hypotheses to calculate Bayes factors, in accordance with the recommendations of Bergsten *et al.* [42]. Specifically, in the first analysis I tested whether:

1. the *rugatulus* clade or the *obturator* clade is the sister group to [*obturator* clade + *salvini* clade + Palearctic clade];
2. the *obturator* clade is sister to the Palearctic clade or the *salvini* clade;
3. the *iris* subclade is sister to the *tricarinatus* group or the mmp11 group, or whether the tricarinatus group and mmp11 groups are sister;
4. *Gauromyrmex* and *Vombisidris* are sister, *Gauromyrmex* is sister to [*Vombisidris* + LGG + *Temnothorax*], or *Vombisidris* is sister to [*Gauromyrmex* + LGG + *Temnothorax*].

I ran these analyses in MrBayes 3.2.6 via CIPRES with two independent runs per analysis, setting the value of the alpha-shape parameter of the beta distribution to 0.4, number of chains to four, and sampling 25.5 million MCMC generations every 100^th^ generation for 50 steps between the posterior and the prior, and discarding the initial 500 thousand MCMC steps as burn-in. After initial runs, the marginal likelihoods of the topology tests within *Temnothorax* were all very close; I subsequently increased the number of runs for each test at nodes i-iii to four.

I reconciled the results of these two sets of analyses by constraining the backbone of the more species-rich Sanger sequencing dataset based on the results of the UCE analyses and stepping stone analyses, and re-estimated the phylogeny in BI and ML frameworks (see Figure [3] for the final constraints used). For BI, I used MrBayes with the settings for the full, unconstrained dataset analysis. For the ML analyses I used and a beta version of IQTREE and RAxML, with the settings used above in the unconstrained analyses, and using the ‘–g’ option for both programs, which uses a multifurcating tree as input for the constrained tree search.

*Morphology*

To properly place the fossil taxon *Temnothorax praecreolus* in the phylogeny, I recorded characters and took measurements from this fossil, as well as all *Temnothorax* worker specimens used in the phylogeny. The final dataset comprised twenty-nine characters, which were either inherently discrete, discretized continuous characters, or discretized indices (see Additional file [4] and Additional table [6]). Because the morphological dataset is small compared to the molecular dataset, I expected the influence of this addition to be minimal on topology. However, to gauge the influence of the morphological data on the phylogeny, I ran an analysis of the morphology dataset in MrBayes, both with and without the molecular data (see Additional file [9]). I used the standard discrete morphology model, with the “coding” parameter set to “variable”, which implements the Lewis Mkv model. Both analyses use four chains and two independent runs, sampling every 10,000 generations, and were run for 50 M generations.

*Divergence time analysis*

Divergence dates in *Temnothorax* were inferred using a combination of node- and tip-dating approaches in the program BEAST 2.3.0 [47]. I used morphological data to place *Temnothorax praecreolus*, but due to the uncertainty of the placement of Baltic amber specimens (Calibration 3 below), I compared two analyses, using these fossils to calibrate: (a) the node subtending the CF (b) the node subtending [LGG + Temnothorax]. In the following, I provide details on the node calibrations used: the node and clade that it subtends are given first, followed by the type of prior distribution, then a series of three numbers representing the upper 95% bound, the median, and the lower 95% bound in units of millions of years ago (Ma). I used normal priors on all secondary node calibrations because these distributions best fit the data output from the original studies that they are based on. The actual values used as input for the BEAST distribution model

parameters are given in parentheses.

1. Root node calibration. Myrmicinae. Normal prior, 118-96.6-75.5 (offset 96.6, SD 12.8). This is a secondary calibration point based on the age of the crown-group Myrmicinae inferred from three studies: the mean age of was calculated by first finding the mean of the dates reported from two calibration approaches in Moreau *et al.* [48], then finding the mean of the set of ages presented in Brady *et al.* [49], and finally finding the mean of these two studies and Ward *et al.* [1]. The standard deviation was set to encompass the upper and lower ages across these three studies.
2. Calibration 1. [Solenopsidini + Attini + Crematogastrini]. Normal prior, 87-79-71 (offset 79, SD 4.84). This is a secondary calibration point based on the ages reported in Ward *et al.* [1].
3. Calibration 2. Crematogastrini. Normal prior, 78-71-64 (offset 71, SD 4.23). This is another secondary calibration point based on the ages reported in Ward *et al.* [1].
4. Calibration 3. ‘*Temnothorax*’ spp. in Baltic amber [50]. Lognormal prior, 73-52-45.2 (mean 2.3, SD 0.69, offset 42).

I used the combined concatenated Sanger molecular and morphology dataset as input, with the molecular data partitioned according to the best scheme found by PartitionFinder. For the molecular data partitions, I used the ‘RB’ reversible-jump MCMC method to sample across all substitution models for each partition, setting the among-site rate variation to the models suggested by PartitionFinder. For the morphology partition, I used the Lewis Mkv model. I chose an uncorrelated lognormal clock model [51] and a Fossilized Birth-Death speciation process [52] for the tree prior, with both priors linked across partitions. In the tree prior, I left the diversification rate, turnover and sampling proportion parameters at their default settings, changing the sampling proportion prior to a beta distribution with the shape parameters alpha and beta equal to 2. Additionally, I changed the proportion of extant species sampled (rho) to 0.2, based on the assumption that my 103 ingroup taxon dataset represents 1/5 of the estimated 500 extant species of *Temnothorax*. I changed the mean prior distribution on the clock model to exponential, with a mean of 10. I constrained the tree topologically based on the results of the UCE and stepping stone analyses (see Figure [3] for constraints). Two identical independent runs with were executed for the analysis. To check that the data was influencing the priors, I performed an additional null run, in which all parameters were identical to the previous two runs, but with the data removed. Each analysis was set to run for 100 million generations, sampling every 10 thousand generations.

Time series plots and ESS values were visualized with Tracer to assess the appropriate proportion of burn-in, as well to check that the runs converged and were mixing adequately. The analyses with data included produced high ESS values (greater than 200) in both runs for all parameters, excluding some of the rj-MCMC model sampling parameters. I combined the resulting tree files with the program LogCombiner v1.8.0, resampling at a frequency of 20,000 generations, and generated a consensus chronogram with TreeAnnotator v1.8.0 with the initial 2500 of the 10,000 resulting trees discarded as burn-in.

*Biogeographic analysis*

I used the consensus chronogram from the BEAST analysis as input for the likelihood-based R package BioGeoBEARS [53], pruning all outgroup taxa from the tree except for the CF. I used a biogeographical classification scheme following previous studies (*e.g.* [1]), but discretized the Caribbean islands, including the Bahamas, into a separate biogeographical unit given the high diversity of *Temnothorax* species on these islands, and their historical isolation from the mainland [54] (see the key in Figure [4] for a summary of the areas used). Because no extant species in the phylogeny inhabits more than two biogeographical areas, I limited the maximum ancestral species range to two units. Because the ancestor of the CF is inferred to have arisen between 60 and 50 Ma, relatively little tectonic drift with respect to the current positions of the continents has taken place in the evolutionary history of this group; therefore, I used a single time slice for this analysis. I imposed matrices of dispersal constraints, dispersal multipliers, area adjacencies, and allowed ancestral ranges on the analysis (see script and input files on Dryad, available upon acceptance). I ran the program over all six models included in the example script (DEC, DEC+J, DIVALIKE, DIVALIKE+J, BAYAREALIKE, BAYAREALIKE+J), and compared the models via AICc score. Because the models offered alternate reconstructions, I averaged the results across all six models using the AICc weights in the output file.

The genera *Harpagoxenus* and *Formicoxenus*, in the LGG, are Holarctic in distribution, but my taxon sampling was limited to a Palearctic *Harpagoxenus* (*H. sublaevis*) and a Nearctic *Formicoxenus* (*F. diversipilosus*), which may introduce bias into the biogeographic reconstruction. Additionally, Vombisidris has a Indomalayan-Australasian distribution, but my dataset contained a single Australasian species. To test the sensitivity of the empirical reconstruction to incomplete outgroup sampling, I recoded the tip states for these genera in all 27 possible permutations of bioregion combinations:

(*Vombisidris*(Aus, Ind, Aus-Ind))

*(*Harpagoxenus*(Nea, Pal, Nea-Pal))

*(*Formicoxenus*(Nea, Pal, Nea-Pal))

= 27 alternate reconstruction scenarios

For each reconstruction scenario, I averaged over all six models, as in the empirical example above. Finally, I averaged over all 27 model-averaged reconstructions, weighting each equally, to account for reconstruction uncertainty due to incomplete outgroup sampling.

*Ancestral state reconstruction*

To reconstruct the historical shifts in nesting habitat and parasitism in *Temnothorax*, as well as the potential effects of these characters on diversification rates, I estimated the ancestral states of these two traits using the hidden state speciation and extinction (HiSSE) model [55], which improves upon the binary state speciation and extinction BiSSE model by considering the possibility of unobserved traits affecting trait associated diversification rates. I gathered nesting habitat and behavioral data from the literature, collection information, and personal observation, and coded all extant taxa according to whether they are:

1. arboricolous, nesting exclusively in vegetable matter, living or dead, above the soil and leaf litter layer, or
2. terricolous, nesting in variety of habitats, but always including the soil, leaf litter layer, and/or fallen, rotten logs.

In the parasitism analysis, species were coded as:

1. free living, if nests are founded independently, or
2. social parasites, if new nests are founded exclusively in the nests of other ants; I include the xenobiotic genus *Formicoxenus* in this category.

Because the taxon sampling in this phylogeny is incomplete, I used global sampling fractions for each analysis, modeled as the proportion of species in each state that are included in the phylogeny.

For the arboreality dataset, I searched among 59 models (see Additional table [8]). Several of these are BiSSE-like models, which vary by having separate transition rates for each state (‘ARD’ models), equal transition rates (‘ER’ models), equal extinction fractions, and by disallowing transitions from arboreality to terrestriality. Next, I constructed variations on the full HiSSE model, manipulating the diversification parameters among models. Because transition rates are generally difficult to accurately estimate in the full model [55], I limited my search to a subset of the models in which transition rates are equal. Following the advice of Beaulieu & O’Meara [55], I also removed models in which dual transitions between the observed trait and the hidden trait (e.g. q0a <-> q1b and q0b <-> q1a) are allowed. Also, because HiSSE has difficulty estimating extinction fraction, I paired each model with multiple state-associated extinction fractions with an identical model that held extinction fractions equal for each state. I then added models that disallowed transitions from arboreality to terrestriality and, based on preliminary results, a set of models without a hidden state associated with arboreality. I specified several models that disallowed the combination of transitions from aboreality to terrestriality and the presence of a hidden state associated with arboreality. Finally, I included variations on null models, which operate under the assumption that diversification rates are independent of character states (‘CID’ models). HiSSE initially returned a surprising result, showing that a hidden state associated with arboreality was responsible for a two-fold decrease in net diversification rate. I inspected reconstructions of the states on the tree, and found a single transition to the hidden state at the root of the tree, subtending the long-branched outgroups. After trimming *Gauromyrmex* and *Vombisidris* from the dataset, all traces of character dependent diversification rates disappeared.

For the parasitism dataset, I constructed 41 models (see Additional file [9]) using most of the models mentioned above, with a subset disallowing transitions from parasitism to free living. Unlike the arboreality model search, I didn’t include HiSSE models that disallowed hidden states. For both datasets I summarized all reconstructions, accounting for model uncertainty, by averaging over all models after weighting each model by its AIC score (see Figure [5] and Additional file [10]).

**References**

1. Ward PS, Brady SG, Fisher BL, Schultz TR. The evolution of myrmicine ants: phylogeny and biogeography of a hyperdiverse ant clade (Hymenoptera: Formicidae). Syst Ent. 2015;40:61-81.
2. Ward PS, Downie DA. The ant subfamily Pseudomyrmecinae (Hymenoptera: Formicidae): phylogeny and evolution of big‐eyed arboreal ants. Syst Ent. 2005;30:310-35.
3. Weisrock DW, Smith SD, Chan LM, Biebouw K, Kappeler PM, Yoder AD. Concatenation and concordance in the reconstruction of mouse lemur phylogeny: an empirical demonstration of the effect of allele sampling in phylogenetics. Mol Biol Evol. 2012;29:1615-30.
4. Katoh K, Standley DM. MAFFT multiple sequence alignment software version 7: improvements in performance and usability. Mol Biol Evol. 2013;30:772-780.
5. Nuin P, Wang Z, Elisabeth R. The accuracy of several multiple sequence alignment programs for proteins. BMC Bioinformatics. 2006;7:471.
6. Golubchik T, Wise M, Easteal S, Jermiin L. Mind the gaps: evidence of bias in estimates of multiple sequence alignments. Mol Biol Evol. 2007;24:2433-42.
7. Pais FSM, de Cássia Ruy P, Oliveira G, Coimbra RS. Assessing the efficiency of multiple sequence alignment programs. Algorithms Mol Biol. 2014;9:1.
8. Maddison WP, Maddison DR. Mesquite: a modular system for evolutionary analysis. Version 3.10. 2016. http://mesquiteproject.org.
9. Wu M, Chatterji S, Eisen JA. Accounting for alignment uncertainty in phylogenomics. PLoS ONE. 2012;7:e30288.
10. Fernández R, Edgecombe GD, Giribet G. Exploring phylogenetic relationships within Myriapoda and the effects of matrix composition and occupancy on phylogenomic reconstruction. Syst Biol. 2016:syw041.
11. Kück P, Struck TH. BaCoCa–A heuristic software tool for the parallel assessment of sequence biases in hundreds of gene and taxon partitions. Mol Phylogenet Evol. 2014;70:94-8.
12. Buckley TR, Simon C, Chambers GK. Exploring among-site rate variation models in a maximum likelihood framework using empirical data: effects of model assumptions on estimates of topology, branch lengths, and bootstrap support. Syst Biol. 2001;50:67-86.
13. Lanfear R, Calcott B, Ho SYW, Guindon S. PartitionFinder: combined selection of partitioning schemes and substitution models for phylogenetic analyses. Mol Biol Evol. 2012;29:1695-701.
14. Sullivan J, Swofford DL, Naylor GJP. The effect of taxon sampling on estimating rate-heterogeneity parameters of maximum-likelihood models. Mol Biol Evol. 1999;16:1347-56.
15. Sullivan J, Swofford DL. Should we use model-based methods for phylogenetic inference when we know assumptions about among-site rate variation and nucleotide substitution pattern are violated? Syst Biol. 2001;50:723-9.
16. Yang Z. Computational molecular evolution. Oxford University Press, Oxford; 2006.
17. Stamatakis A. RAxML-VI-HPC: maximum likelihood-based phylogenetic analyses with thousands of taxa and mixed models, Bioinformatics. 2006;22:2688-90.
18. Nguyen LT, Schmidt HA, von Haeseler A, Minh BQ. Iq-tree: A fast and effective stochastic algorithm for estimating maximum-likelihood phylogenies. Mol Biol Evol. 2015;32:268-74.
19. Ronquist F, Teslenko M, van der Mark P. MrBayes 3.2: efficient Bayesian phylogenetic inference and model choice across a large model space. Syst Biol. 2012;61:539-42.
20. Miller MA, Pfeiffer W, Schwartz T. 2010. Creating the CIPRES Science Gateway for inference of large phylogenetic trees in Proceedings of the Gateway Computing Environments Workshop (GCE) New Orleans, LA; 14 Nov. 2010. pp. 1-8.
21. Rambaut A, Suchard MA, Xie D, Drummond AJ. Tracer v1.6. 2014. http://tree.bio.ed.ac.uk/software/tracer. Accessed 1 April 2014.
22. Ané C, Larget B, Baum DA, Smith SD, Rokas A. Bayesian estimation of concordance among gene trees. Mol Biol Evol. 2007;24:412-26.
23. Larget B, Kotha SK, Dewey CN, Ané C. BUCKy: Gene tree/species tree reconciliation with the Bayesian concordance analysis. Bioinformatics. 2010;26:2910-1.
24. Faircloth BC, Branstetter MG, White ND, Brady SG. Target enrichment of ultraconserved elements from arthropods provides a genomic perspective on relationships among Hymenoptera. Mol Ecol Res. 2015;15:489–501.
25. Branstetter MG, Longino JT, Ward PS, Faircloth BC. Enriching the ant tree of life: enhanced UCE bait set for genome‐scale phylogenetics of ants and other Hymenoptera. Methods in Ecology and Evolution. 2017; 8, 768-776.
26. Rohland N, Reich D: Cost-effective, high-throughput DNA sequencing libraries for multiplexed target capture. Genome Res 2012, 22:939–946.
27. Fisher S, Barry A, Abreu J, Minie B, Nolan J, Delorey TM, Young G, Fennell TJ, Allen A, Ambrogio L: A scalable, fully automated process for construction of sequenceready human exome targeted capture libraries. Genome Biol 2011, 12:R1.
28. Branstetter MG, Longino JT, Ward PS, Faircloth BC. Enriching the ant tree of life: enhanced UCE bait set for genome‐scale phylogenetics of ants and other Hymenoptera. Methods Ecol Evol. 2017;8:768-76.
29. Faircloth BC, McCormack JE, Crawford NG, Harvey MG, Brumfield RT, Glenn TC. 2012. Ultraconserved elements anchor thousands of genetic markers spanning multiple evolutionary timescales. Syst Biol. 2012;61:1-10.
30. Faircloth BC. PHYLUCE is a software package for the analysis of conserved genomic loci. Bioinformatics: Advance Access 2015:1-3.
31. Bolger AM, Lohse M, Usadel B. Trimmomatic: a flexible trimmer for Illumina sequence data. Bioinformatics. 2014 Apr 1;30(15):2114-20.
32. Grabherr MG, Haas BJ, Yassour M, Levin JZ, Thompson DA, Amit I, Adiconis X, Fan L, Raychowdhury R, Zeng Q, Chen Z, Mauceli E, Hacohen N, Gnirke A, Rhind N, di Palma F, Birren BW, Nusbaum C, Lindblad-Toh K, Friedman N, Regev A. Full-length transcriptome assembly from RNA-Seq data without a reference genome. Nature Biotechnol. 2011;29:644-52.
33. Borowiec ML. AMAS: a fast tool for alignment manipulation and computing of summary statistics. PeerJ. 2016;4:e1660.
34. Lanfear R, Frandsen PB, Wright AM, Senfeld T, Calcott B.. PartitionFinder 2: new methods for selecting partitioned models of evolution for molecular and morphological phylogenetic analyses. Mol Biol Evol. 2016:msw260.
35. Frandsen PB, Calcott B, Mayer C, Lanfear R. 2015. Automatic selection of partitioning schemes for phylogenetic analyses using iterative k-means clustering of site rates. BMC Evol Biol. 2015;15:13.
36. Guindon S, Dufayard JF, Lefort V, Anisimova M, Hordijk W, Gascuel O. New Algorithms and Methods to Estimate Maximum-Likelihood Phylogenies: Assessing the Performance of PhyML 3.0. Syst Biol. 2010;59:307-21.
37. Borowiec ML. Convergent evolution of the army ant syndrome and congruence in big-data phylogenetics. bioRxiv. 2017:134064.
38. Abascal F, Zardoya R, Telford MJ. TranslatorX: multiple alignment of nucleotide sequences guided by amino acid translations. Nucleic Acids Res. 2010;38:7-13.
39. Larsson A. AliView: a fast and lightweight alignment viewer and editor for large data sets. Bioinformatics. 2014;30:3276-8.
40. Aberer AJ, Kobert K, Stamatakis A. ExaBayes: massively parallel Bayesian tree inference for the whole-genome era. Mol Biol Evol. 2014;31:2553-6.
41. Zhong M, Hansen B, Nesnidal MP, Golombek A, Halanych K, Struck TH. Detecting the symplesiomorphy trap: A multigene phylogenetic analysis for terebelliform annelids. BMC Evol Biol. 2011;11:369.
42. Borowiec ML, Lee EK, Chiu JC, Plachetzki DC. Extracting phylogenetic signal and accounting for bias in whole-genome data sets supports the Ctenophora as sister to remaining Metazoa. BMC Genomics. 2015;16:987.
43. Mirarab S, Warnow T. ASTRAL-II: coalescent-based species tree estimation with many hundreds of taxa and thousands of genes. Bioinformatics. 2015;31:i44-i52.
44. Bayzid MS, Mirarab S, Boussau B, Warnow T. Weighted statistical binning: enabling statistically consistent genome-scale phylogenetic analyses. PLoS ONE. 2015;10:e0129183.
45. Xie W, Lewis PO, Fan Y, Kuo L, Chen MH. Improving marginal likelihood estimation for Bayesian phylogenetic model selection. Syst Biol. 2011;60:150-60.
46. Bergsten J, Nilsson AN., Ronquist F. Bayesian tests of topology hypotheses with an example from diving beetles. Syst Biol. 2013;62:660-73.
47. Bouckaert R, Heled J, Kühnert D, Vaughan T, Wu C-H, Xie D, Suchard MA, Rambaut A, Drummond AJ. BEAST 2: A Software Platform for Bayesian Evolutionary Analysis. PLoS Comput Biol. 2014;10:e1003537.
48. Moreau CS, Bell CD, Vila R, Archibald SB, Pierce NE. Phylogeny of the ants: diversification in the age of angiosperms. Science. 2006;312:101-4.
49. Brady SG, Schultz TR, Fisher BL, Ward PS. Evaluating alternative hypotheses for the early evolution and diversification of ants. Proc Natl Acad Sci. 2006;103:18172-7.
50. Dlussky GM. Genera of ants (Hymenoptera: Formicidae) from Baltic amber. Paleonotl J. 1997;31:616-27.
51. Drummond AJ, Ho SYW, Phillips MJ, Rambaut A. 2006. Relaxed phylogenetics and dating with confidence. PLoS Biol. 2006;4:e88.
52. Heath TA, Huelsenbeck JP, Stadler T. The fossilized birth–death process for coherent calibration of divergence-time estimates. Proc Natl Acad Sci. 2014;111:e2957-66.
53. Matzke N. BioGeoBEARS: BioGeography with Bayesian (and likelihood) evolutionary analysis in R scripts. University of California, Berkeley, Berkeley, CA; 2013.
54. Iturralde-Vinent MA. A brief account of the evolution of the Caribbean seaway: Jurassic to present. In Prothero D, Ivany L, Nesbitt E, editors. From greenhouse to icehouse: The marine Eocene–Oligocene transition: New York, NY: Colombia University Press; 2003. pp. 386–396.
55. Beaulieu, J.M., and B.C. O'Meara. (2016). Detecting hidden diversification shifts in models of trait-dependent speciation and extinction. Syst Biol. 65:583-601.
