# Supplementary material for: Insights into the evolution, biogeography and natural history of the acorn ants, genus Temnothorax Mayr (hymenoptera: Formicidae)
Source: BMC Evol Biol. 2017 Dec 13;17:250. doi: 10.1186/s12862-017-1095-8 (PMC5729518; doi:10.1186/s12862-017-1095-8)
Supplement: Supplementary file 7 — Trees inferred from the full Sanger sequencing dataset and data subsetting experiments. (PDF 437 kb) [file 12862_2017_1095_MOESM7_ESM.pdf]

Figure A: BI

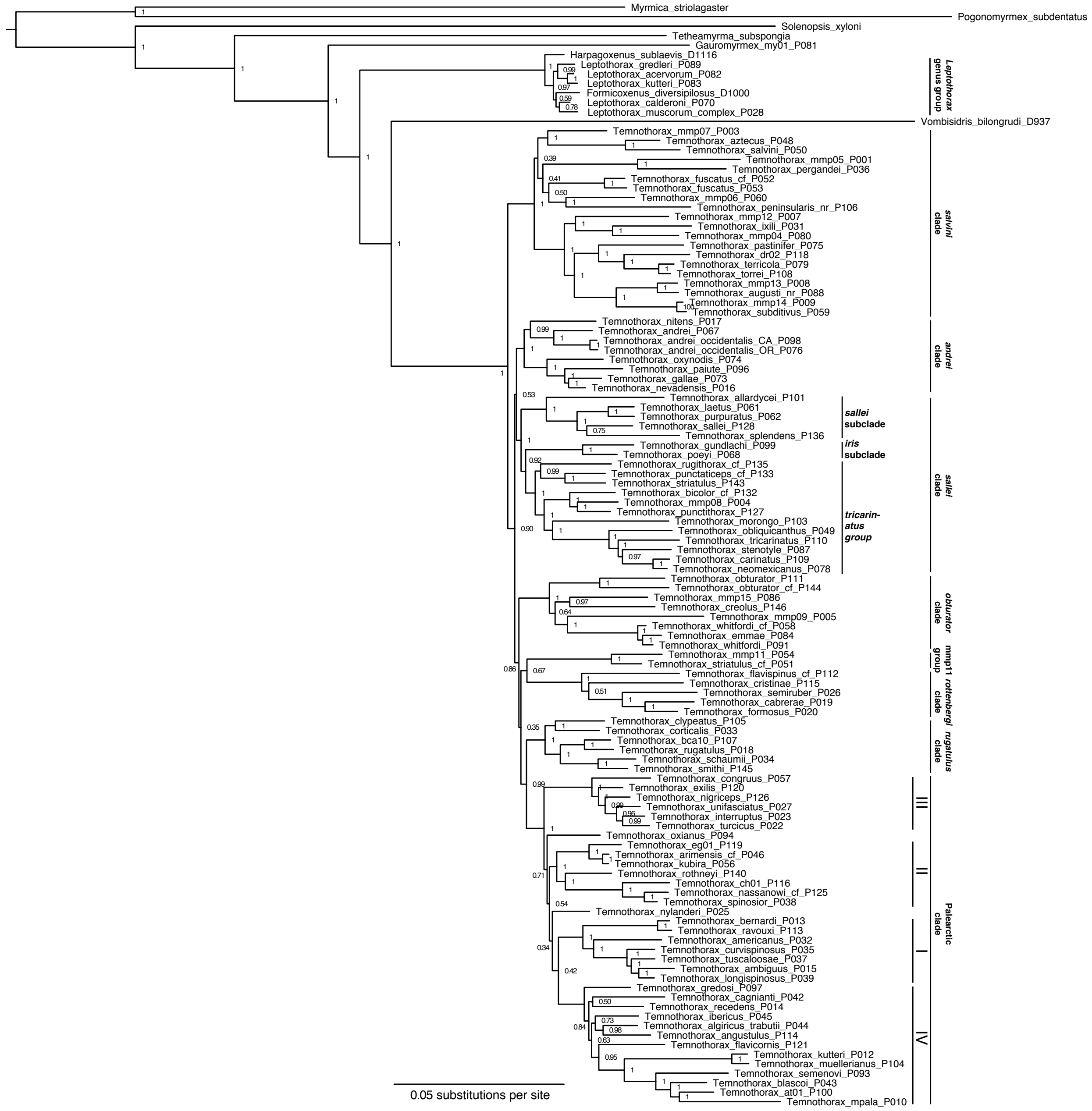

Myrmica_striolagaster
Pogonomyrmex_subdentatus
Solenopsis_xyloni
Tetheamyrma_subspongia
Gauromyrmex_my01_P081
Harpagoxenus_sublaevis_D1116
Leptothorax_gredleri_P089
Leptothorax_acervorum_P082
Leptothorax_kutteri_P083
Formicoxenus_diversipilosus_D1000
Leptothorax_calderoni_P070
Leptothorax_muscorum_complex_P028
Leptothorax genus group
Vombisidris_bilongrudi_D937
Temnothorax_mmp07_P003
Temnothorax_aztecus_P048
Temnothorax_salvini_P050
Temnothorax_mmp05_P001
Temnothorax_pergandei_P036
Temnothorax_fuscatus_cf_P052
Temnothorax_fuscatus_P053
Temnothorax_mmp06_P060
Temnothorax_peninsularis_nr_P106
Temnothorax_mmp12_P007
Temnothorax_ixili_P031
Temnothorax_mmp04_P080
Temnothorax_pastinifer_P075
Temnothorax_dr02_P118
Temnothorax_terricola_P079
Temnothorax_torrei_P108
Temnothorax_mmp13_P008
Temnothorax_augusti_nr_P088
Temnothorax_mmp14_P009
Temnothorax_subditivus_P059
salvini clade
Temnothorax_nitens_P017
Temnothorax_andrei_P067
Temnothorax_andrei_occidentalis_CA_P098
Temnothorax_andrei_occidentalis_OR_P076
Temnothorax_oxynodis_P074
Temnothorax_paiute_P096
Temnothorax_gallae_P073
Temnothorax_nevadensis_P016
andrei clade
Temnothorax_allardycei_P101
Temnothorax_laetus_P061
Temnothorax_purpuratus_P062
Temnothorax_sallei_P128
Temnothorax_splendens_P136
sallei subclade
Temnothorax_gundlachi_P099
Temnothorax_poeyi_P068
iris subclade
Temnothorax_rugithorax_cf_P135
Temnothorax_punctaticeps_cf_P133
Temnothorax_striatulus_P143
Temnothorax_bicolor_cf_P132
Temnothorax_mmp08_P004
Temnothorax_punctithorax_P127
Temnothorax_morongo_P103
Temnothorax_obliquicanthus_P049
Temnothorax_tricarinatus_P110
Temnothorax_stenotyle_P087
Temnothorax_carinatus_P109
Temnothorax_neomexicanus_P078
tricarin-atus group
sallei clade
Temnothorax_obturator_P111
Temnothorax_obturator_cf_P144
Temnothorax_mmp15_P086
Temnothorax_creolus_P146
Temnothorax_mmp09_P005
Temnothorax_whitfordi_cf_P058
Temnothorax_emmae_P084
Temnothorax_whitfordi_P091
obturator clade
Temnothorax_mmp11_P054
Temnothorax_striatulus_cf_P051
mmp11 group
Temnothorax_flavispinus_cf_P112
Temnothorax_cristinae_P115
Temnothorax_semiruber_P026
Temnothorax_cabrerae_P019
Temnothorax_formosus_P020
rottenbergi clade
Temnothorax_clypeatus_P105
Temnothorax_corticalis_P033
Temnothorax_bca10_P107
Temnothorax_rugatulus_P018
Temnothorax_schaumii_P034
Temnothorax_smithi_P145
rugatulus clade
Temnothorax_congruus_P057
Temnothorax_exilis_P120
Temnothorax_nigriceps_P126
Temnothorax_unifasciatus_P027
Temnothorax_interruptus_P023
Temnothorax_turcicus_P022
III
Temnothorax_oxianus_P094
Temnothorax_eg01_P119
Temnothorax_arimensis_cf_P046
Temnothorax_kubira_P056
Temnothorax_rothneyi_P140
Temnothorax_ch01_P116
Temnothorax_nassanowi_cf_P125
Temnothorax_spinosior_P038
II
Temnothorax_nylanderi_P025
Temnothorax_bernardi_P013
Temnothorax_ravouxi_P113
Temnothorax_americanus_P032
Temnothorax_curvispinosus_P035
Temnothorax_tuscaloosae_P037
Temnothorax_ambiguus_P015
Temnothorax_longispinosus_P039
I
Temnothorax_gredosi_P097
Temnothorax_cagnianti_P042
Temnothorax_recedens_P014
Temnothorax_ibericus_P045
Temnothorax_algiricus_trabutii_P044
Temnothorax_angustulus_P114
Temnothorax_flavicornis_P121
Temnothorax_kutteri_P012
Temnothorax_muellerianus_P104
Temnothorax_semenovi_P093
Temnothorax_blascoi_P043
Temnothorax_at01_P100
Temnothorax_mpala_P010
IV
Palearctic clade
0.05 substitutions per site

Figure B: ML, full dataset (IQTREE)

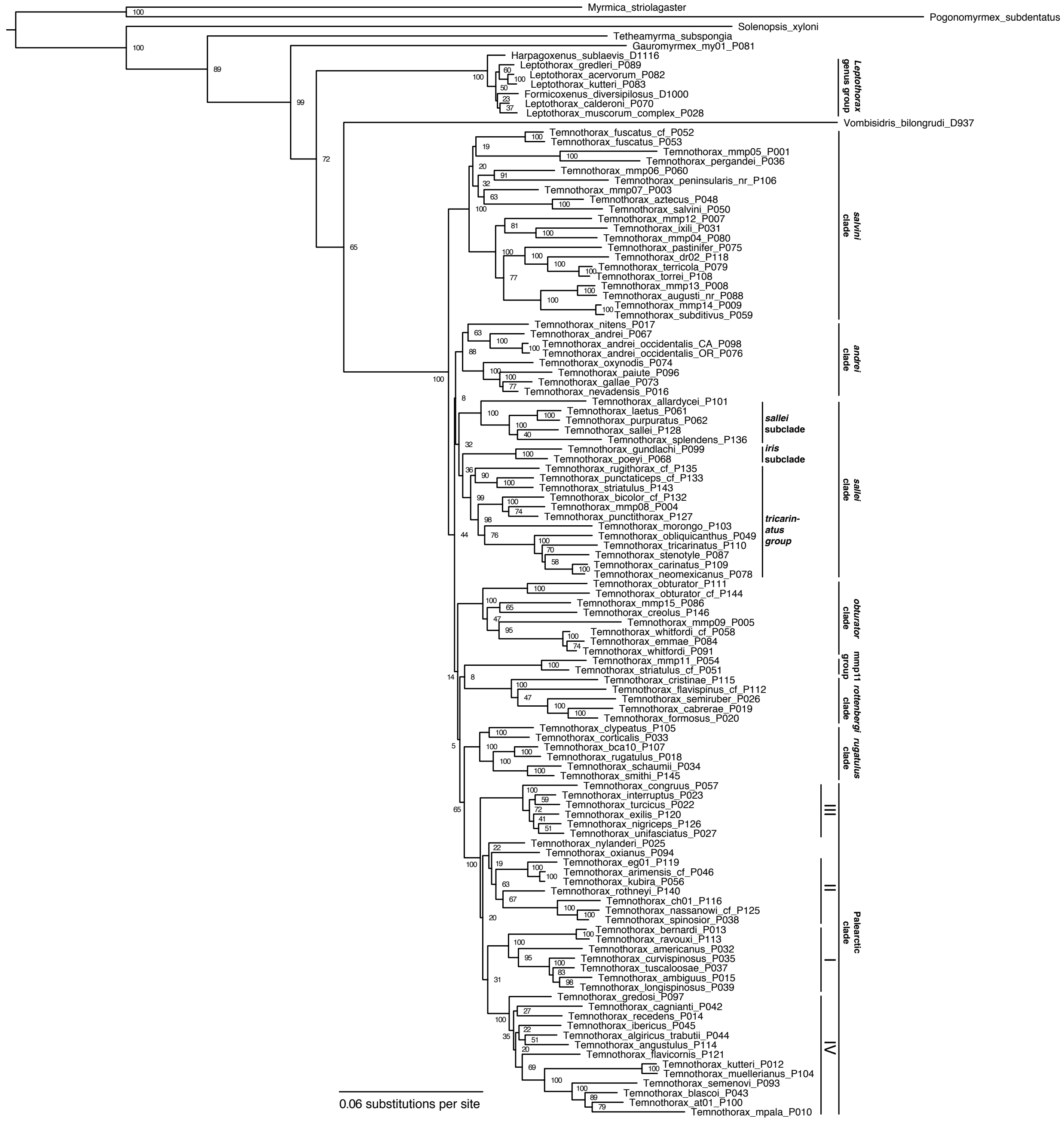

Figure C: ML, full dataset (RAxML)

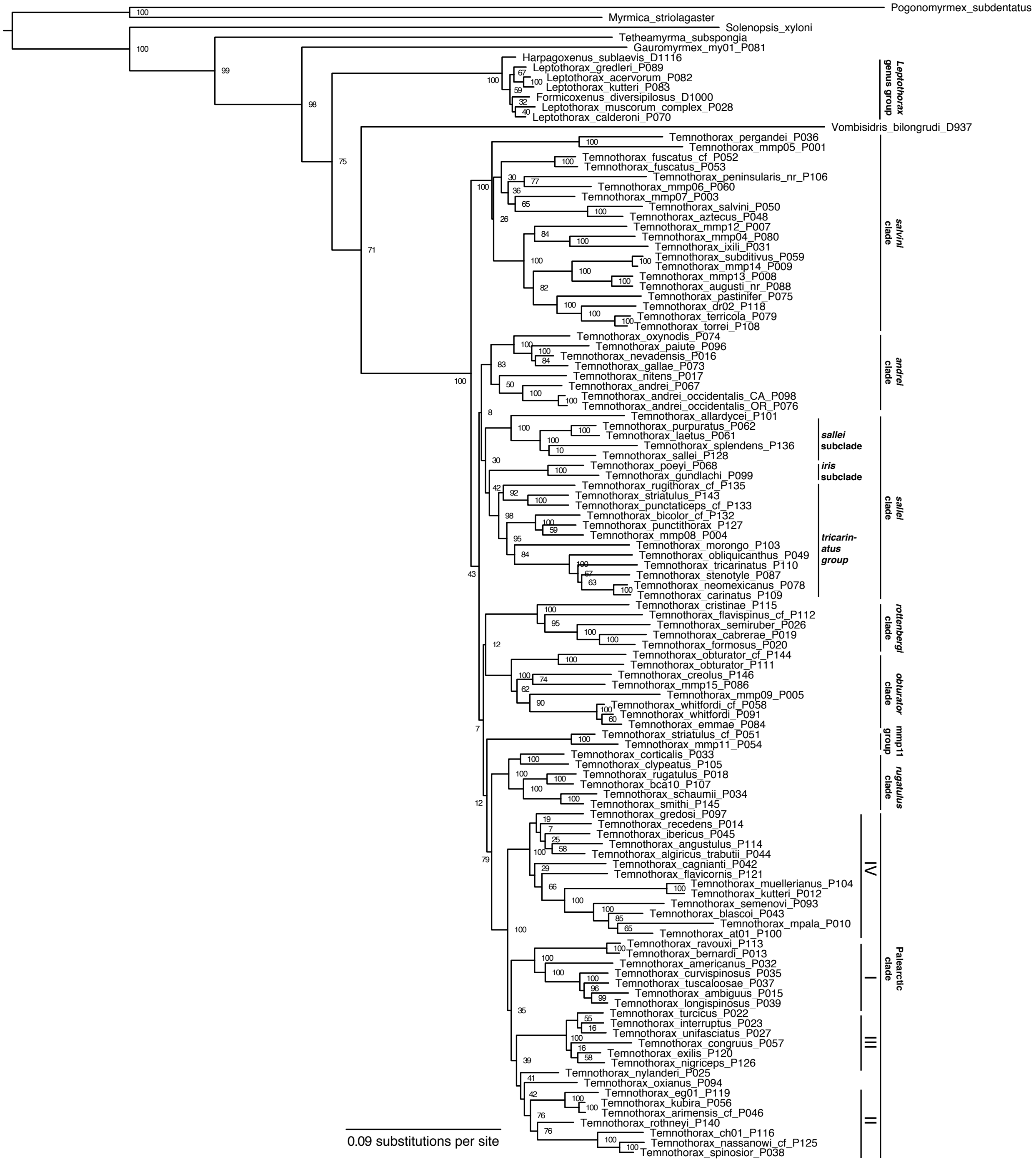

Figure D: ML, introns removed (RAxML)

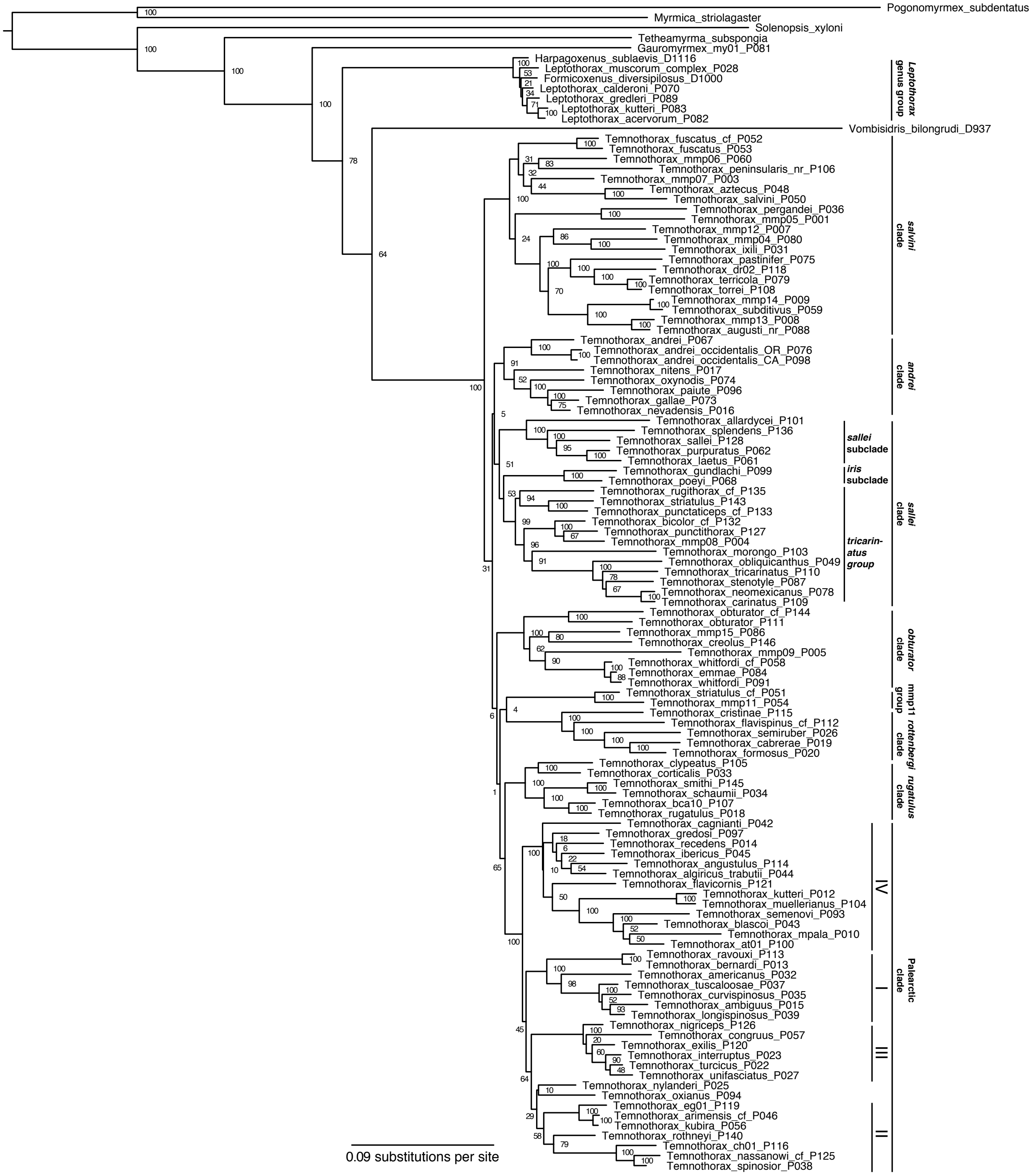

Figure E: ML, COI + COII removed (RAxML)

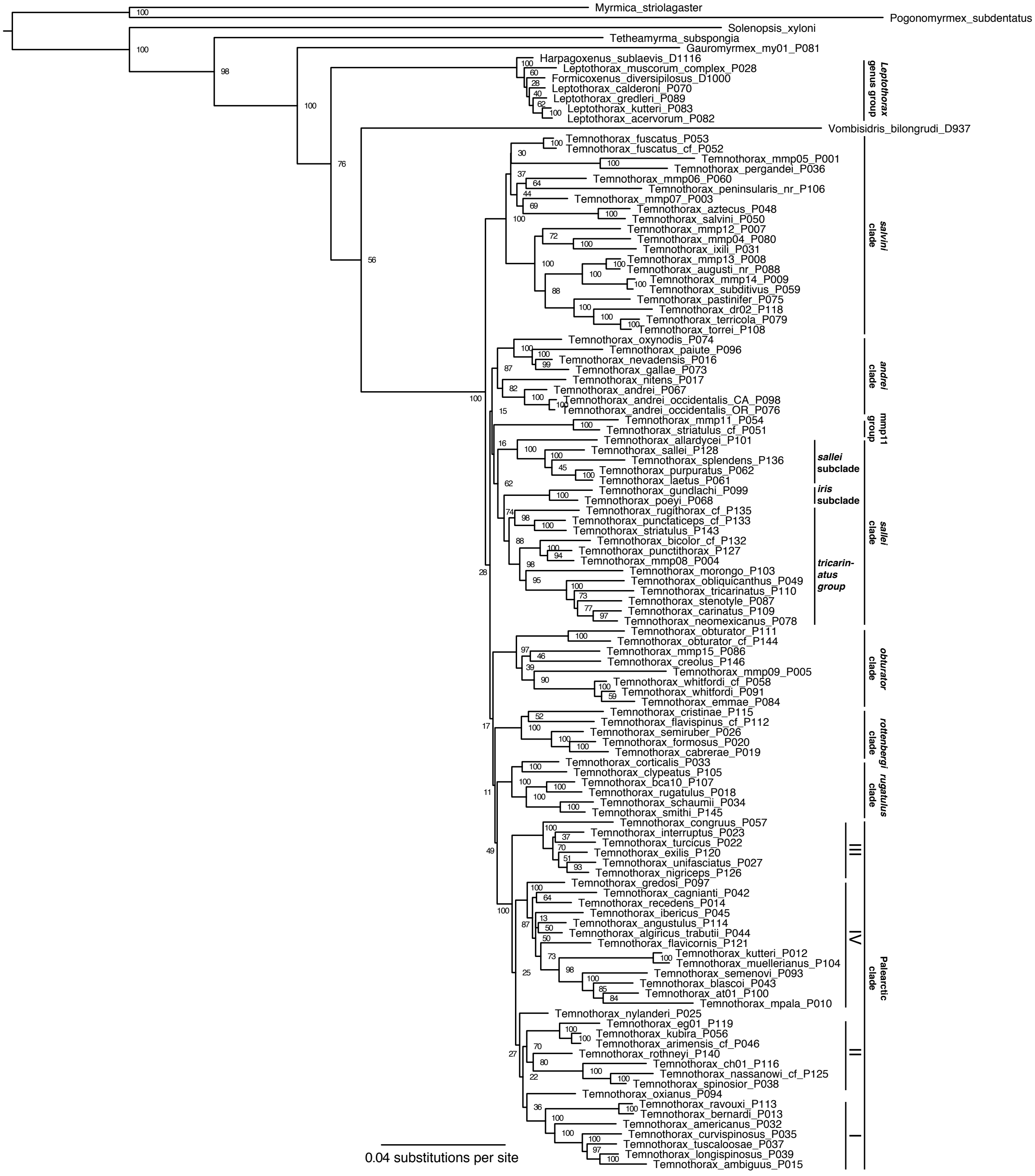

Figure F: ML, COI + COII and introns removed (RAxML)

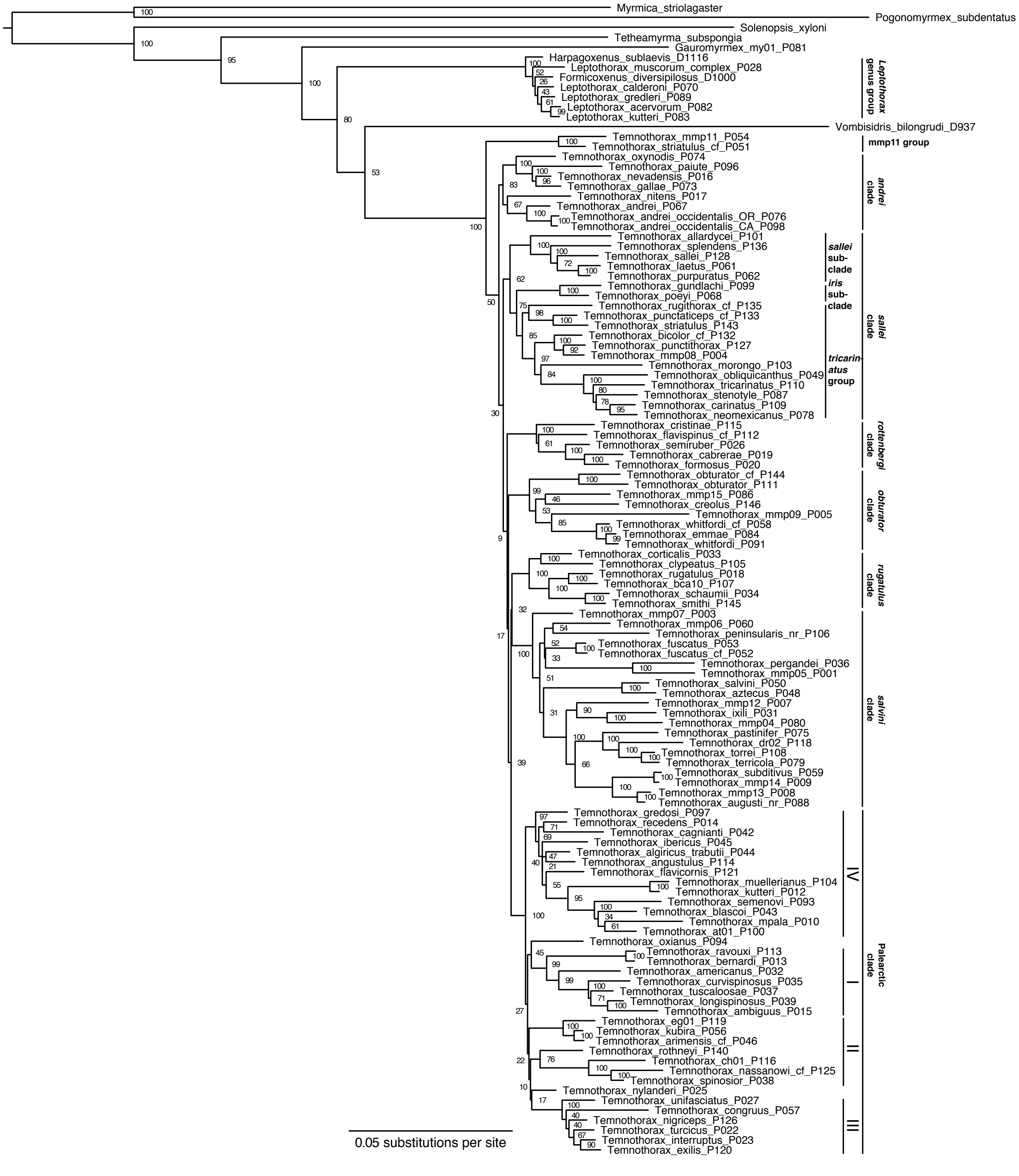

Figure G: gene concordance

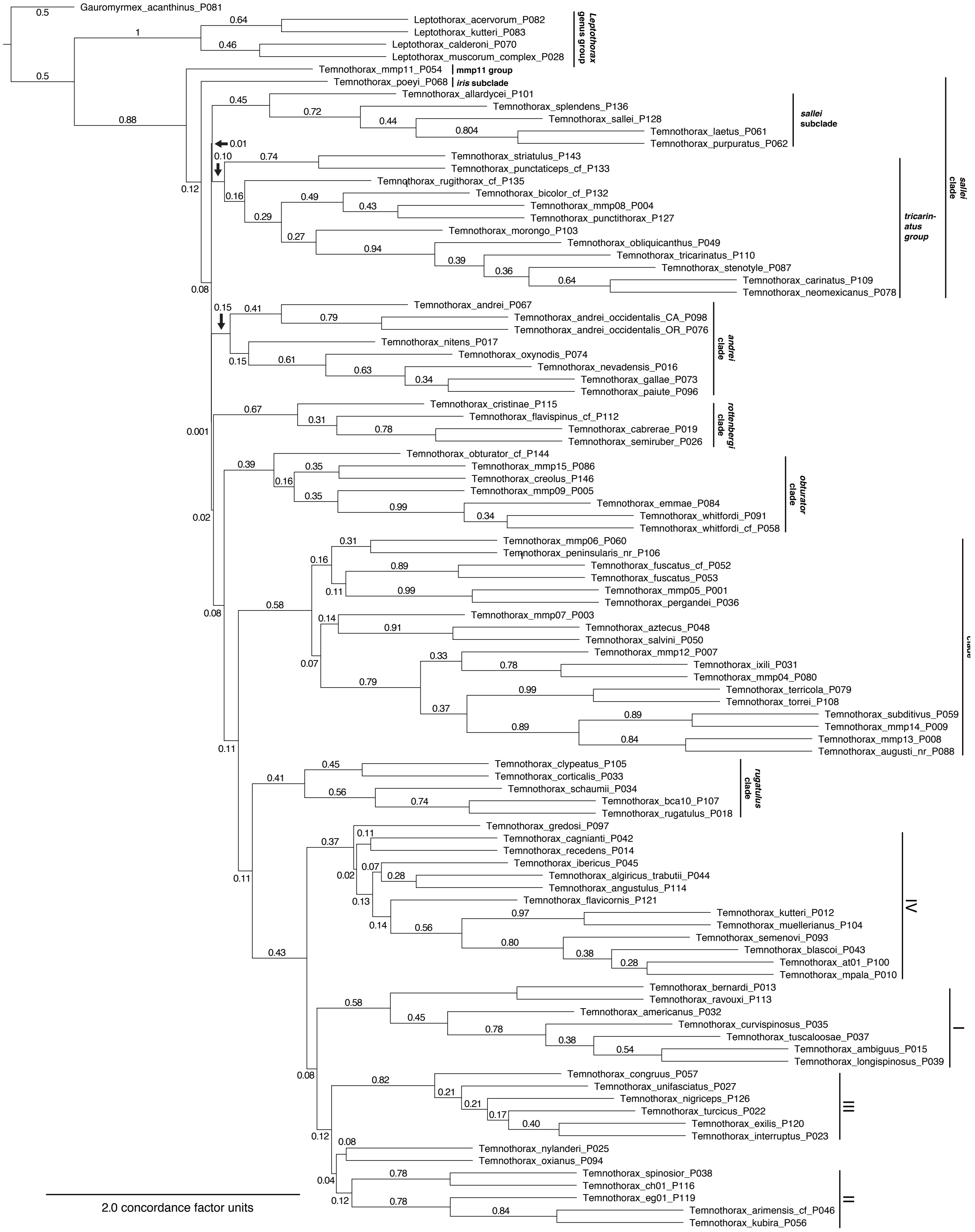

Figure H: BI with support values

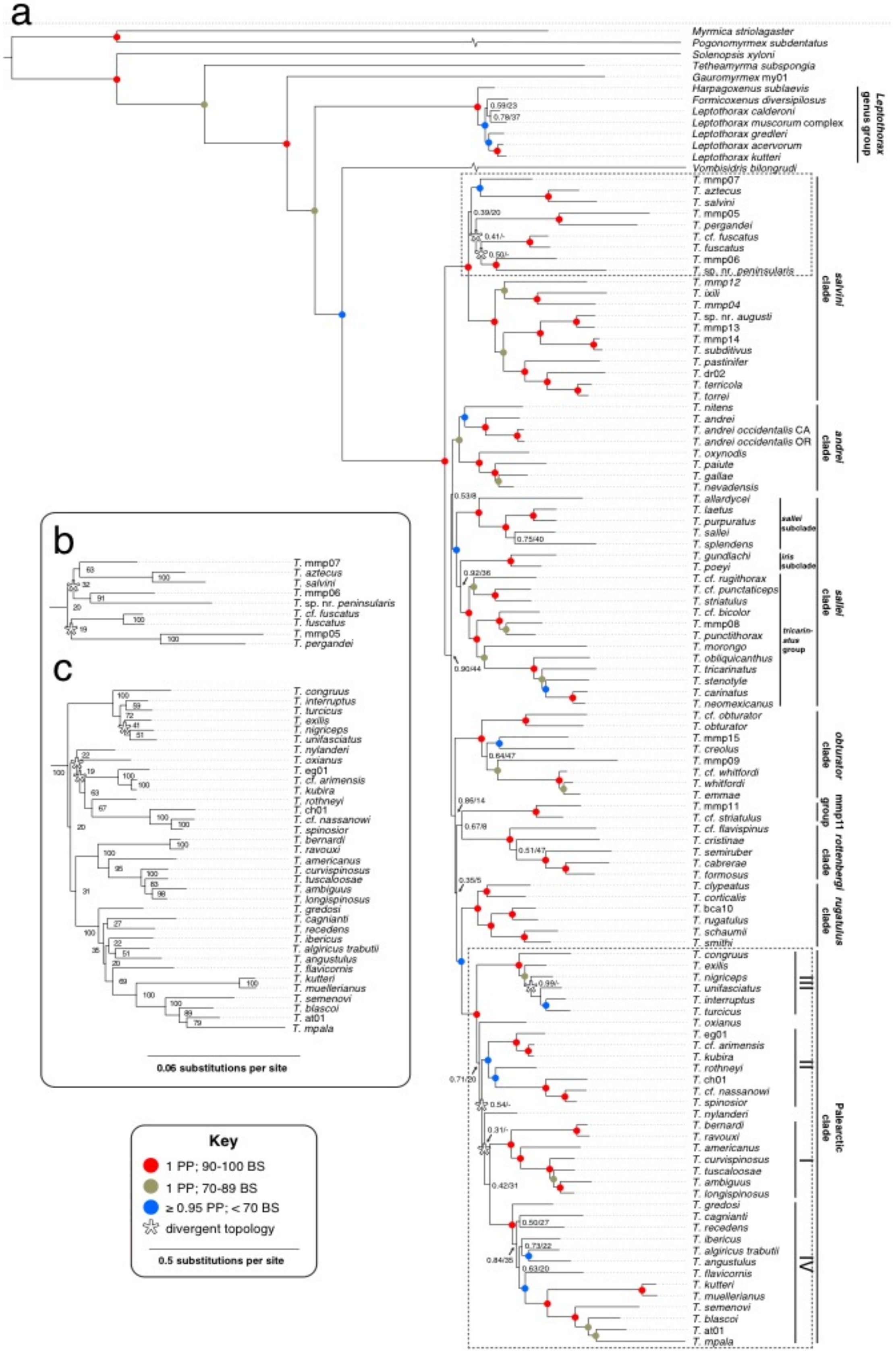

Additional file 7. Best Bayesian inference (BI, node support values shown as posterior probability), maximum likelihood (ML, node support values in bootstraps), and gene concordance trees estimated from analysis of the concatenated, unconstrianed 10-gene Sanger sequencing dataset, with data sub-setting experiments. **A**: BI tree inferred with MrBayes from the full dataset; **B**: ML tree inferred with IQTREE from the full dataset; **C**: ML tree inferred with RAxML from the full dataset; **D**: RAxML tree, introns removed; **E**: RAxML tree, COI + II removed; **F**: RAxML tree, COI + II and introns removed; **G**: gene concordance tree inferred with BUCKy, branch lengths and branch support values in concordance factor units; **H**: (**a)** Bayesian inference (BI) phylogeny of the ant genus *Temnothorax*, including myrmicine outgroups. Node support values are given in Bayesian posterior probability (PP) and maximum likelihood bootstraps (BS); the latter were generated in a separate maximum likelihood (ML) analysis. Actual support values are noted as PP/BS for all nodes that do not conform to the three categories provided in the key. The outlined parts of the phylogeny contain nodes which diverged topologically among BI and ML analyses, indicated by asterisks; the latter alternate topologies are depicted in the inset. **(b)** and **(c)**, alternate topologies resulting from ML analysis; node support values are given in BS.
