# Supplementary material for: Insights into the evolution, biogeography and natural history of the acorn ants, genus Temnothorax Mayr (hymenoptera: Formicidae)
Source: BMC Evol Biol. 2017 Dec 13;17:250. doi: 10.1186/s12862-017-1095-8 (PMC5729518; doi:10.1186/s12862-017-1095-8)
Supplement: Supplementary file 9 — Definitions and illustrations of morphological characters used in this study. (DOCX 101 kb) [file 12862_2017_1095_MOESM9_ESM.docx]

**Additional file 9. Morphological characters and indices**

TSC transverse crest on the maxillary stipes (see Fig. 2 in main article).

0 = absent

1 = present

PF palp formula = number of maxillary segments, number of labial segments.

0 = 3,2

1 = 4,2

2 = 4,3

3 = 5,3

MTC mandibular tooth count

0 = 0

1 = 1

2 = 4

3 = 5

4 = 6

MCC median clypeal carina

0 = absent

1 = present

CMD anterior clypeal margin concave medially

0 = false

1 = true

AC antennomere count.

0 = 11

1 = 12

SSM in full face view, antennal scape surpassing margin of head when fully retracted.

0 = false

1 = true

CL cephalic length: In full face view, maximum length of head from anterior margin

of clypeus to posterior margin of the head.

0 = < 0.8 mm

1 = 0.8 - 1 mm

2 = > 1 mm

WL Weber’s length: in profile view, diagonal length of mesosoma in profile, from anterior declivity of pronotum (exclusive of pronotal ‘‘neck’’) to apex of metapleural lobe.

0 = < 1 mm

1 = 1 - 1.2 mm

2 = > 1.2 mm

MTG metanotal groove: in profile view, impression between propodeum and mesonotum.

0 = absent

1 = present

MSh shape of mesosoma dorsum in profile.

0 = straight

1 = propodeum depressed

2 = evenly rounded

MSC mesosoma setae count.

0 = 0-20

1 = 22-40

2 = > 42

MSSh mesosoma setae shape.

0 = blunt-tipped

1 = clavate

2 = tapering

NOSh petiolar node shape in profile (see Fig. 1).

0 = cuneiform

1 = subquadrate

2 = quadrate

3 = rounded

4 = squamiform

APT anterior peduncle teeth: petiole with a pair of prominences located antero-dorsally, on the peduncle; often these are connected by a transverse ridge (see Fig. 2).

0 = absent

1 = present

PPSh postpetiolar shape in dorsal view (see Fig. 3).

0 = subquadrate

1 = trapezoidal

2 = campaniform

3 = globular

**Indices**

SI scape index (SL/CL*100). SL = scape length: maximum straight-line length of scape shaft exclusive of basal condylar bulb and shaft.

0 = < 80

1 = ≥ 80

A1I antennomere 1 length index (A1/SL * 100). A1 = antennomere 1 length: length of the distal-most antennal segment.

0 = < 50

1 = ≥ 50

CbLI antennal club length index (CL/SL * 100). CbL = antennal club length: combined length of the distal-most three antennal segments.

0 = < 80

1 = ≥ 80

CWI cephalic width index (CW/CWb*100). CW = cephalic width: maximum width of head in full face view, including the compound eyes. CWb = *sensu* Csősz *et al.* 2014: maximum width of head in full face view, measured just posterior to the compound eyes.

0 = < 110

1 = ≥ 110

FRSI frontal carinae distance index (FRS/CL * 100). FRS = *sensu* Csősz *et al.* 2014: Distance of the frontal carinae immediately caudal of the posterior intersection points between frontal carinae and the lamellae dorsal of the torulus. If these dorsal lamellae do not laterally surpass the frontal carinae, the deepest point of scape corner pits may be taken as reference line. These pits take up the inner corner of scape base when the scape is fully switched caudad and produce a dark triangular shadow in the lateral frontal lobes immediately posterior of the dorsal lamellae of scape joint capsule.

0 = < 32.5

1 = ≥ 32.5

PLI petiole length index (PL/WL * 100). PL = petiole length *sensu* Csősz *et al*. 2014: petiole length measured in dorsal view distance between the dorsalmost point of caudal petiolar margin and the dorsalmost point of anterior petiolar peduncle at the transversal level of its strongest constriction. Both points have to be positioned in the same horizontal plane (focal level).

0 = < 35

1 = 35-39

2 = 40-45

3 = > 45

NI petiole node index (NOL/NOH * 100). NOL = *sensu* Csősz *et al.* 2014: Length of the petiolar node, measured from petiolar spiracle to dorso-caudal corner of caudal cylinder. (Note: do not erroneously take as the reference point the dorso-caudal corner of the helcium, which is sometimes visible). NOH = *sensu* Csősz *et al.* 2014: Maximum height of the petiolar node, measured from the uppermost point of the petiolar node perpendicular to a reference line set from the petiolar spiracle to the dorso-caudal corner of caudal cylinder of the petiole.

0 = < 88

1 = ≥ 88

PLI postpetiole length index (PL/PPL * 100). PPL = maximum length of the postpetiole, measured in profile view.

0 = < 200

1 = ≥ 200

PWI pronotum width index (PW/CL * 100). PW = pronotal width: maximum width of pronotum, measured in dorsal view.

0 = < 60

1 = ≥ 60

PPWI1 postpetiole index 1 (PPW/PTW * 100). PPW = postpetiole width: maximum width of postpetiole, measured in dorsal view. PTW = petiole width: maximum width of petiole, measured in dorsal view.

0 = < 150

1 = ≥ 150

PPWI2 postpetiole index 2 (PPW/PW * 100).

0 = < 61

1 = ≥ 61

MSLI mesosoma setae length index (MSL/CL * 100). MSL = measured in profile view, the length of the longest setae on the mesosoma.

0 = < 15

1 = ≥ 15

HFWI hind femora width index (HFW/HTW). HFW = maximum width of the hind femur, measured in dorsal view. HTW = maximum width of the hind trochanter, measured in dorsal view.

0 = < 2.5

1 = 2.5 – 3

2 = > 3

**Figure 1.** Petiole node shapes in profile view used in the morphological analysis. Scale bars equivalent to 0.1 mm. **A:** *T. nitens*, CASENT0005686 **B:** *T. rugatulus,* CASENT0005690 **C:** *T. silvestrii,* CASENT0172599 **D:** *T. emmae,* CASENT0102836 **E:** *T. subditivus,* CASENT0104742.

**Figure 2**. Petioles in ¾ dorsal view, illustrating presence and absence of APT. Scale bars equivalent to 0.1 mm. **A:** *T. nevadensis,* CASENT0756606 **B:** *T. aztecus,* CASENT0733318.

**Figure 3.** Waist segments in dorsal view, illustrating postpetiole node shapes used in the morphological analysis. Scale bars equivalent to 0.1 mm. **A:** *T. ambiguus*, CASENT0104803 **B:** *T. flavicornis*, CASENT0906707 **C:** *T. splendens*, CASENT0636109 **D:** *T. rottenbergi*, CASENT0912989.
