# Supplementary material for: Insights into the evolution, biogeography and natural history of the acorn ants, genus Temnothorax Mayr (hymenoptera: Formicidae)
Source: BMC Evol Biol. 2017 Dec 13;17:250. doi: 10.1186/s12862-017-1095-8 (PMC5729518; doi:10.1186/s12862-017-1095-8)
Supplement: Supplementary file 15 — Trees inferred from single-gene Sanger sequencing analyses. (PDF 216 kb) [file 12862_2017_1095_MOESM15_ESM.pdf]

Figure A: *28S*

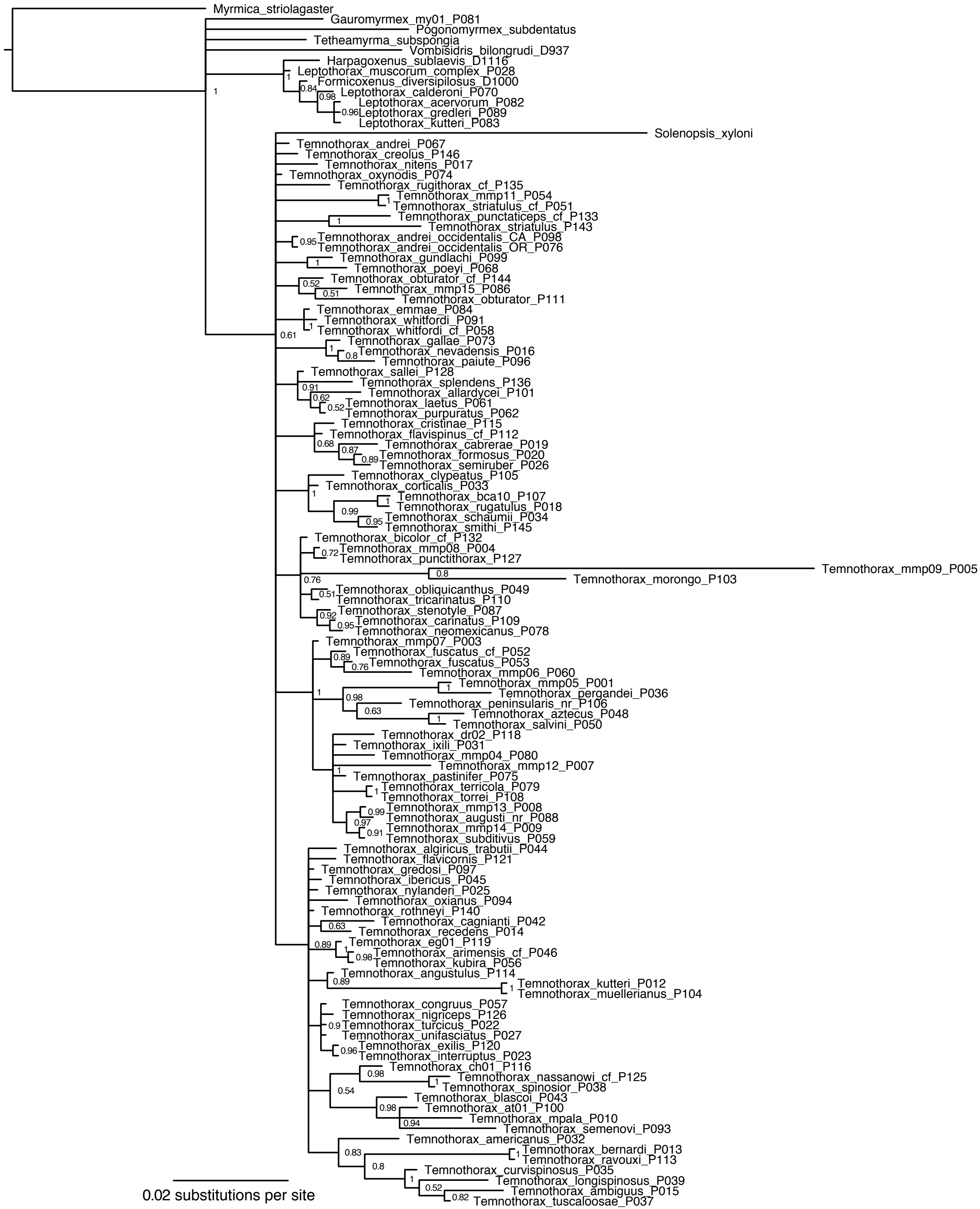
Myrmica_striolagaster
Gauromyrmex_my01_P081
Pogonomyrmex_subdentatus
Tetheamyrma_subspongia
Vombisidris_bilongrudi_D937
Harpagoxenus_sublaevis_D1116
Leptothorax_muscorum_complex_P028
Formicoxenus_diversipilosus_D1000
Leptothorax_calderoni_P070
Leptothorax_acervorum_P082
Leptothorax_gredleri_P089
Leptothorax_kutteri_P083
Solenopsis_xyloni
Temnothorax_andrei_P067
Temnothorax_creolus_P146
Temnothorax_nitens_P017
Temnothorax_oxynodis_P074
Temnothorax_rugithorax_cf_P135
Temnothorax_mmp11_P054
Temnothorax_striatulus_cf_P051
Temnothorax_punctaticeps_cf_P133
Temnothorax_striatulus_P143
Temnothorax_andrei_occidentalis_CA_P098
Temnothorax_andrei_occidentalis_OR_P076
Temnothorax_gundlachi_P099
Temnothorax_poeyi_P068
Temnothorax_obturator_cf_P144
Temnothorax_mmp15_P086
Temnothorax_obturator_P111
Temnothorax_emmae_P084
Temnothorax_whitfordi_P091
Temnothorax_whitfordi_cf_P058
Temnothorax_gallae_P073
Temnothorax_nevadensis_P016
Temnothorax_paiute_P096
Temnothorax_sallei_P128
Temnothorax_splendens_P136
Temnothorax_allardycei_P101
Temnothorax_laetus_P061
Temnothorax_purpuratus_P062
Temnothorax_cristinae_P115
Temnothorax_flavispinus_cf_P112
Temnothorax_cabrerae_P019
Temnothorax_formosus_P020
Temnothorax_semiruber_P026
Temnothorax_clypeatus_P105
Temnothorax_corticalis_P033
Temnothorax_bca10_P107
Temnothorax_rugatulus_P018
Temnothorax_schaumii_P034
Temnothorax_smithi_P145
Temnothorax_bicolor_cf_P132
Temnothorax_mmp08_P004
Temnothorax_punctithorax_P127
Temnothorax_mmp09_P005
Temnothorax_morongo_P103
Temnothorax_obliquicanthus_P049
Temnothorax_tricarinatus_P110
Temnothorax_stenotyle_P087
Temnothorax_carinatus_P109
Temnothorax_neomexicanus_P078
Temnothorax_mmp07_P003
Temnothorax_fuscatus_cf_P052
Temnothorax_fuscatus_P053
Temnothorax_mmp06_P060
Temnothorax_mmp05_P001
Temnothorax_pergandei_P036
Temnothorax_peninsularis_nr_P106
Temnothorax_aztecus_P048
Temnothorax_salvini_P050
Temnothorax_dr02_P118
Temnothorax_ixili_P031
Temnothorax_mmp04_P080
Temnothorax_mmp12_P007
Temnothorax_pastinifer_P075
Temnothorax_terricola_P079
Temnothorax_torrei_P108
Temnothorax_mmp13_P008
Temnothorax_augusti_nr_P088
Temnothorax_mmp14_P009
Temnothorax_subditivus_P059
Temnothorax_algiricus_trabutii_P044
Temnothorax_flavicornis_P121
Temnothorax_gredosi_P097
Temnothorax_ibericus_P045
Temnothorax_nylanderi_P025
Temnothorax_oxianus_P094
Temnothorax_rothneyi_P140
Temnothorax_cagnianti_P042
Temnothorax_recedens_P014
Temnothorax_eg01_P119
Temnothorax_arimensis_cf_P046
Temnothorax_kubira_P056
Temnothorax_angustulus_P114
Temnothorax_kutteri_P012
Temnothorax_muellerianus_P104
Temnothorax_congruus_P057
Temnothorax_nigriceps_P126
Temnothorax_turcicus_P022
Temnothorax_unifasciatus_P027
Temnothorax_exilis_P120
Temnothorax_interruptus_P023
Temnothorax_ch01_P116
Temnothorax_nassanowi_cf_P125
Temnothorax_spinosior_P038
Temnothorax_blascoi_P043
Temnothorax_at01_P100
Temnothorax_mpala_P010
Temnothorax_semenovi_P093
Temnothorax_americanus_P032
Temnothorax_bernardi_P013
Temnothorax_ravouxi_P113
Temnothorax_curvispinosus_P035
Temnothorax_longispinosus_P039
Temnothorax_ambiguus_P015
Temnothorax_tuscaloosae_P037
0.02 substitutions per site

Figure B: *abdA*

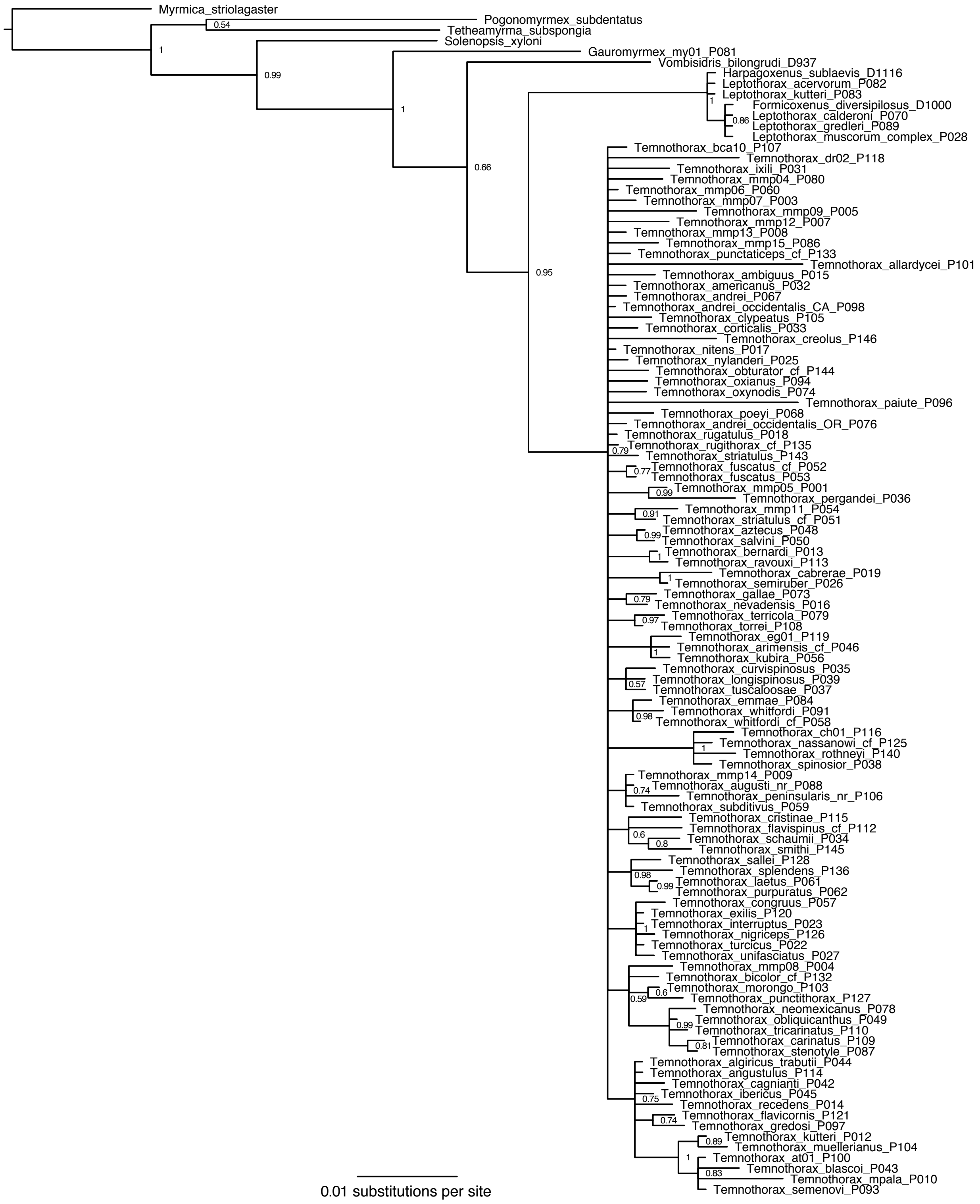

Figure C: *argK*

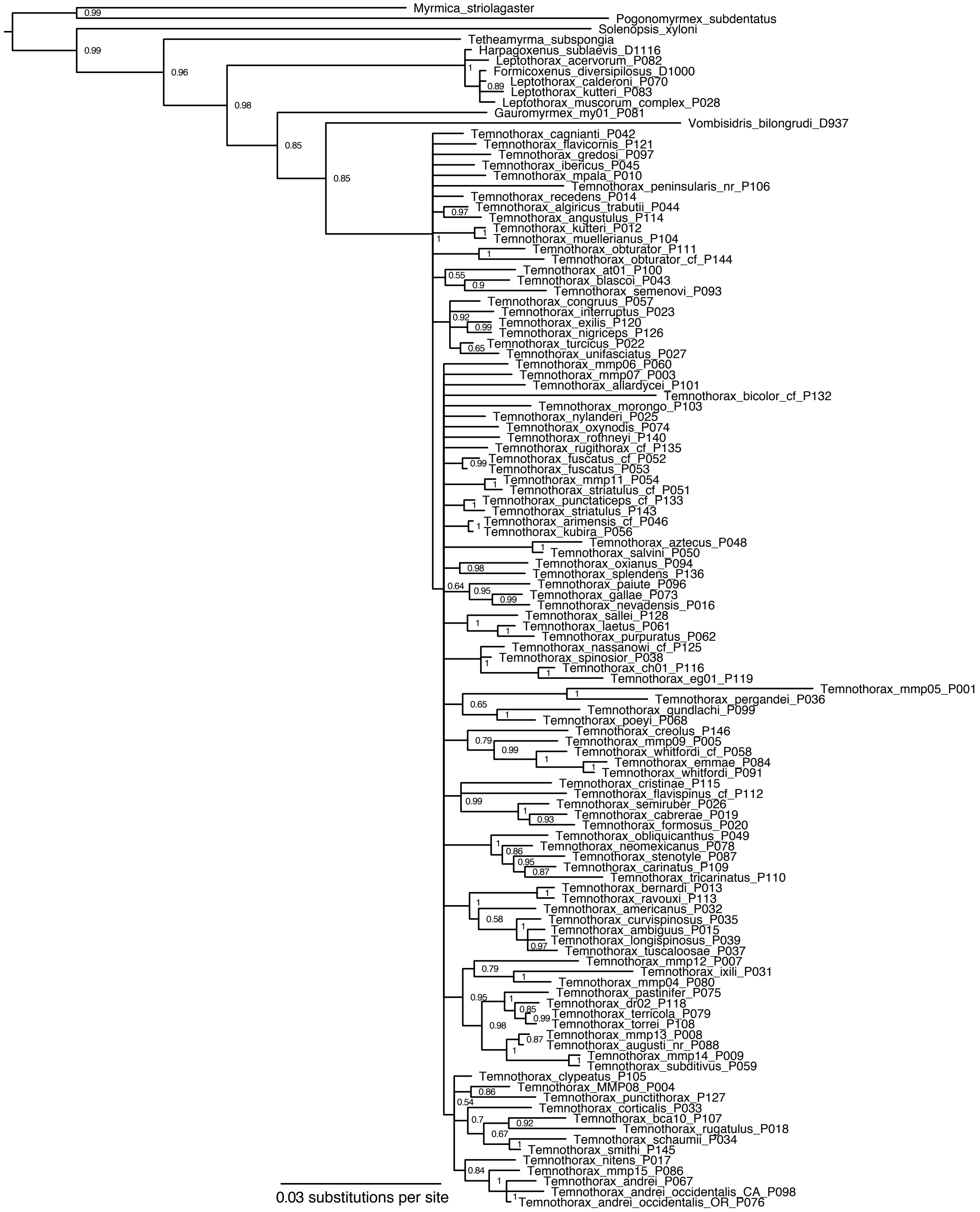

Figure D: *CAD*

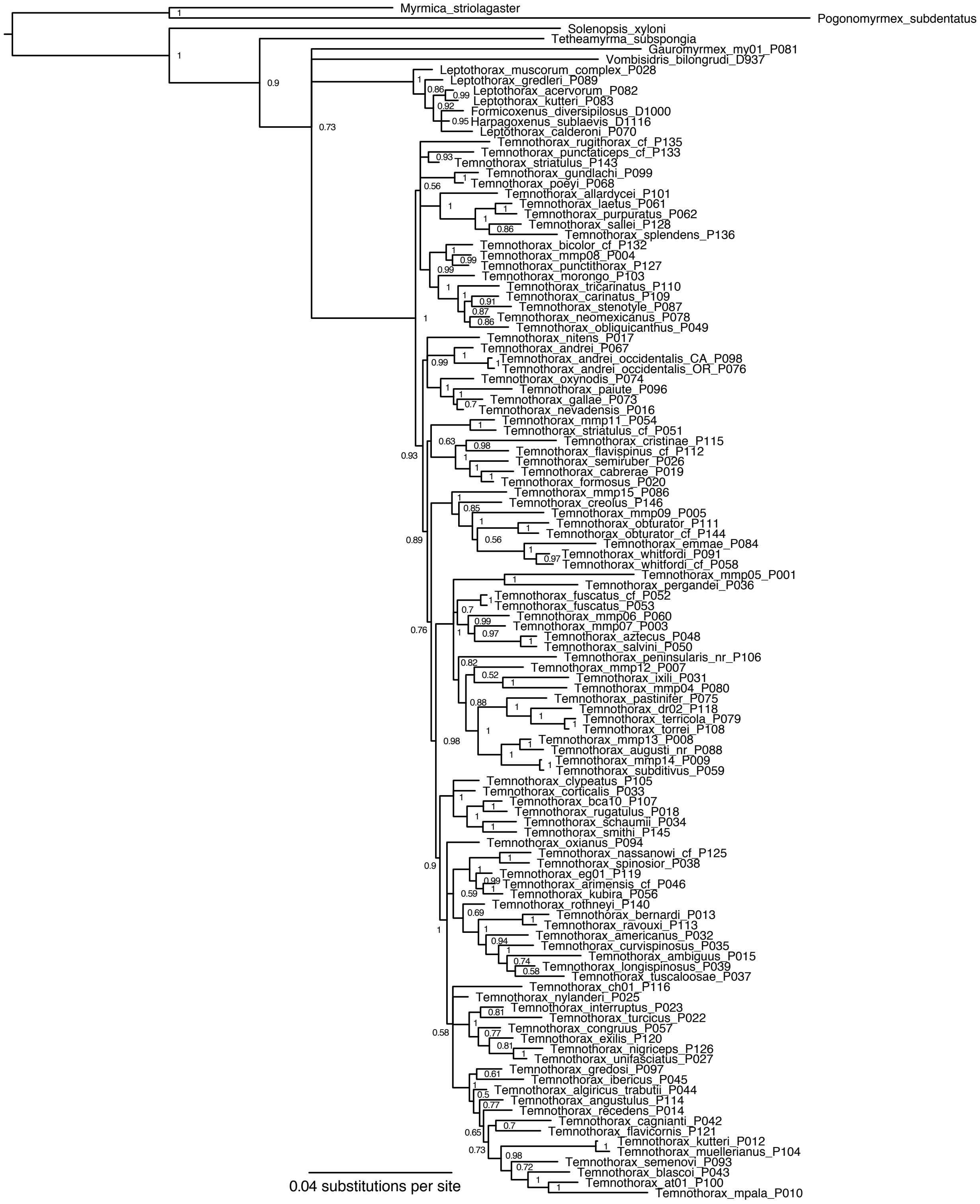
Myrmica_striolagaster
Pogonomyrmex_subdentatus
Solenopsis_xyloni
Tetheamyrma_subspongia
Gauromyrmex_my01_P081
Vombisidris_bilongrudi_D937
Leptothorax_muscorum_complex_P028
Leptothorax_gredleri_P089
Leptothorax_acervorum_P082
Leptothorax_kutteri_P083
Formicoxenus_diversipilosus_D1000
Harpagoxenus_sublaevis_D1116
Leptothorax_calderoni_P070
Temnothorax_rugithorax_cf_P135
Temnothorax_punctaticeps_cf_P133
Temnothorax_striatulus_P143
Temnothorax_gundlachi_P099
Temnothorax_poeyi_P068
Temnothorax_allardycei_P101
Temnothorax_laetus_P061
Temnothorax_purpuratus_P062
Temnothorax_sallei_P128
Temnothorax_splendens_P136
Temnothorax_bicolor_cf_P132
Temnothorax_mmp08_P004
Temnothorax_punctithorax_P127
Temnothorax_morongo_P103
Temnothorax_tricarinatus_P110
Temnothorax_carinatus_P109
Temnothorax_stenotyle_P087
Temnothorax_neomexicanus_P078
Temnothorax_obliquicanthus_P049
Temnothorax_nitens_P017
Temnothorax_andrei_P067
Temnothorax_andrei_occidentalis_CA_P098
Temnothorax_andrei_occidentalis_OR_P076
Temnothorax_oxynodis_P074
Temnothorax_paiute_P096
Temnothorax_gallae_P073
Temnothorax_nevadensis_P016
Temnothorax_mmp11_P054
Temnothorax_striatulus_cf_P051
Temnothorax_cristinae_P115
Temnothorax_flavispinus_cf_P112
Temnothorax_semiruber_P026
Temnothorax_cabrerae_P019
Temnothorax_formosus_P020
Temnothorax_mmp15_P086
Temnothorax_creolus_P146
Temnothorax_mmp09_P005
Temnothorax_obturator_P111
Temnothorax_obturator_cf_P144
Temnothorax_emmae_P084
Temnothorax_whitfordi_P091
Temnothorax_whitfordi_cf_P058
Temnothorax_mmp05_P001
Temnothorax_pergandei_P036
Temnothorax_fuscatus_cf_P052
Temnothorax_fuscatus_P053
Temnothorax_mmp06_P060
Temnothorax_mmp07_P003
Temnothorax_aztecus_P048
Temnothorax_salvini_P050
Temnothorax_peninsularis_nr_P106
Temnothorax_mmp12_P007
Temnothorax_ixili_P031
Temnothorax_mmp04_P080
Temnothorax_pastinifer_P075
Temnothorax_dr02_P118
Temnothorax_terricola_P079
Temnothorax_torrei_P108
Temnothorax_mmp13_P008
Temnothorax_augusti_nr_P088
Temnothorax_mmp14_P009
Temnothorax_subditivus_P059
Temnothorax_clypeatus_P105
Temnothorax_corticalis_P033
Temnothorax_bca10_P107
Temnothorax_rugatulus_P018
Temnothorax_schaumii_P034
Temnothorax_smithi_P145
Temnothorax_oxianus_P094
Temnothorax_nassanowi_cf_P125
Temnothorax_spinosior_P038
Temnothorax_eg01_P119
Temnothorax_arimensis_cf_P046
Temnothorax_kubira_P056
Temnothorax_rothneyi_P140
Temnothorax_bernardi_P013
Temnothorax_ravouxi_P113
Temnothorax_americanus_P032
Temnothorax_curvispinosus_P035
Temnothorax_ambiguus_P015
Temnothorax_longispinosus_P039
Temnothorax_tuscaloosae_P037
Temnothorax_ch01_P116
Temnothorax_nylanderi_P025
Temnothorax_interruptus_P023
Temnothorax_turcicus_P022
Temnothorax_congruus_P057
Temnothorax_exilis_P120
Temnothorax_nigriceps_P126
Temnothorax_unifasciatus_P027
Temnothorax_gredosi_P097
Temnothorax_ibericus_P045
Temnothorax_algiricus_trabutii_P044
Temnothorax_angustulus_P114
Temnothorax_recedens_P014
Temnothorax_cagnianti_P042
Temnothorax_flavicornis_P121
Temnothorax_kutteri_P012
Temnothorax_muellerianus_P104
Temnothorax_semenovi_P093
Temnothorax_blascoi_P043
Temnothorax_at01_P100
Temnothorax_mpala_P010
0.04 substitutions per site

Figure E: *COI + COII*

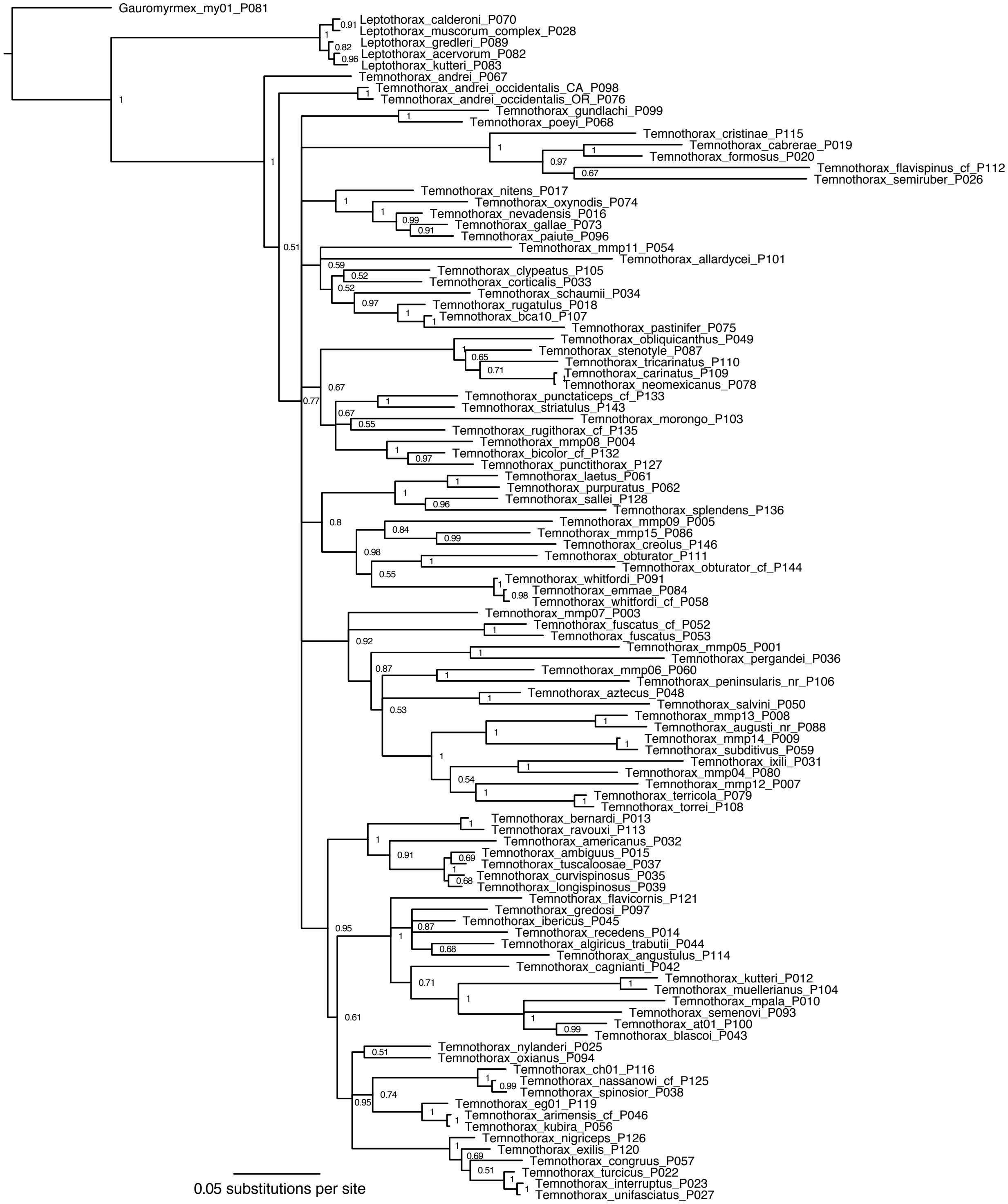

Figure F: *EF1aF2*

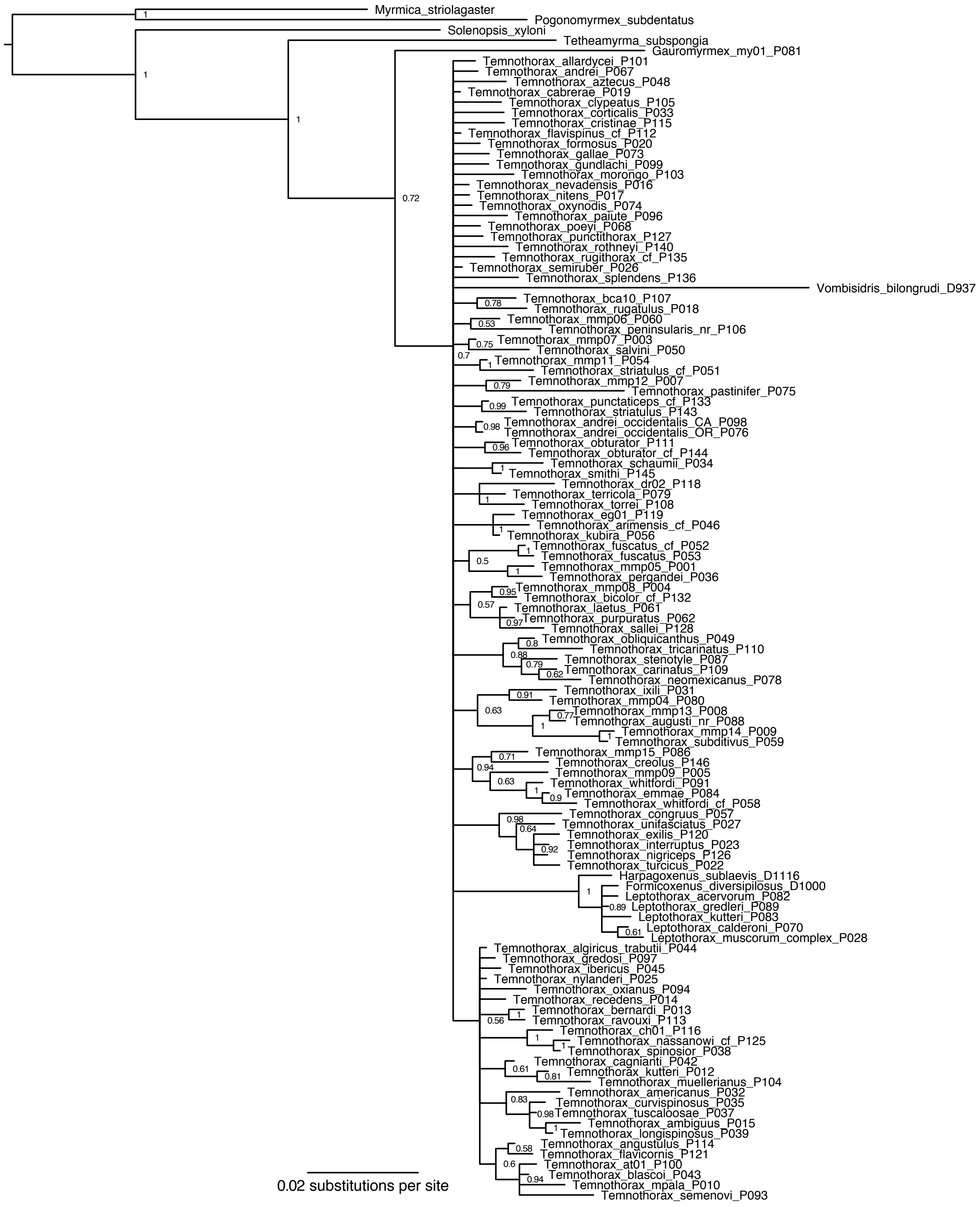

Figure G: *LW Rh*

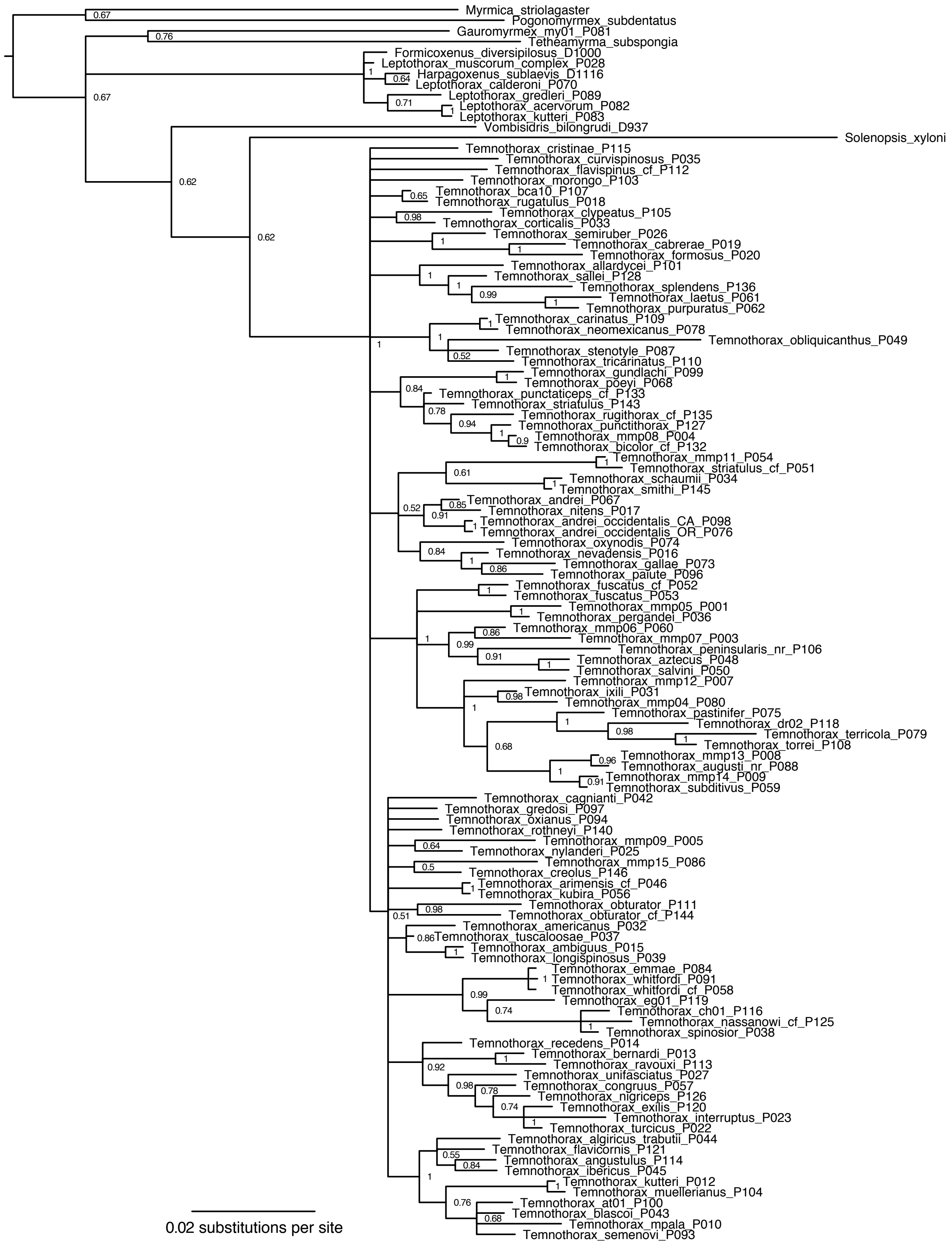

Figure H: *Top1*

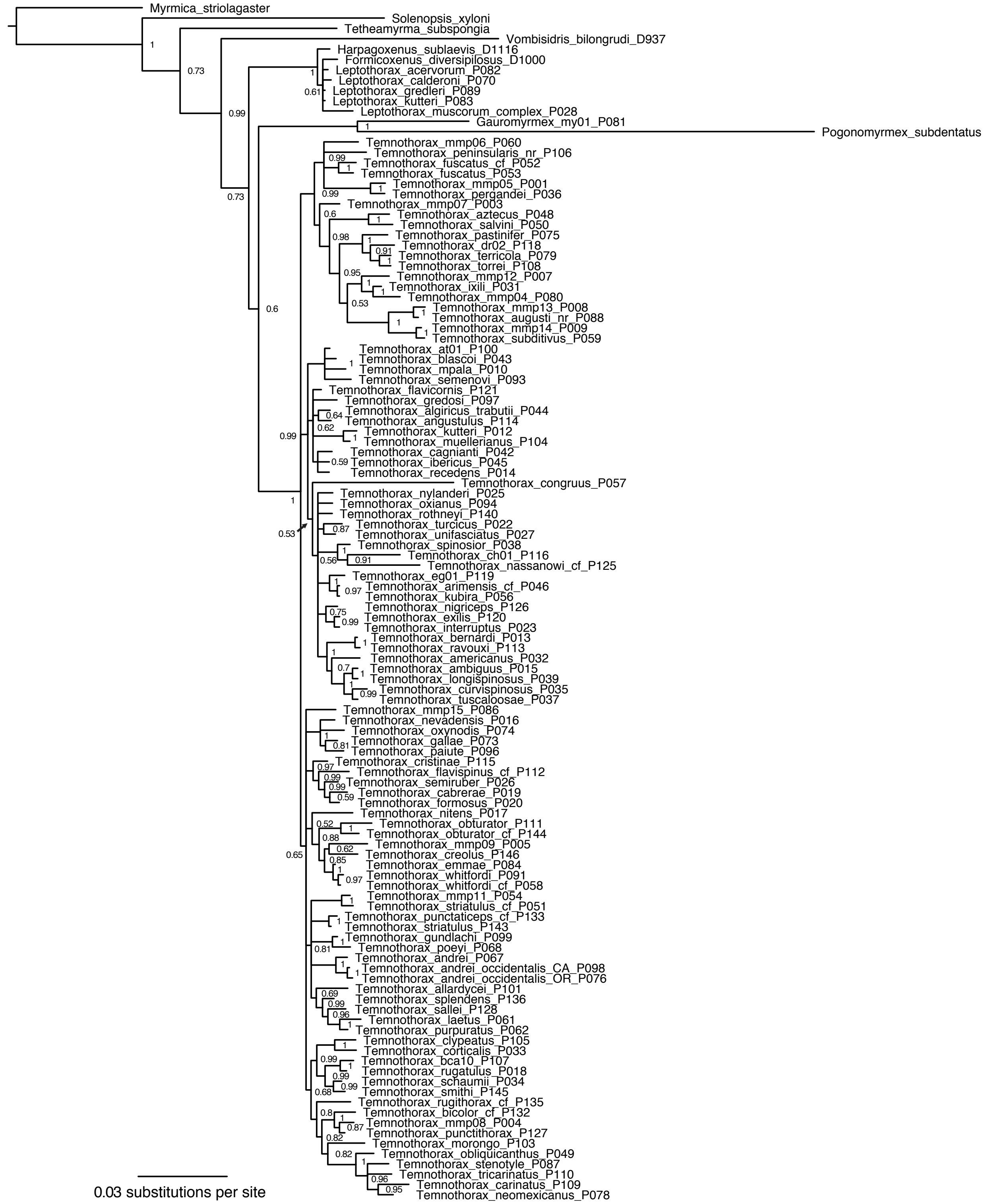

Figure I: *Wg*

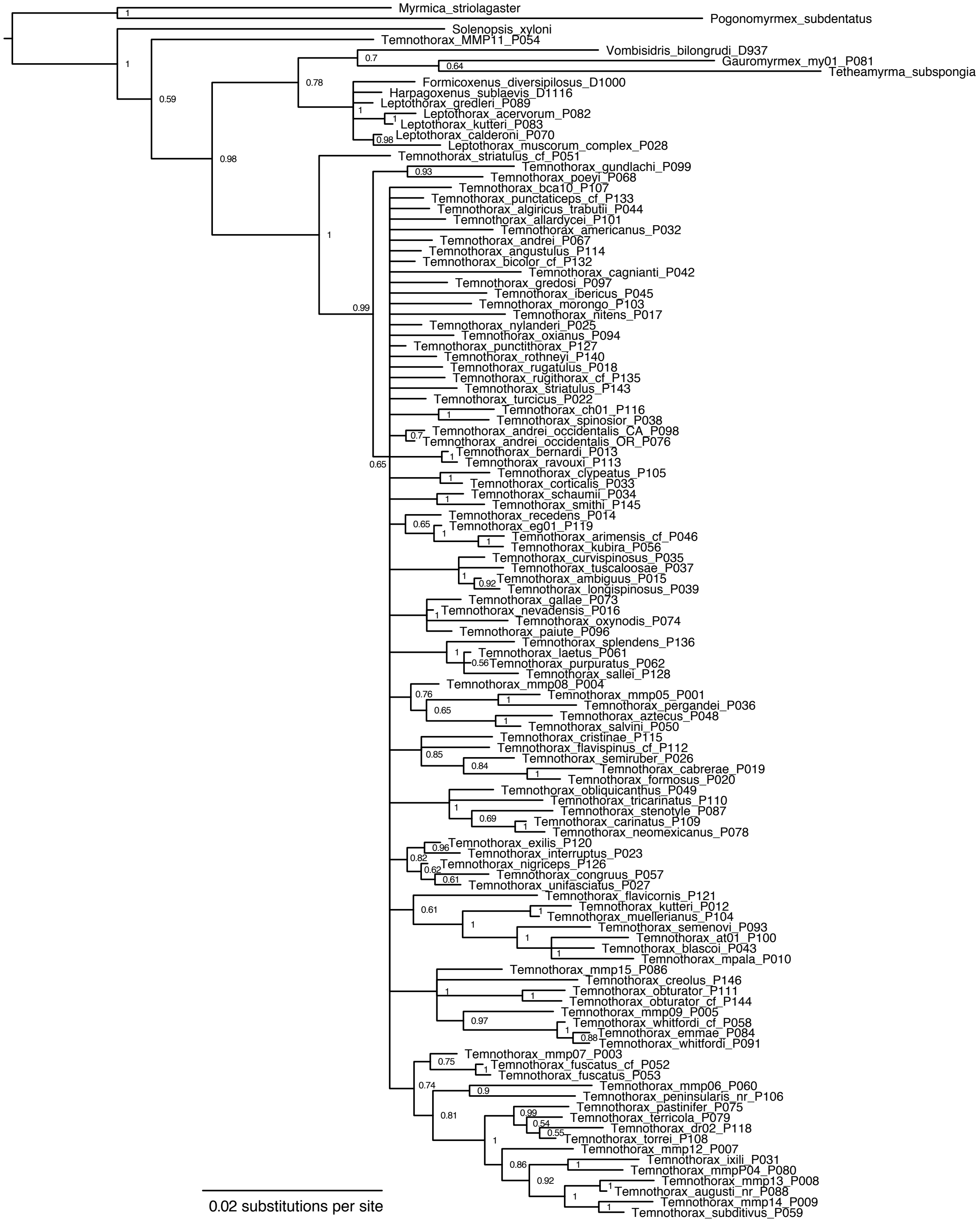

Additional file 15 50% majority-rule consensus trees from MrBayes analyses of single genes. Node support values indicated by posterior probabilities. **A**: *28S*; **B**: *abdA*; **C**: *argK*; **D**: *CAD*; **E**: *COI + COII*; **F**: *EF1aF2*; **G**: *LW Rh*; **H**: *Top1*; **I**: *Wg*.
