# Supplementary material for: Insights into the evolution, biogeography and natural history of the acorn ants, genus Temnothorax Mayr (hymenoptera: Formicidae)
Source: BMC Evol Biol. 2017 Dec 13;17:250. doi: 10.1186/s12862-017-1095-8 (PMC5729518; doi:10.1186/s12862-017-1095-8)
Supplement: Supplementary file 17 — Trees inferred from UCE dataset subsetting experiments. (PDF 460 kb) [file 12862_2017_1095_MOESM17_ESM.pdf]

Figure A: min25

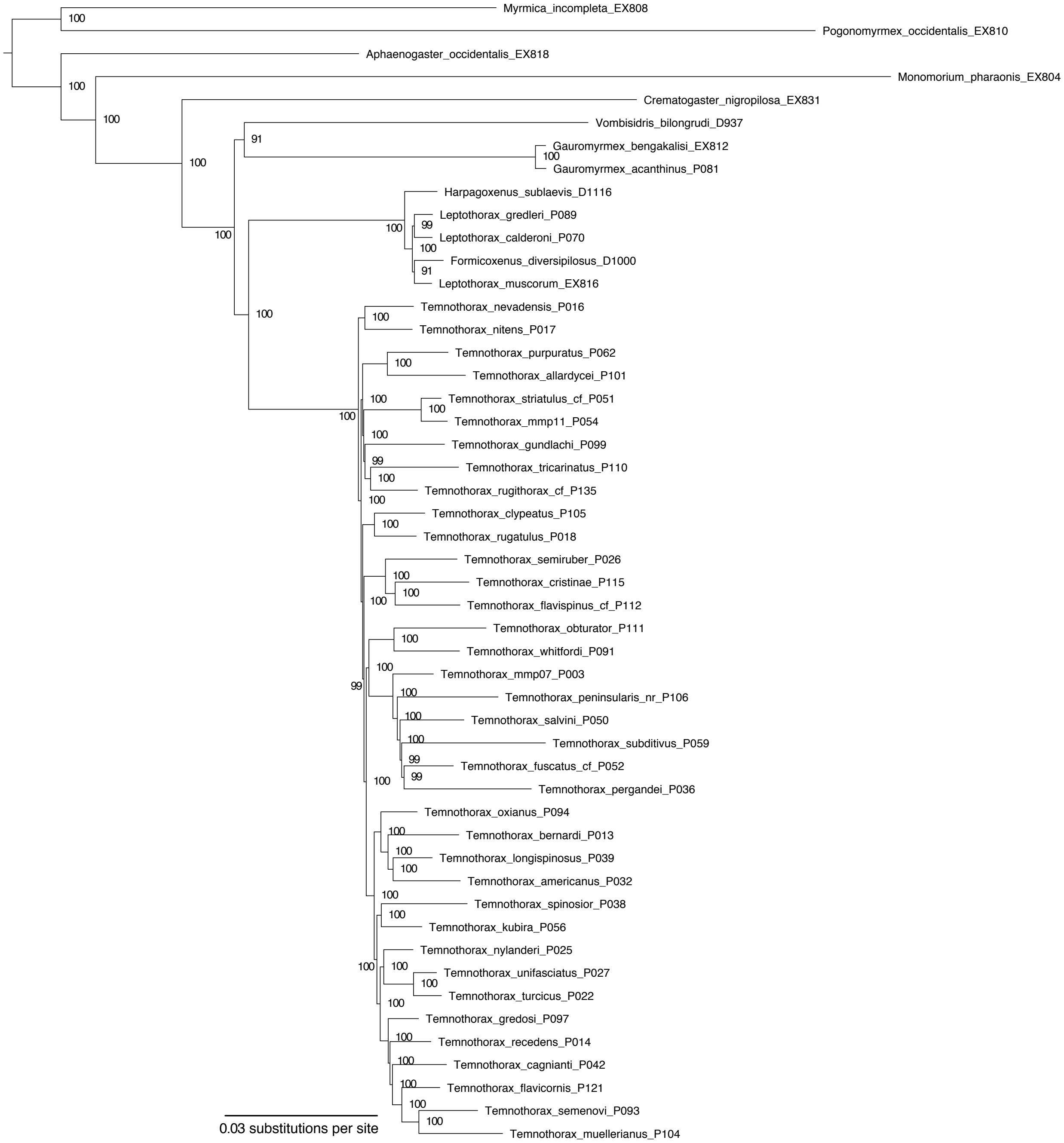

Figure B: min50

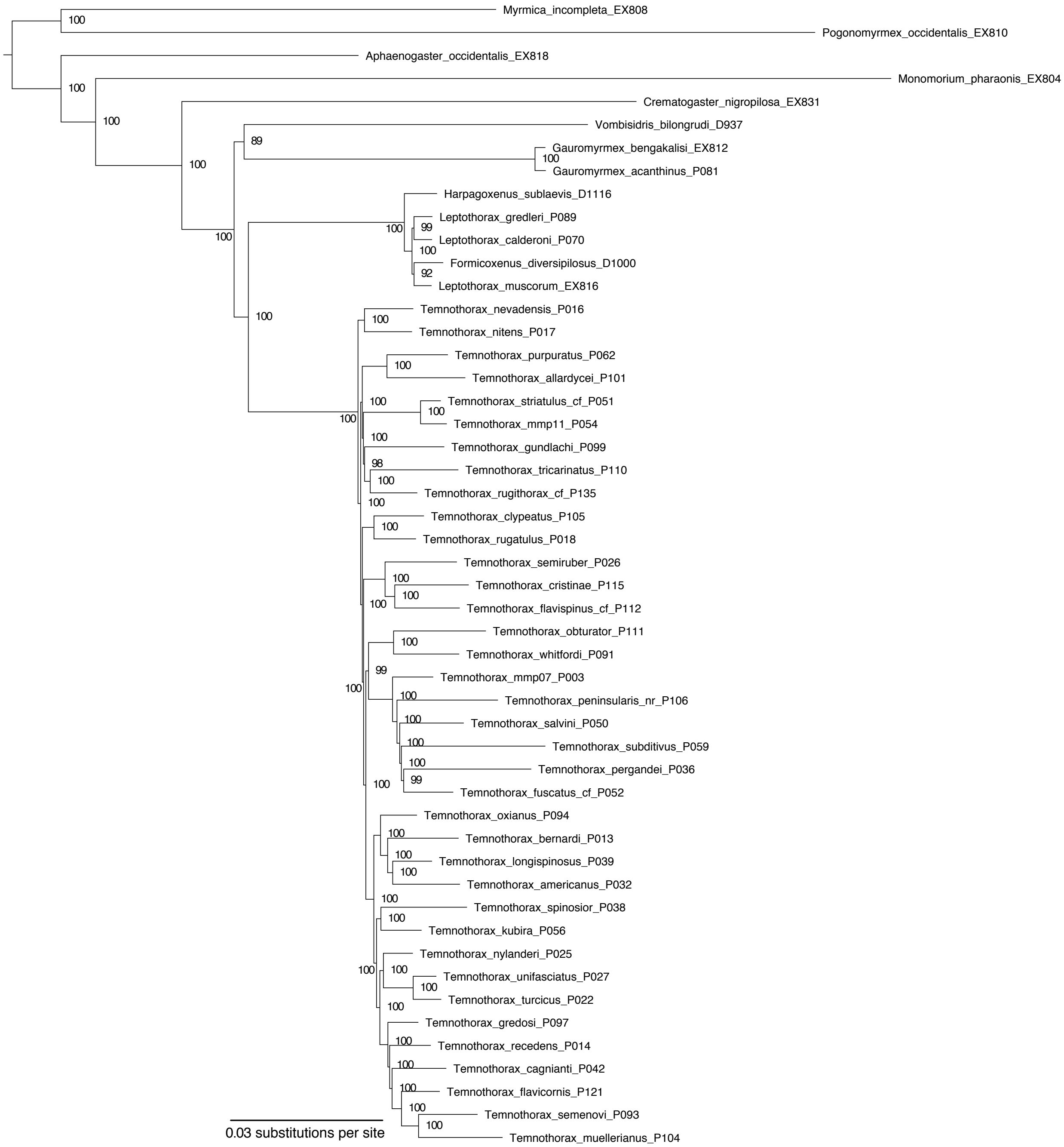

Figure C: min75

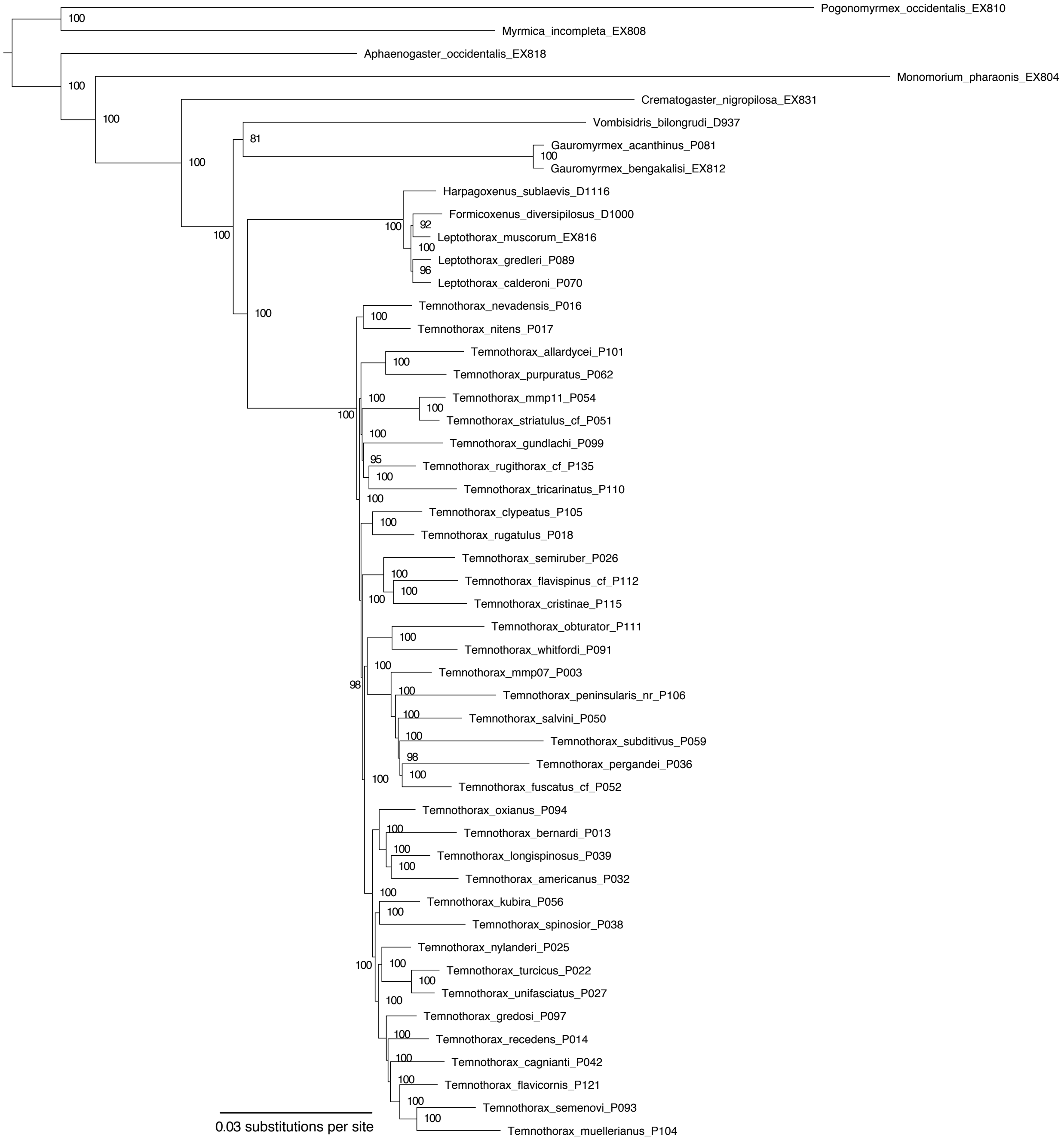

Figure D: min90

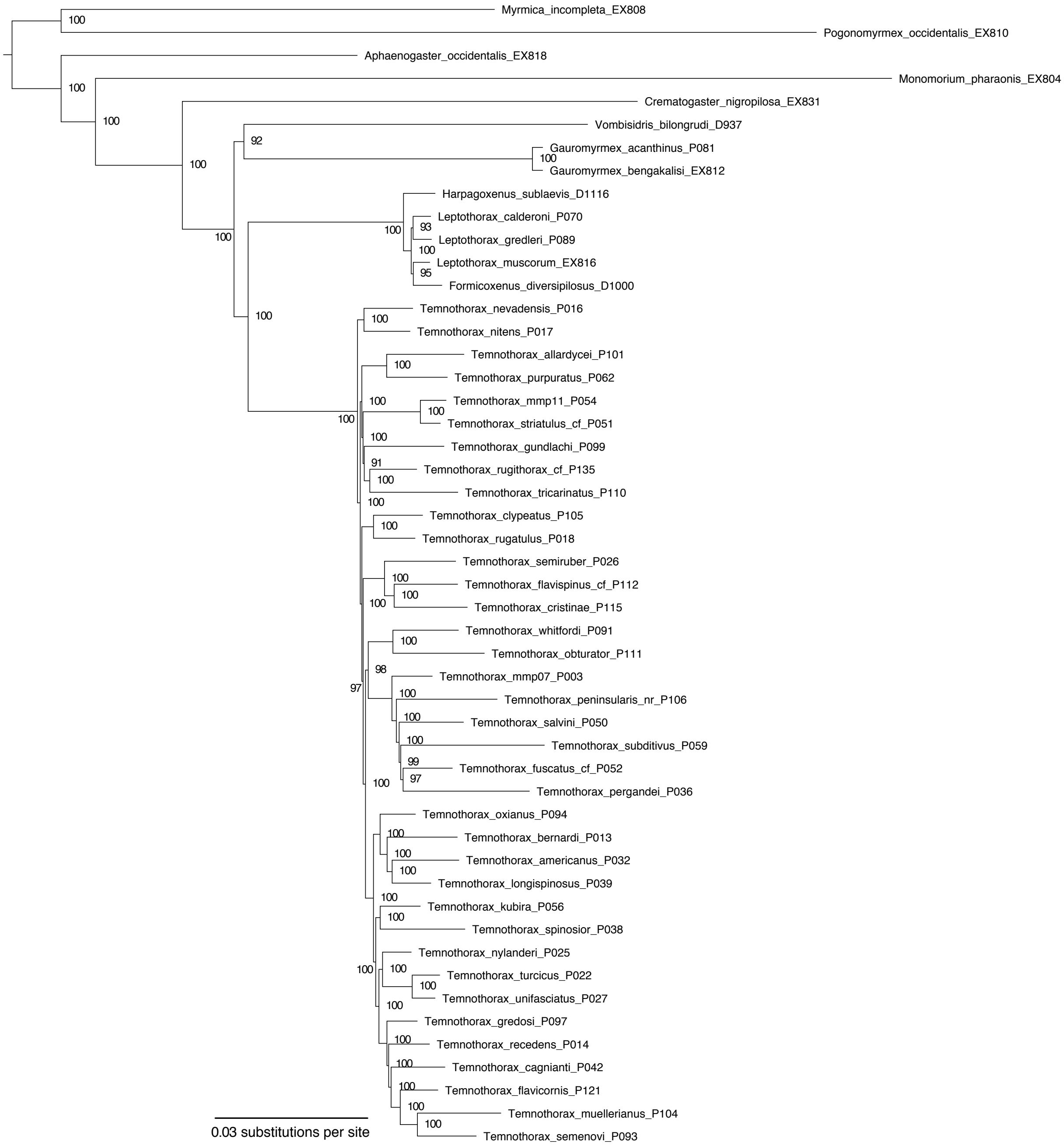

Figure E: min95

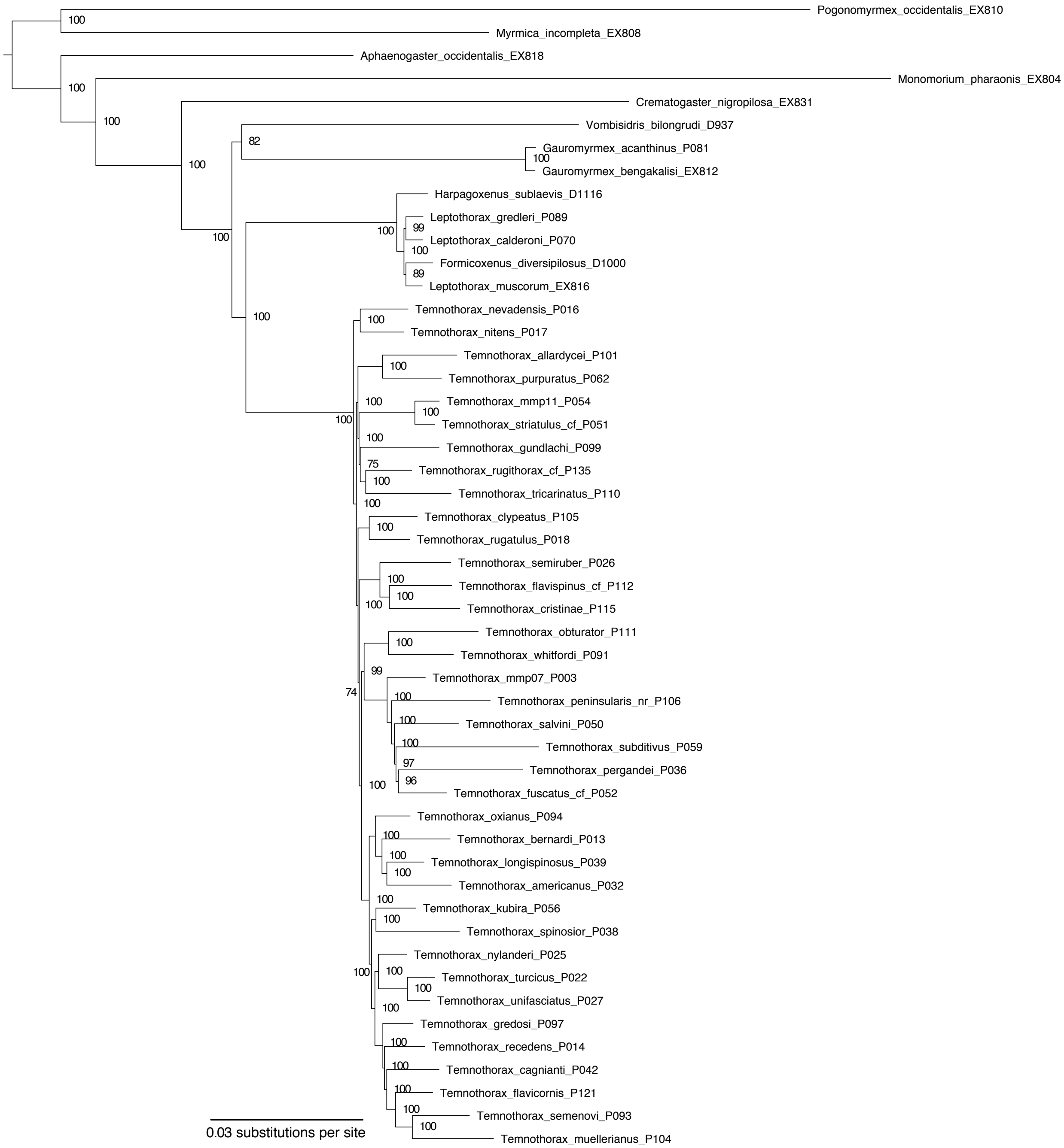

Figure F: min99

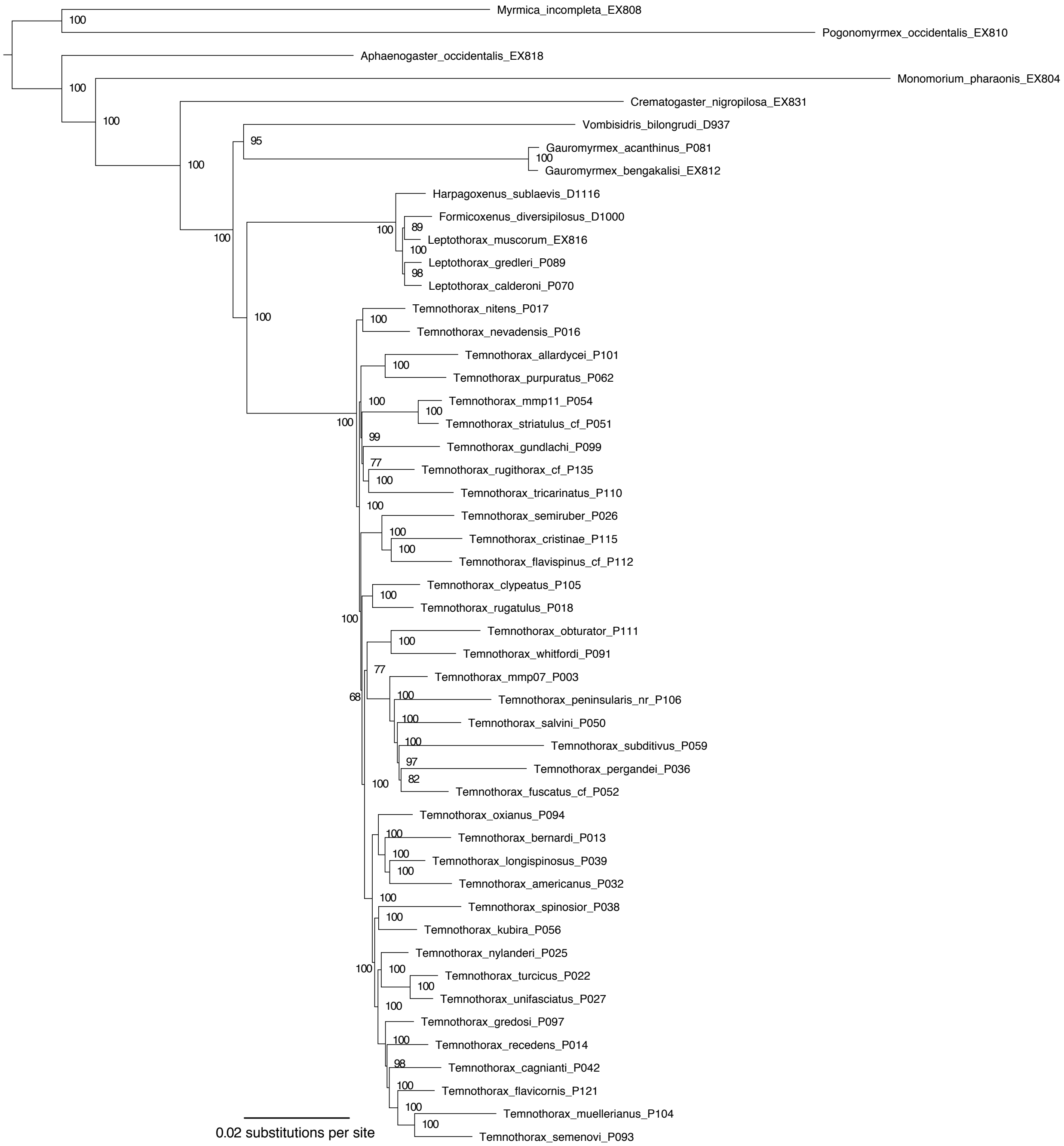

Figure G: min100

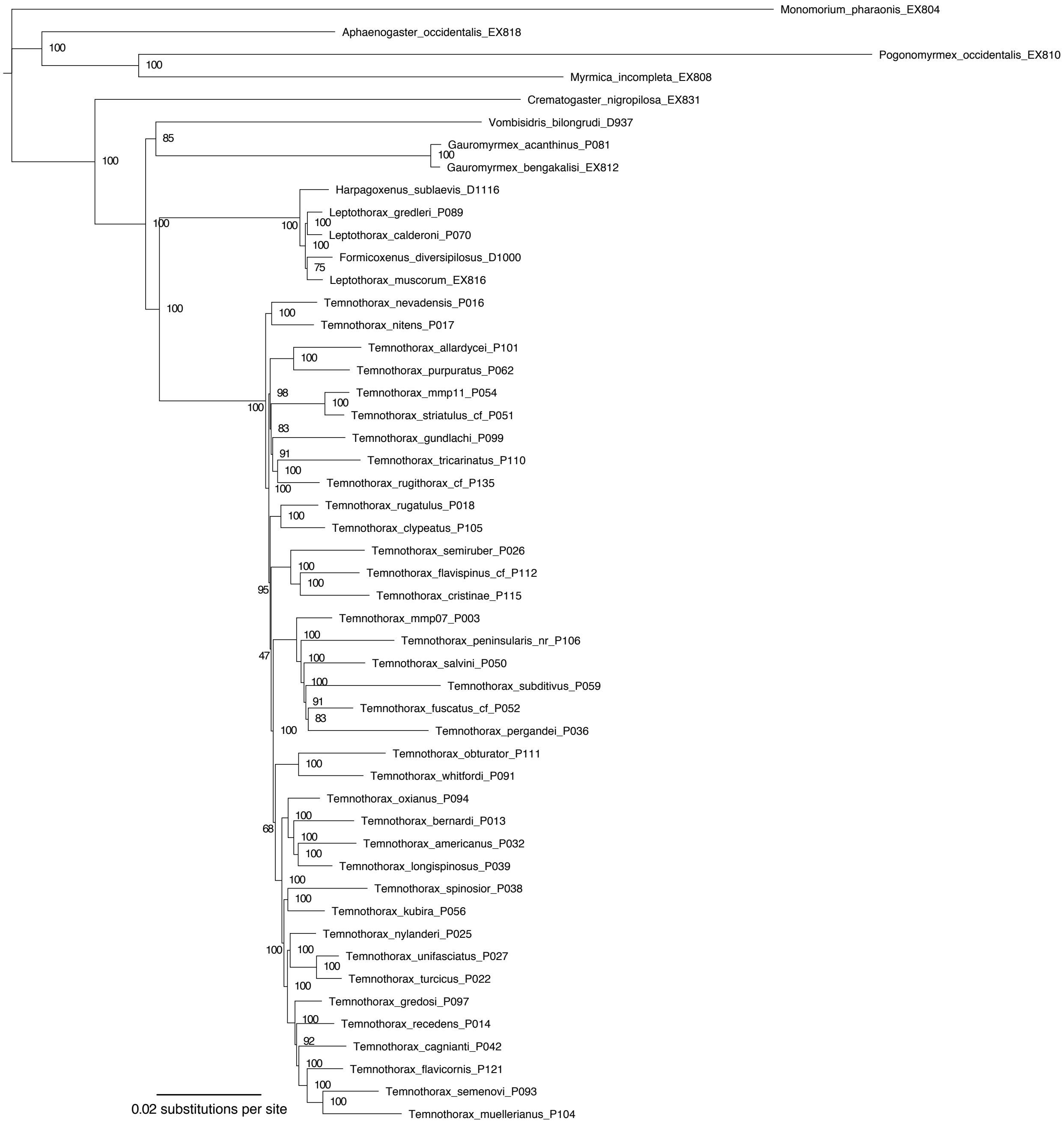

Figure H: rand33_1

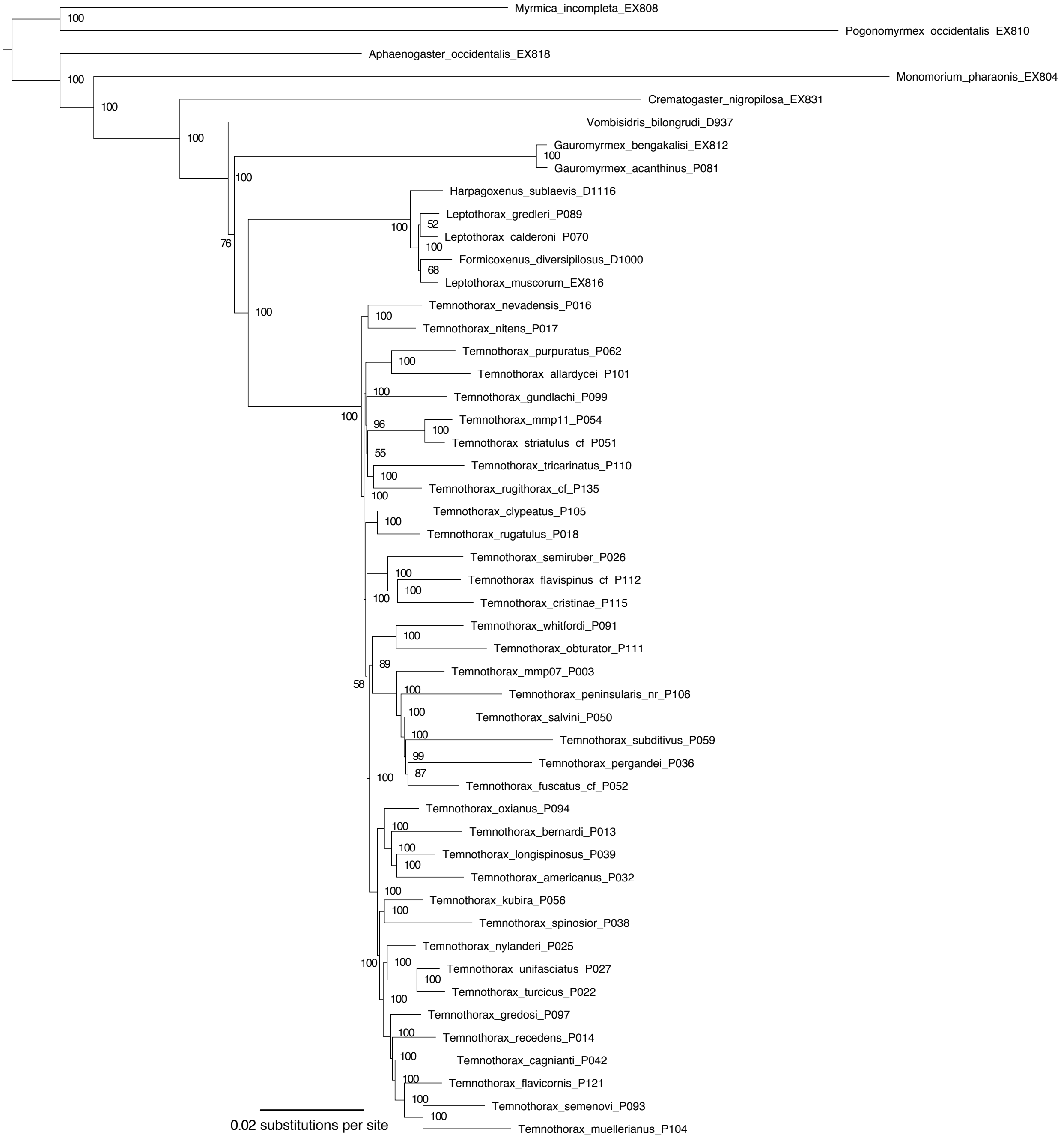

Figure I: rand33_2

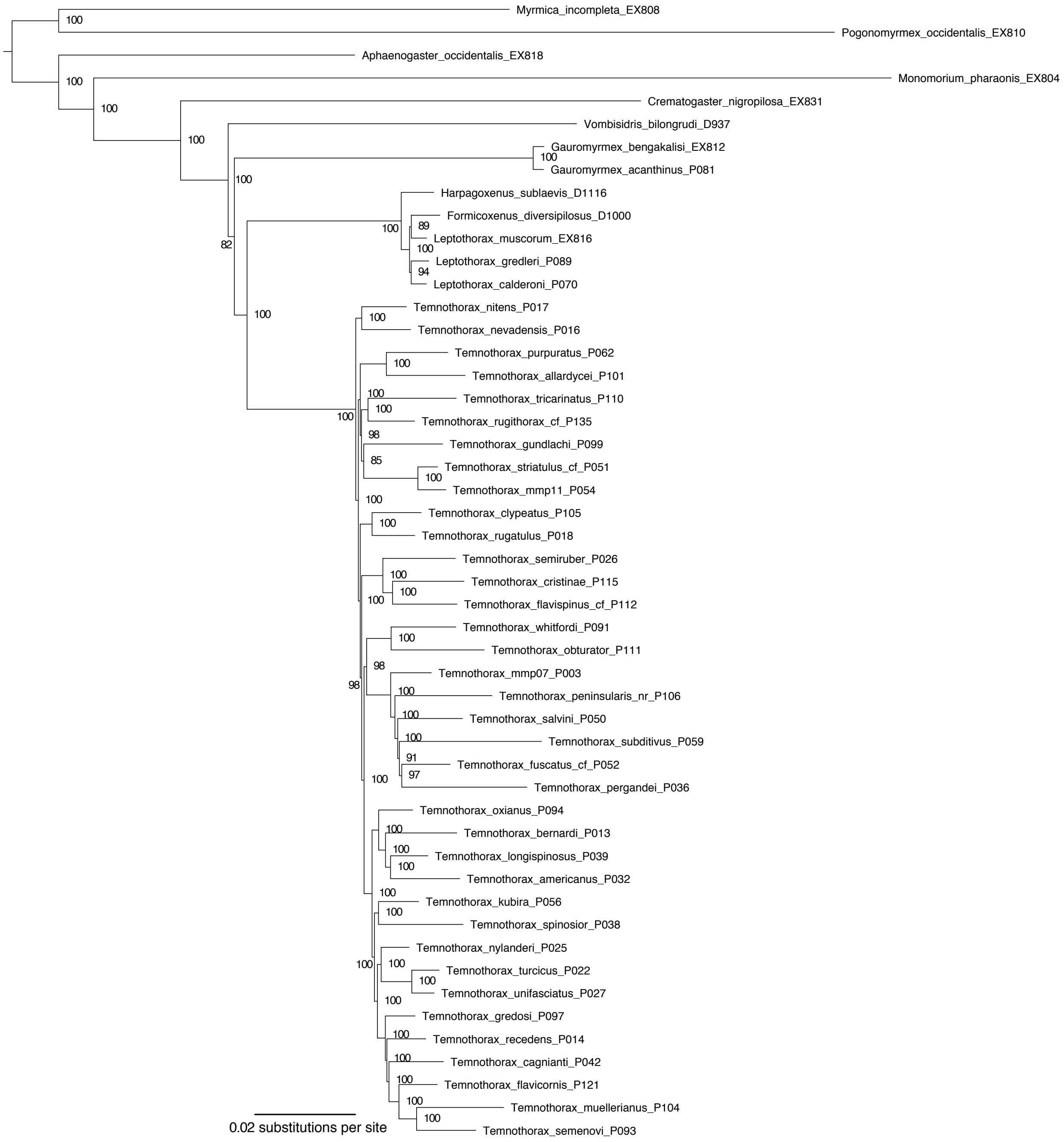

Figure J: rand33_3

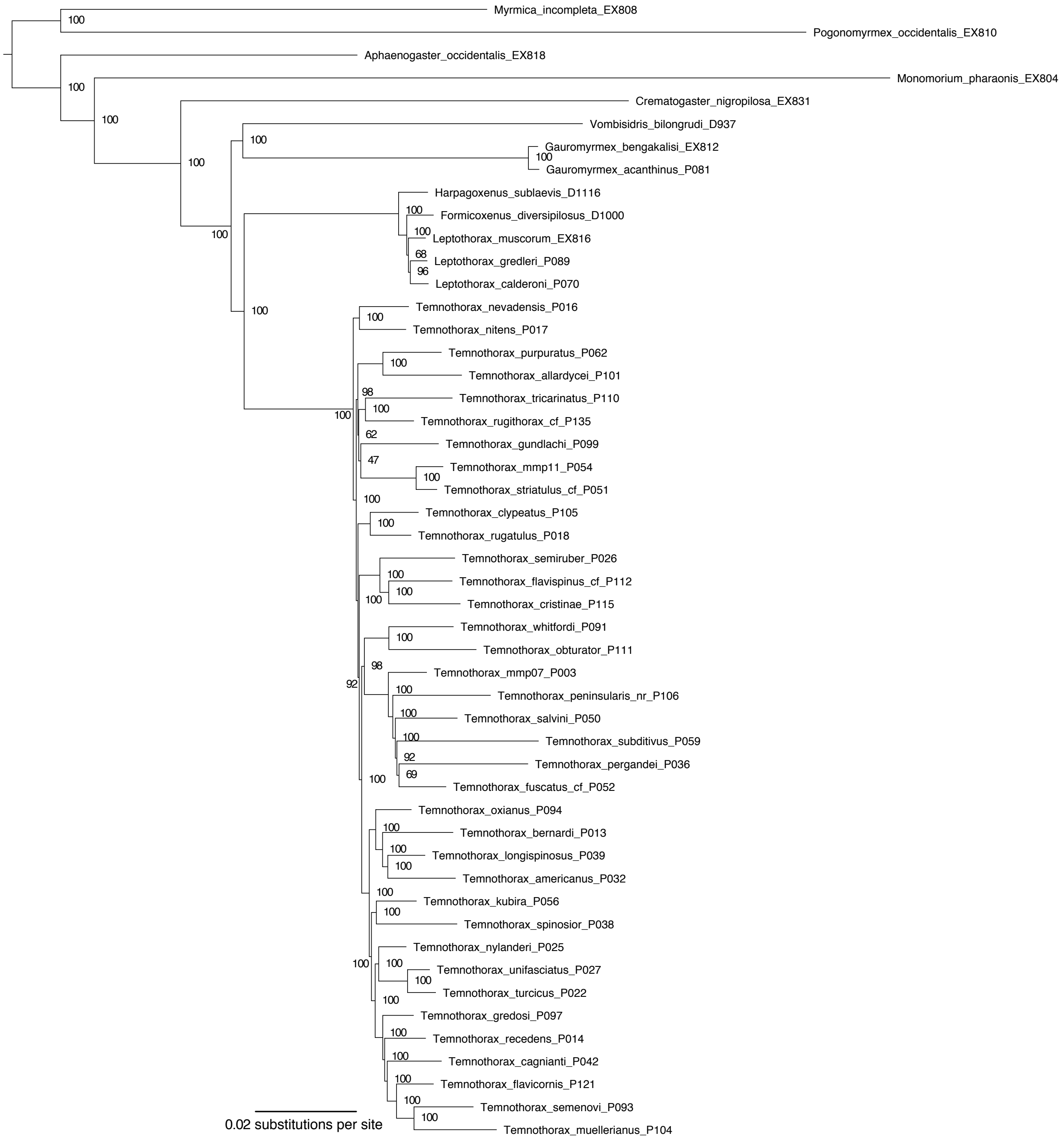

Figure K: rand66

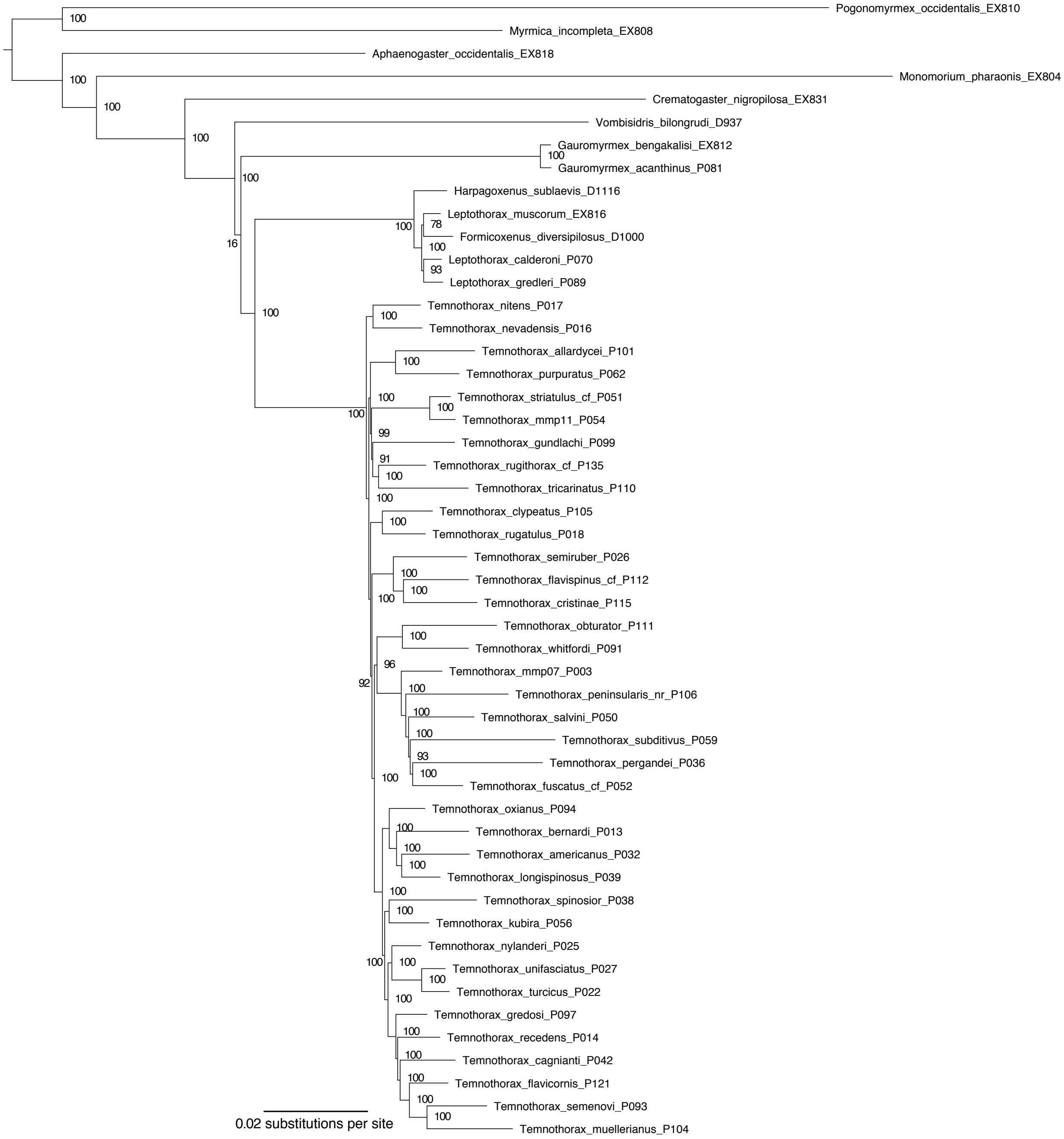

Figure L: rand90

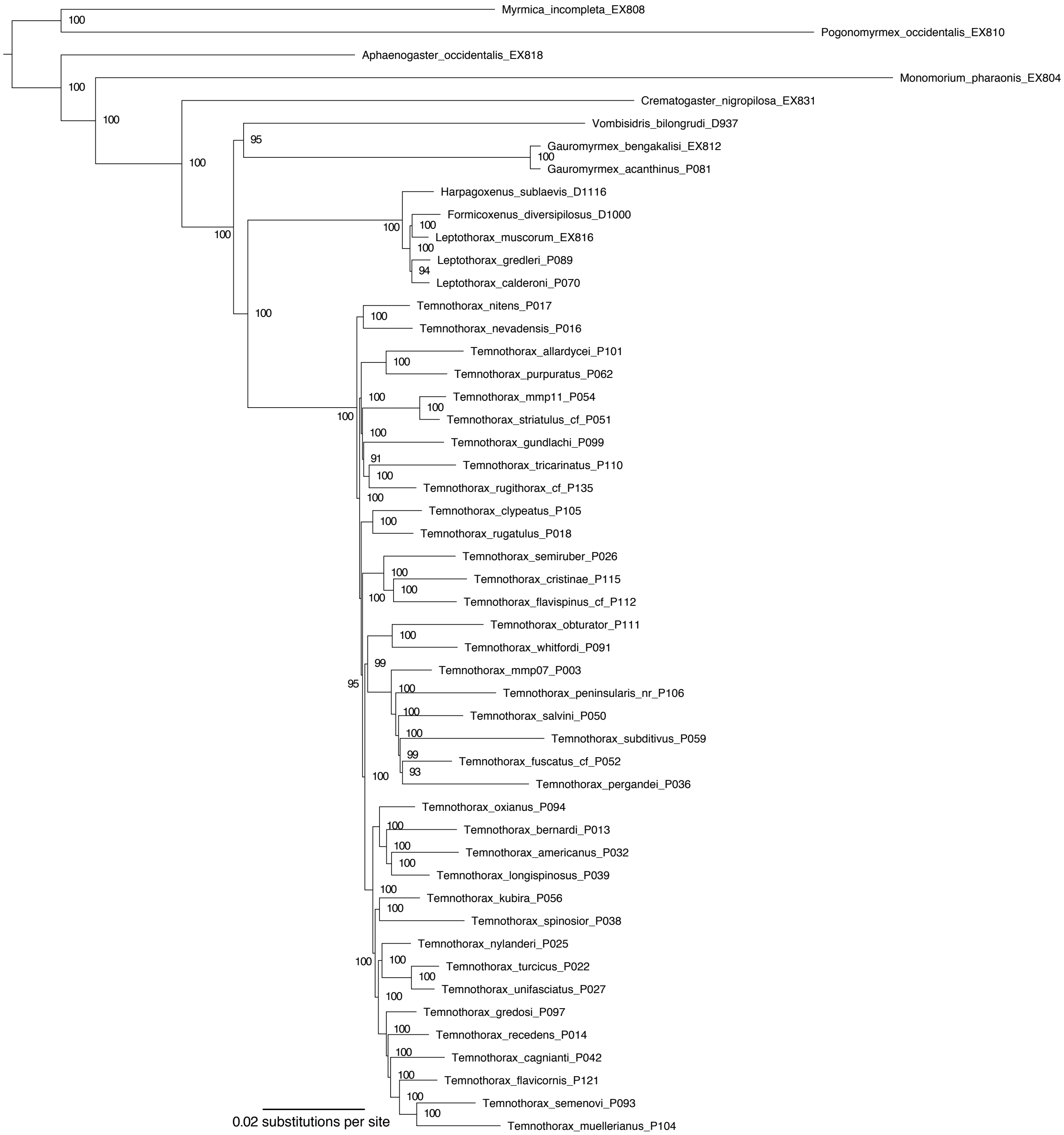

Figure M: rcfv33

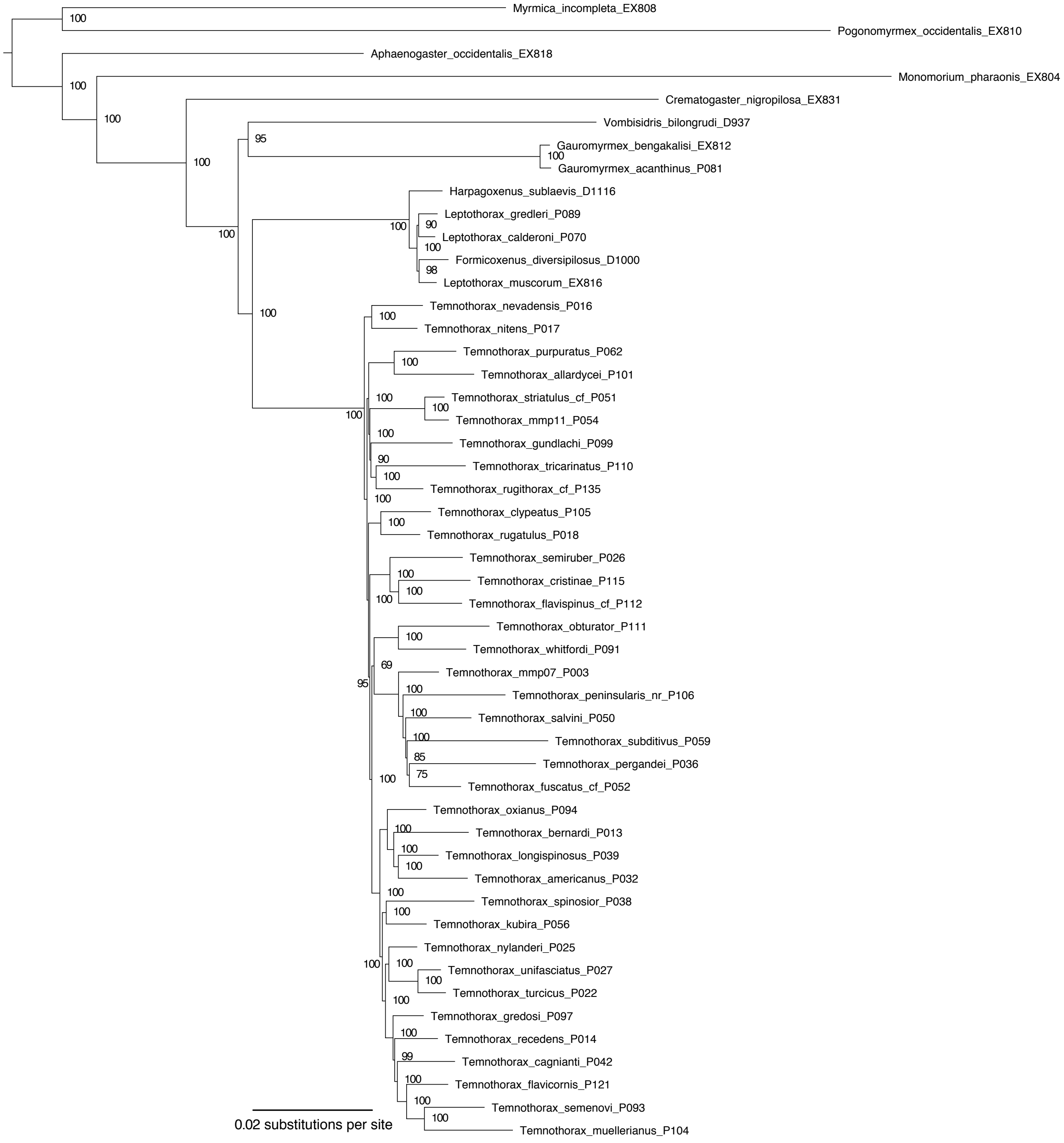

Figure N: rcfv66

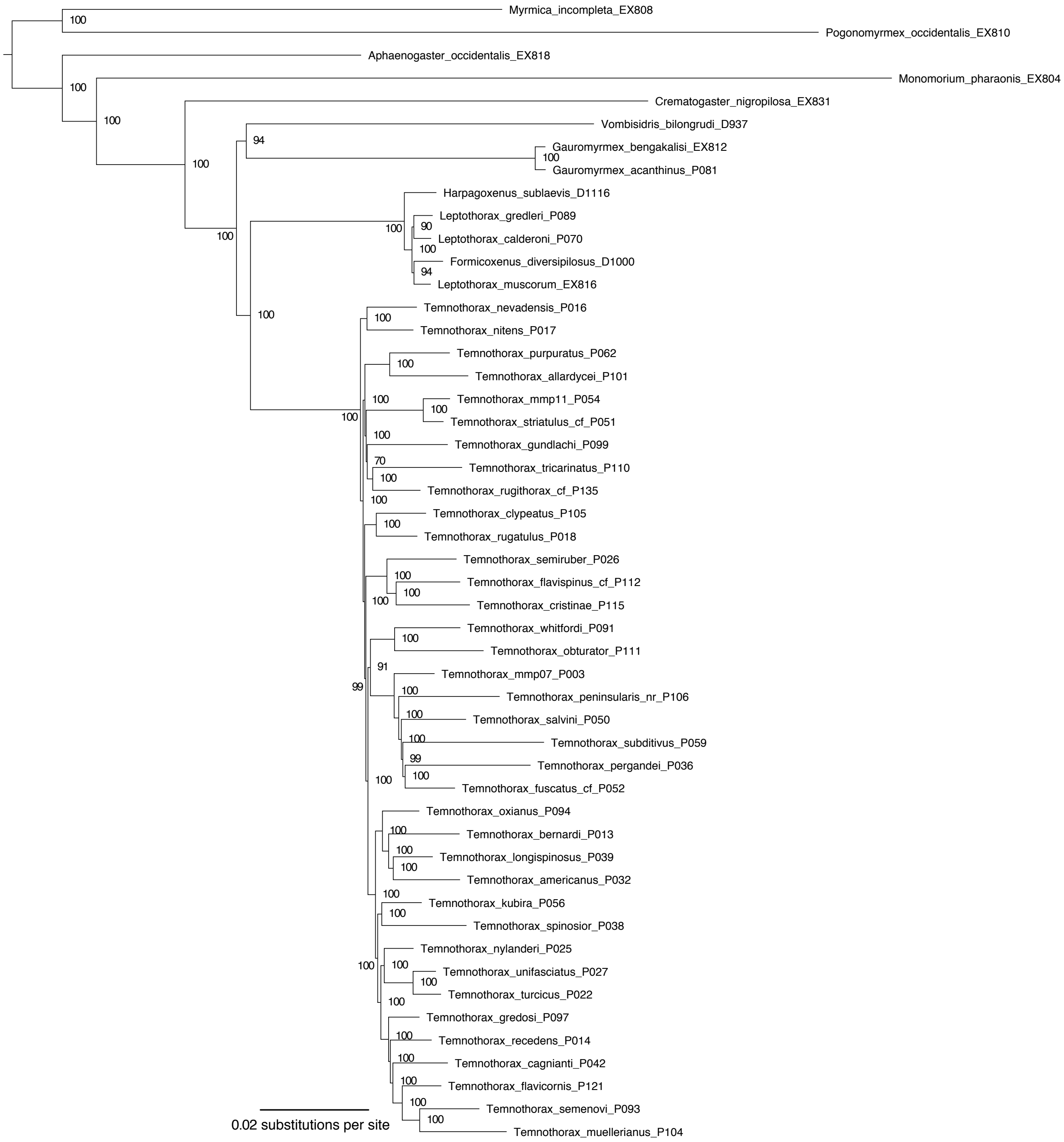

Figure O: rcfv90

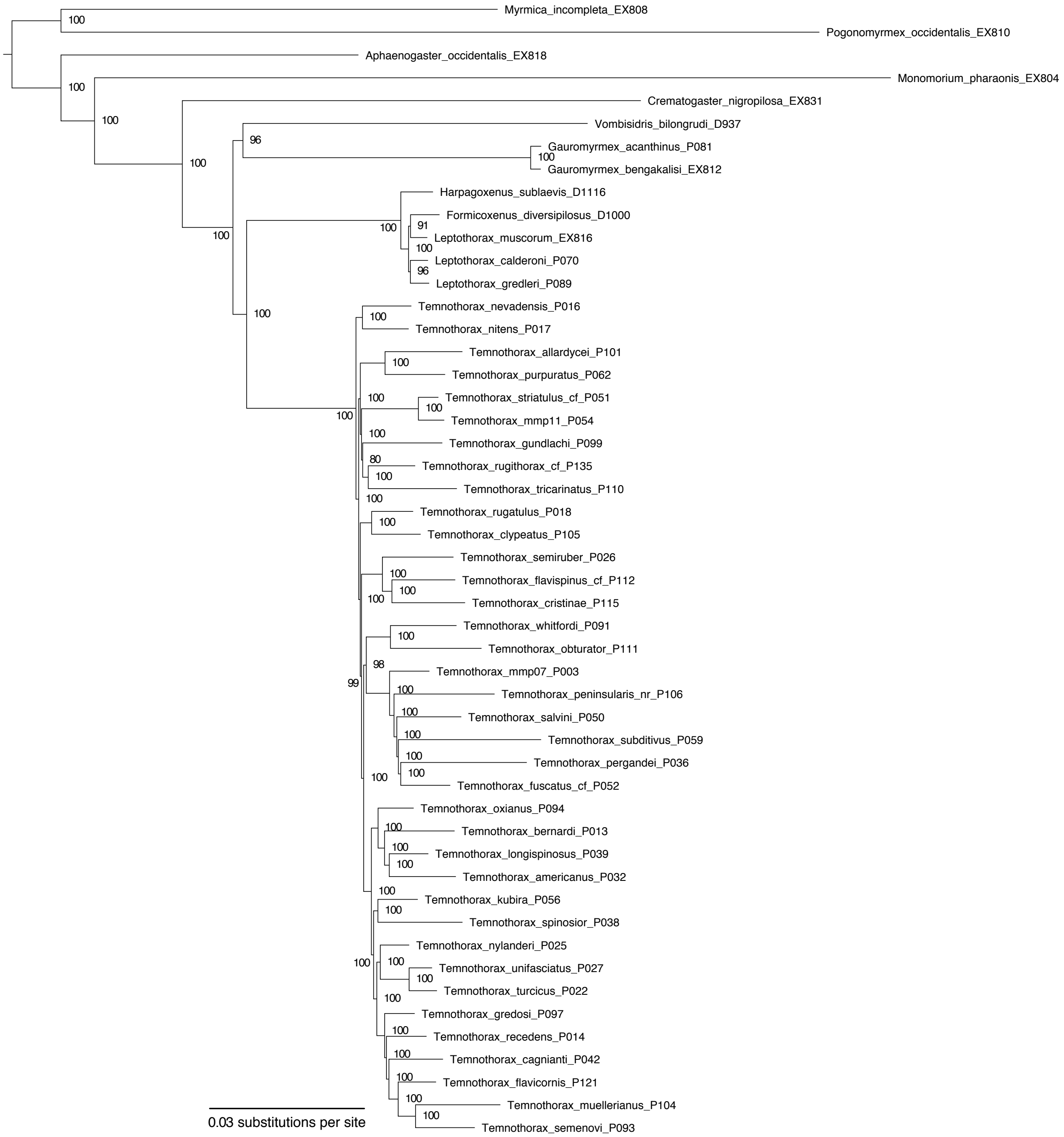

Figure P: slow33

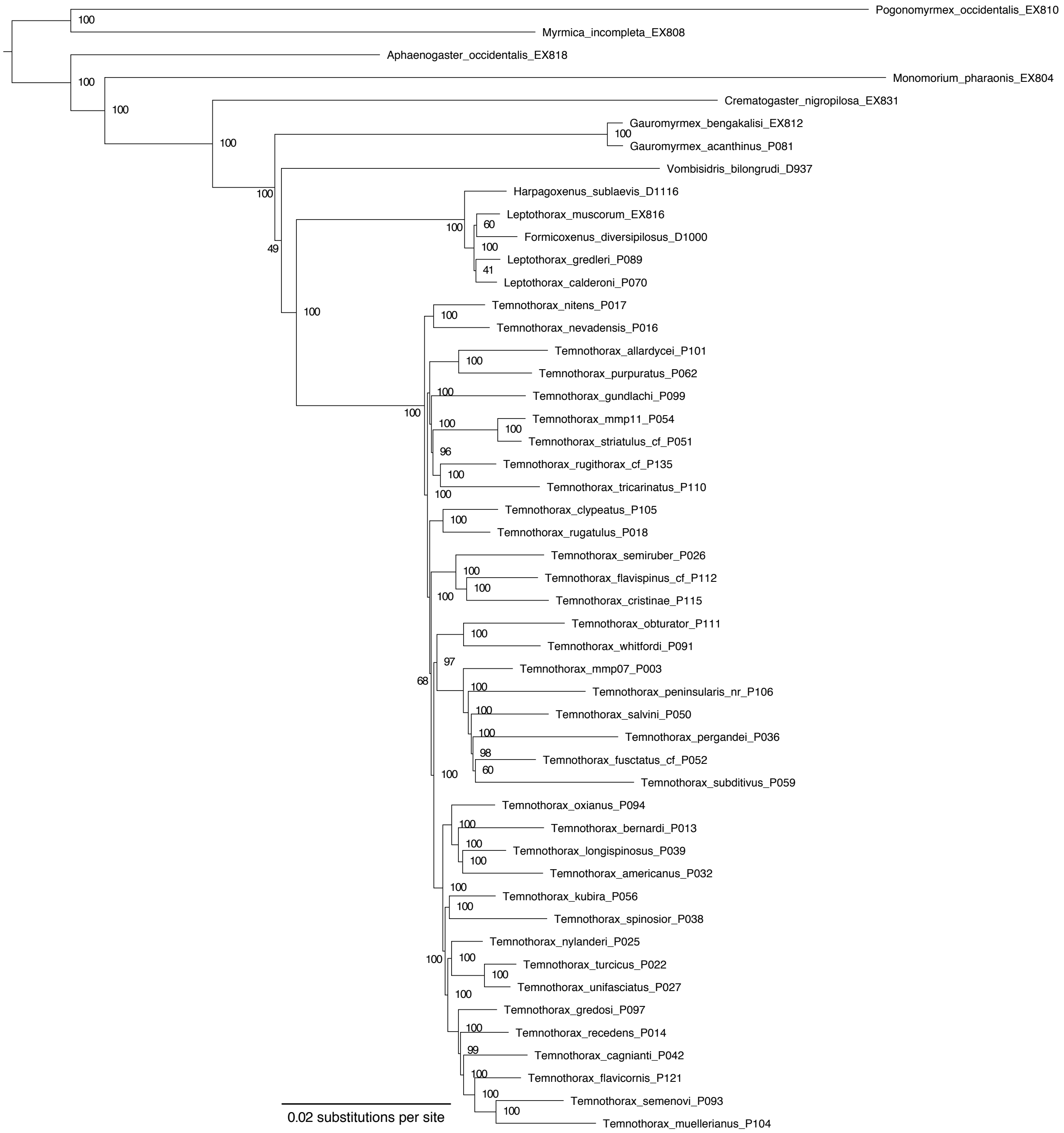

Figure Q: slow66

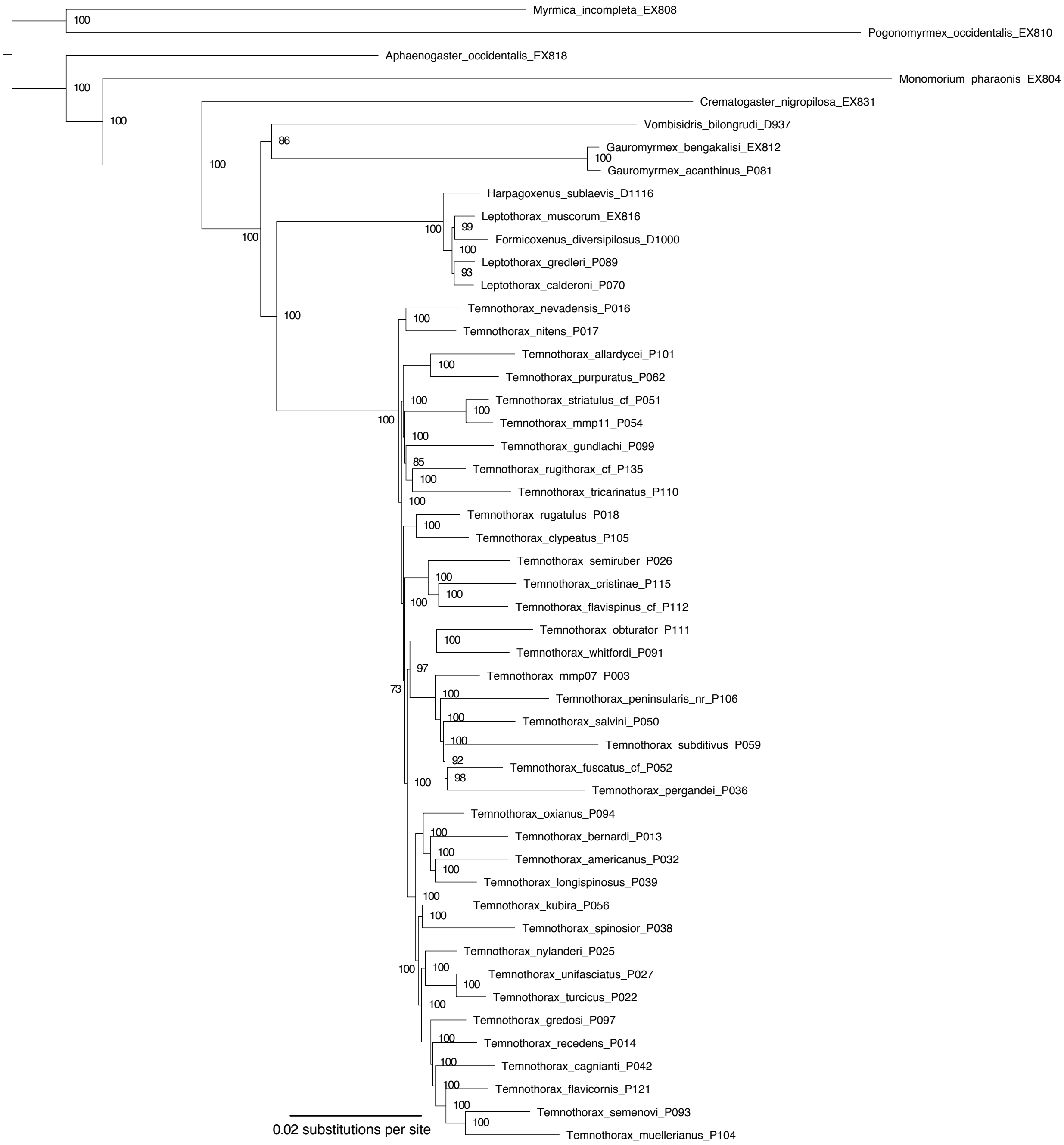

Figure R: slow90

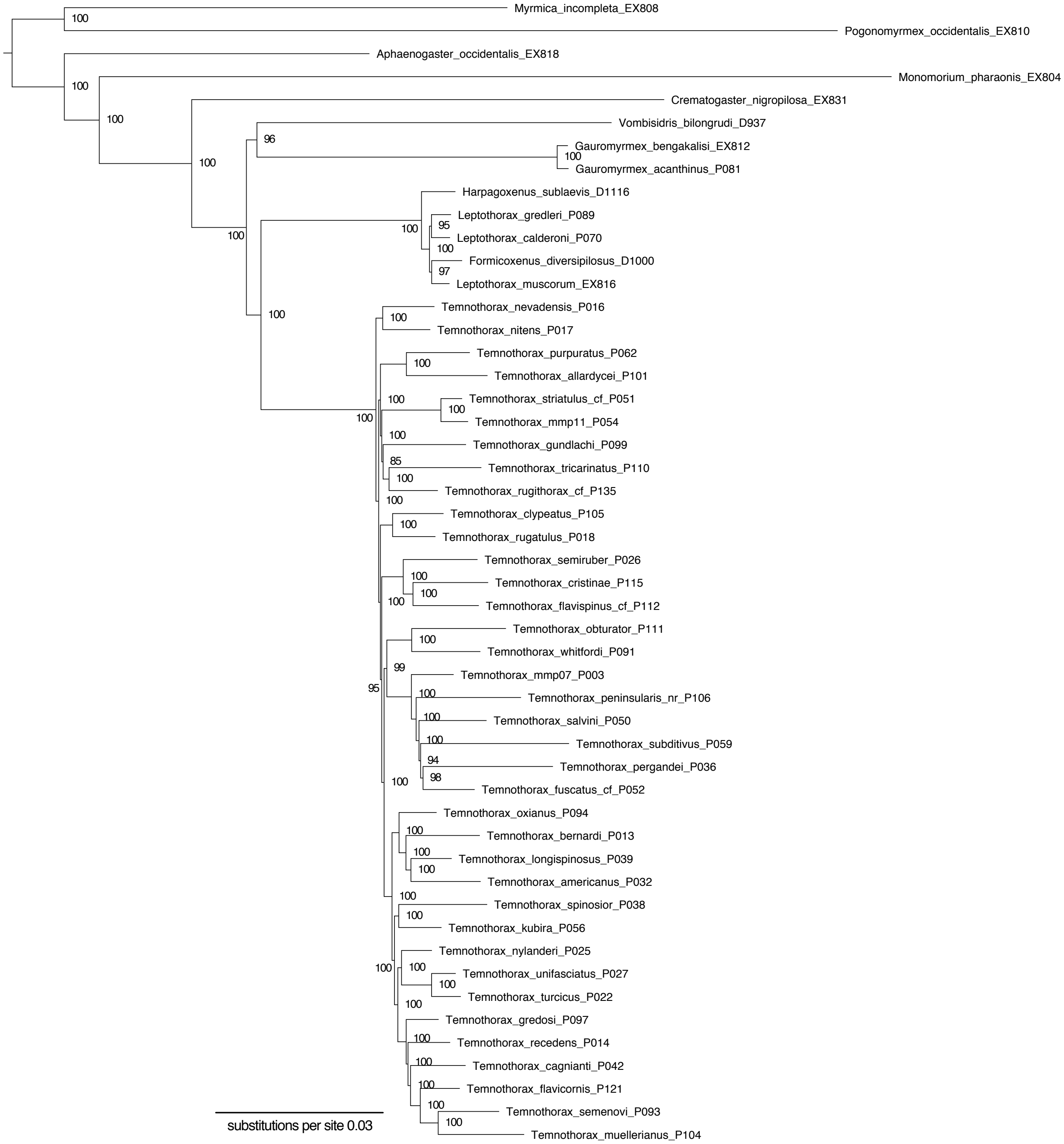

Figure S: ML kmeans

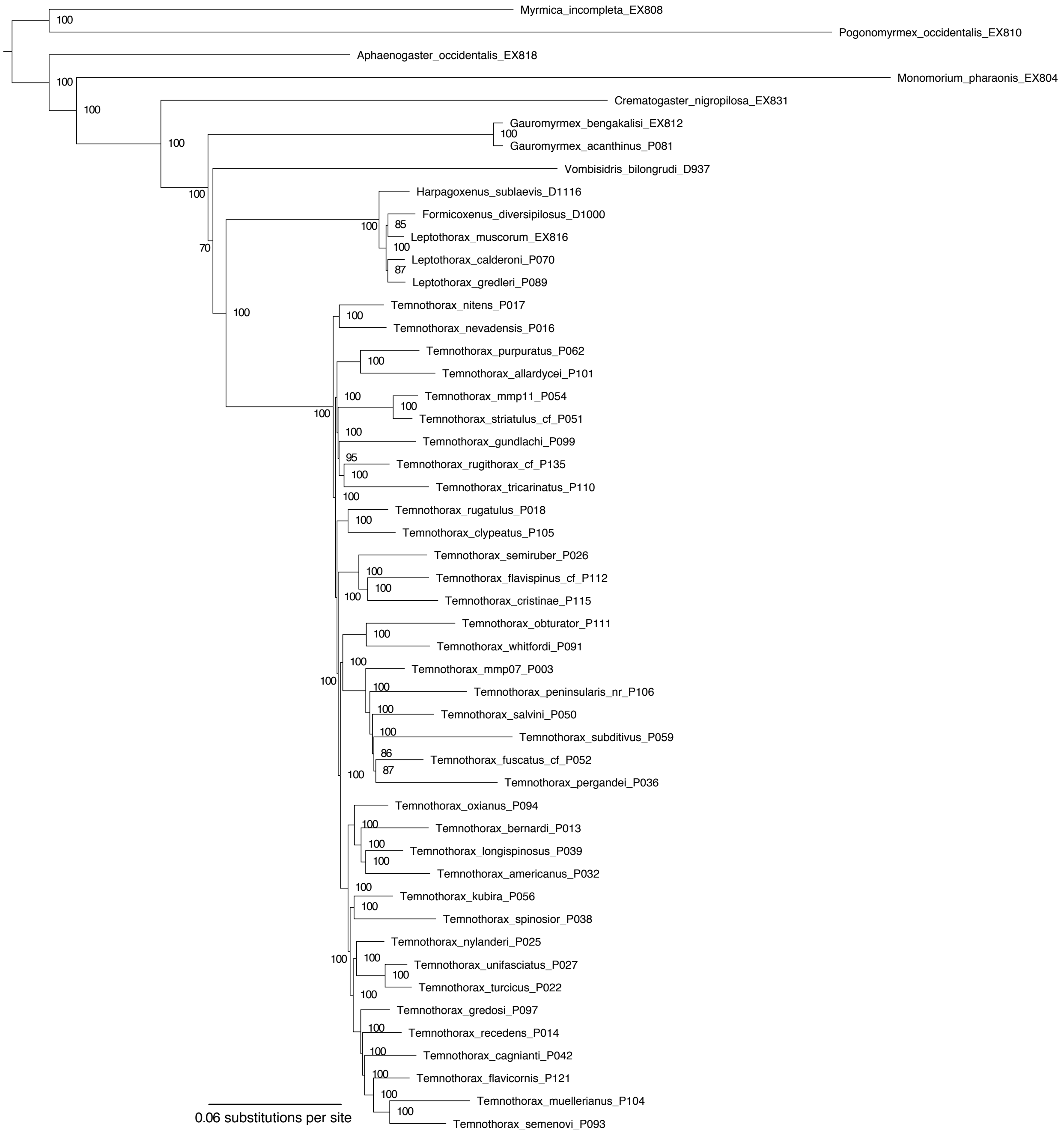

Figure T: ML rcluster

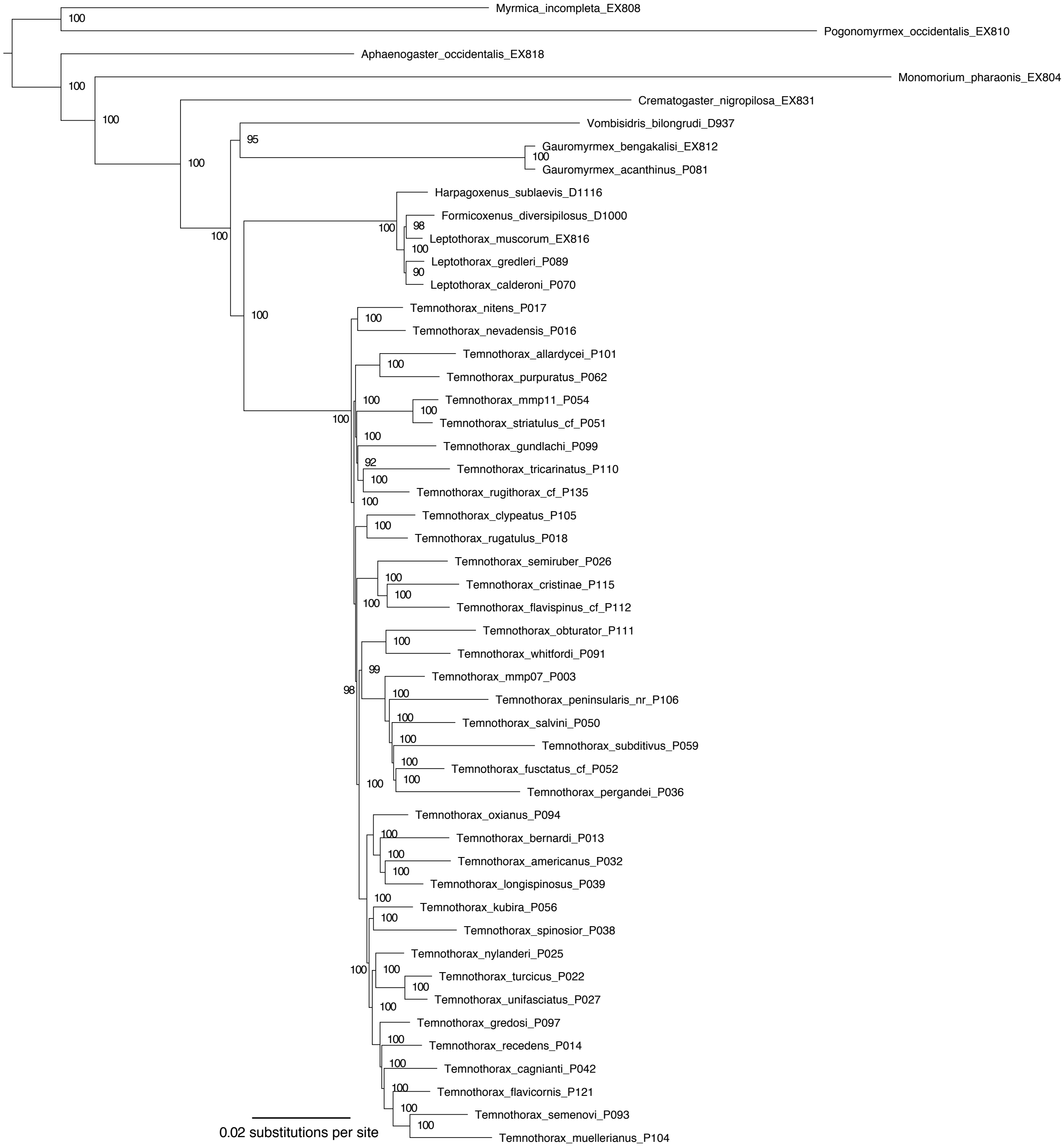

Figure U: ML rcluster coding

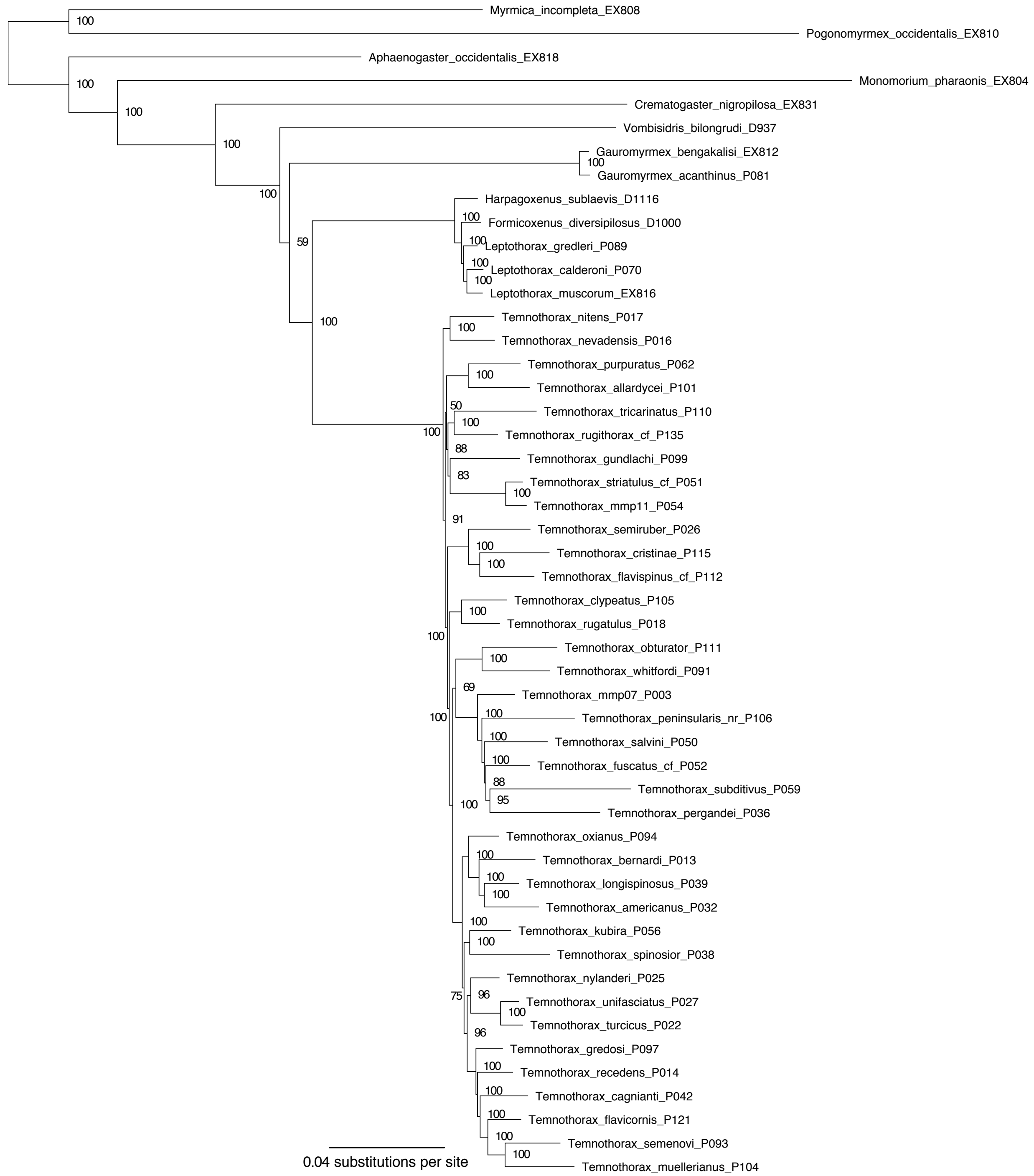

Figure V: BI kmeans

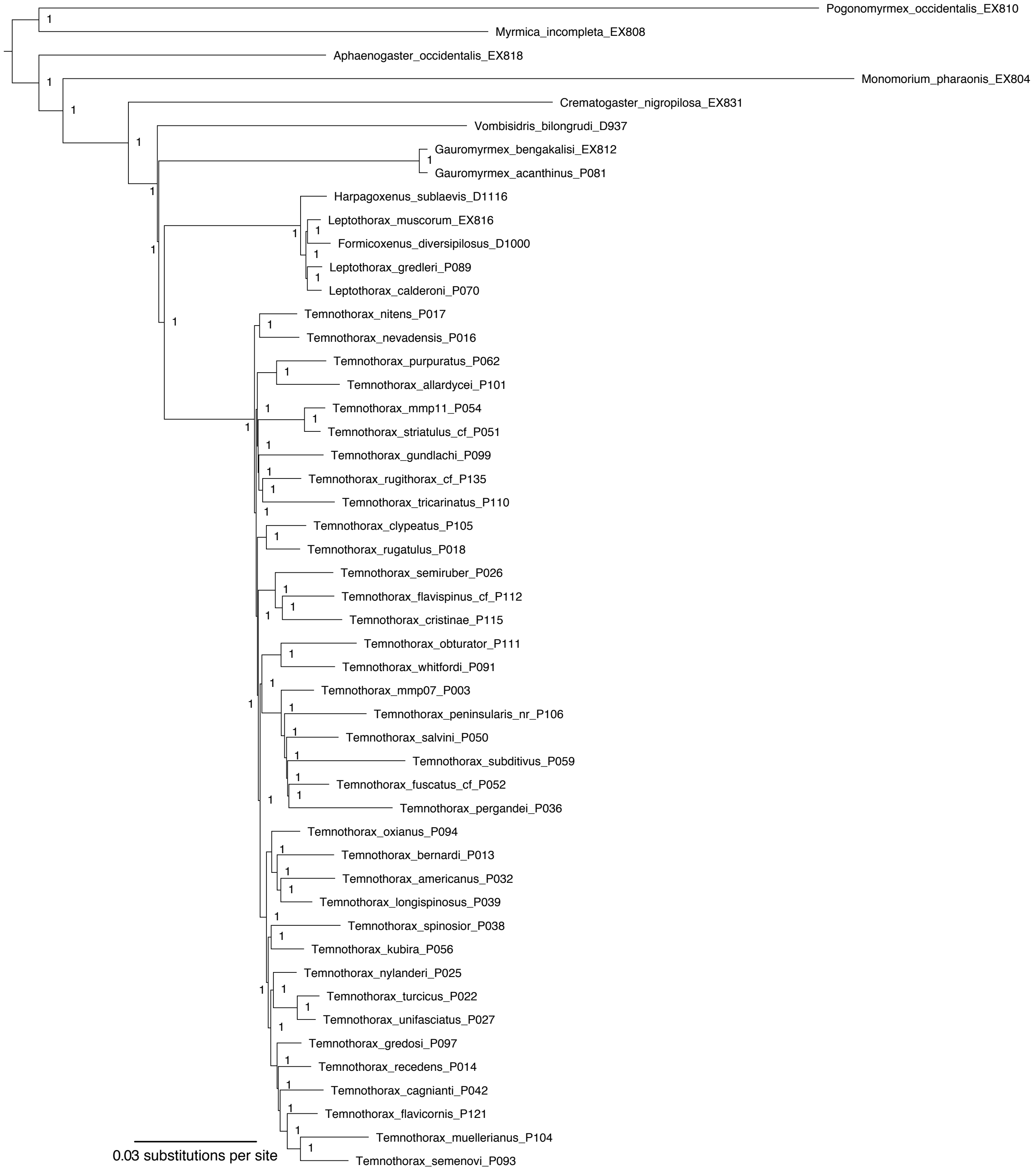

Figure W: BI rcluster

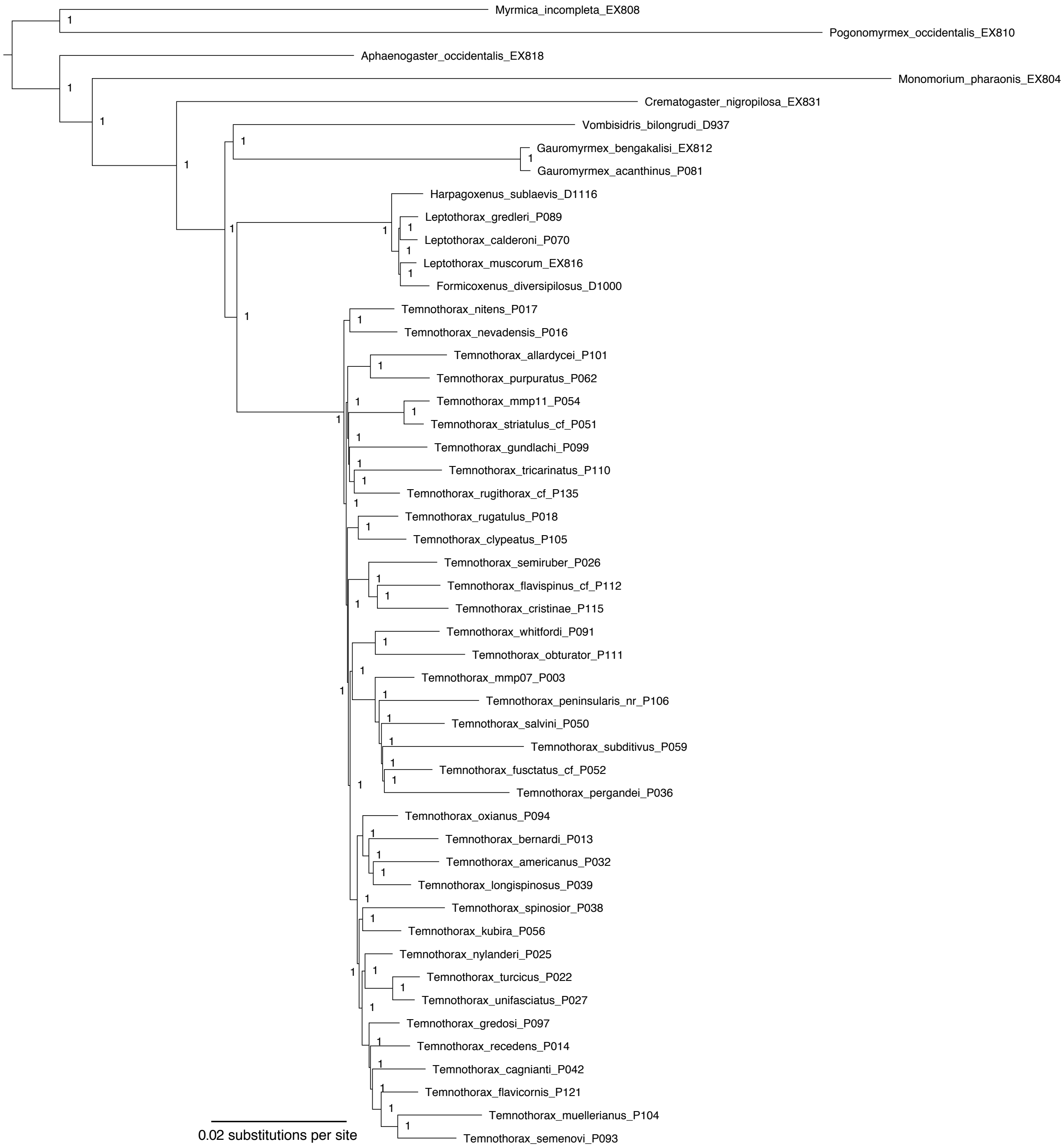

Figure X: BI rcluster coding

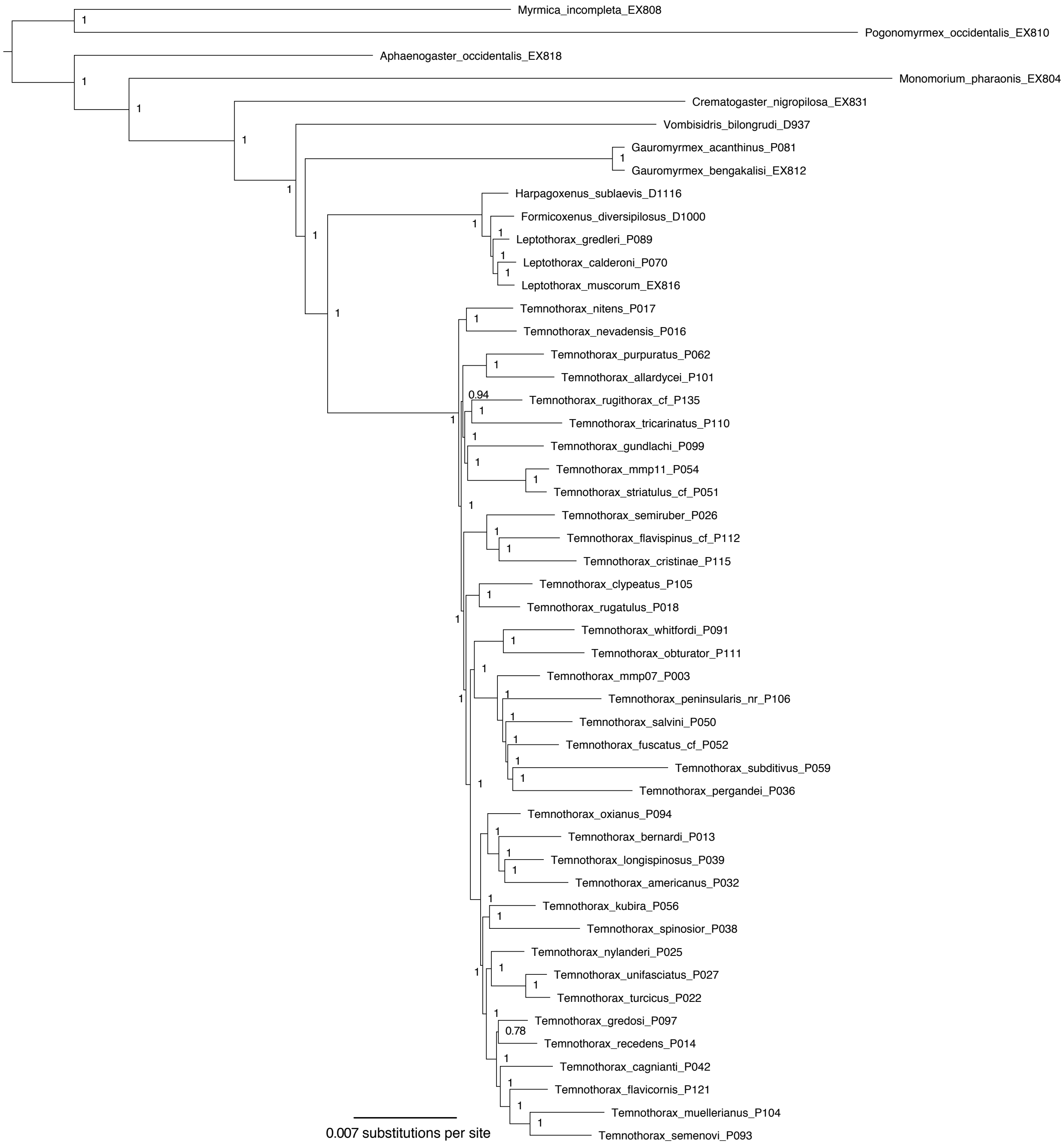

Figure Y: ASTRAL

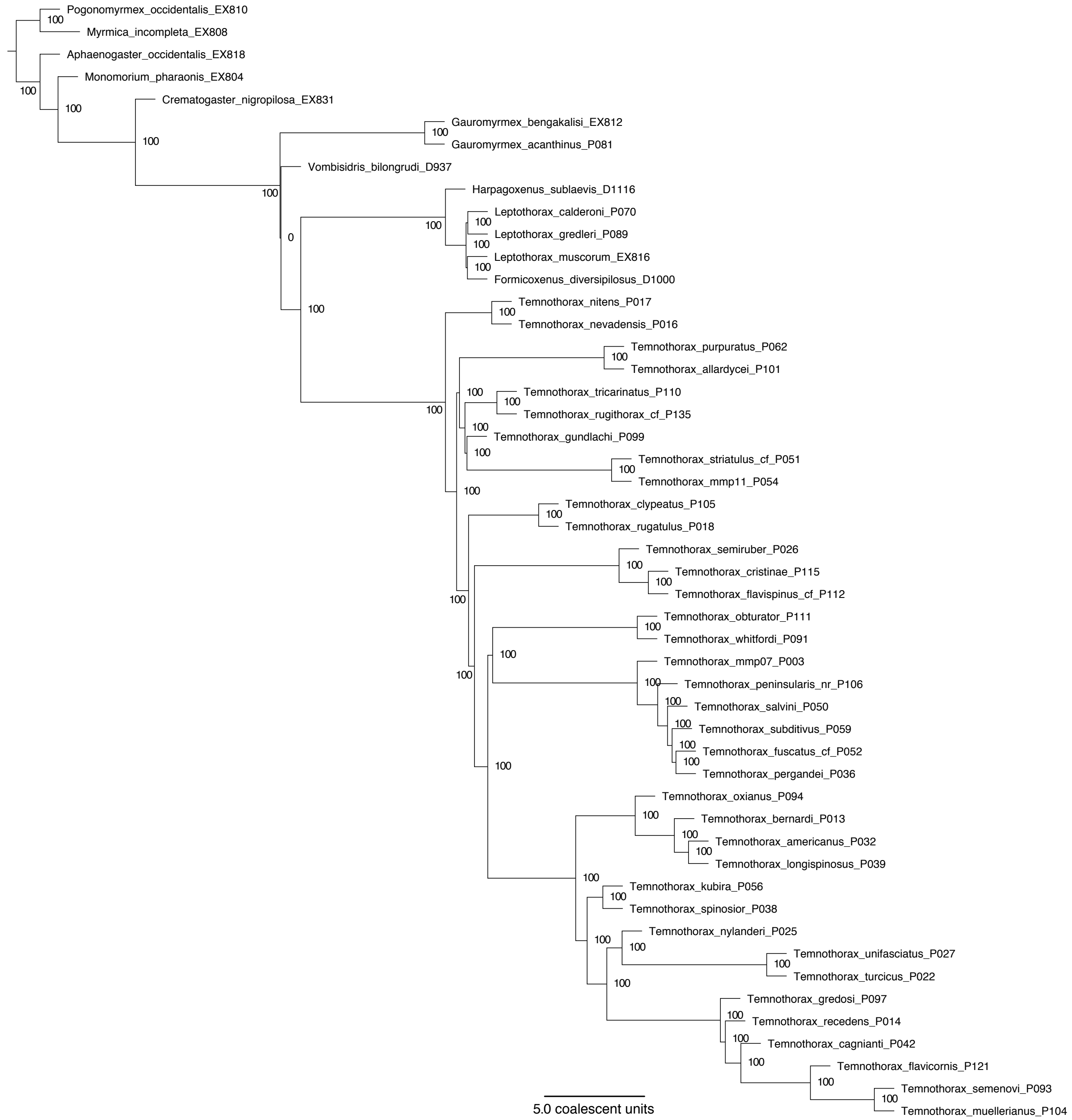

Additional file 17 Best maximum likelihood (ML, estimated with RAxML; node support values in bootstraps), Bayesian inference (BI, estimated with ExaBayes, node support values shown as posterior probability), and species trees (estimated with ASTRAL-II) based on filtered UCE datasets. **A:** min_25 ML tree; **B:** min_50 ML tree; **C:** min_75 ML tree; **D:** min_90 ML tree; **E:** min_95 ML tree; **F:** min_99 ML tree; **G:** min_100 ML tree; **H:** rand_33_1 ML tree; **I:** rand_33_2 ML tree; **J:** rand_33_3 ML tree; **K:** rand_66 ML tree; **L:** rand_99 ML tree; **M:** rcfv_33 ML tree; **N:** rcfv_66 ML tree; **O:** rcfv_90 ML tree; **P:** slow_33 ML tree; **Q:** slow_66 ML tree; **R:** slow_90 ML tree; **S:** kmeans ML tree; **T:** rcluster ML tree; **U:** rcluster coding ML tree; **V:** kmeans BI tree; **W:** rcluster BI tree; **X:** rcluster coding BI tree; **Y:** ASTRAL-II species tree.
