# Supplementary material for: Insights into the evolution, biogeography and natural history of the acorn ants, genus Temnothorax Mayr (hymenoptera: Formicidae)
Source: BMC Evol Biol. 2017 Dec 13;17:250. doi: 10.1186/s12862-017-1095-8 (PMC5729518; doi:10.1186/s12862-017-1095-8)
Supplement: Supplementary file 19 — BI trees based on morphology, and morphology + molecular data. (PDF 75 kb) [file 12862_2017_1095_MOESM19_ESM.pdf]

Figure A: Empirical data

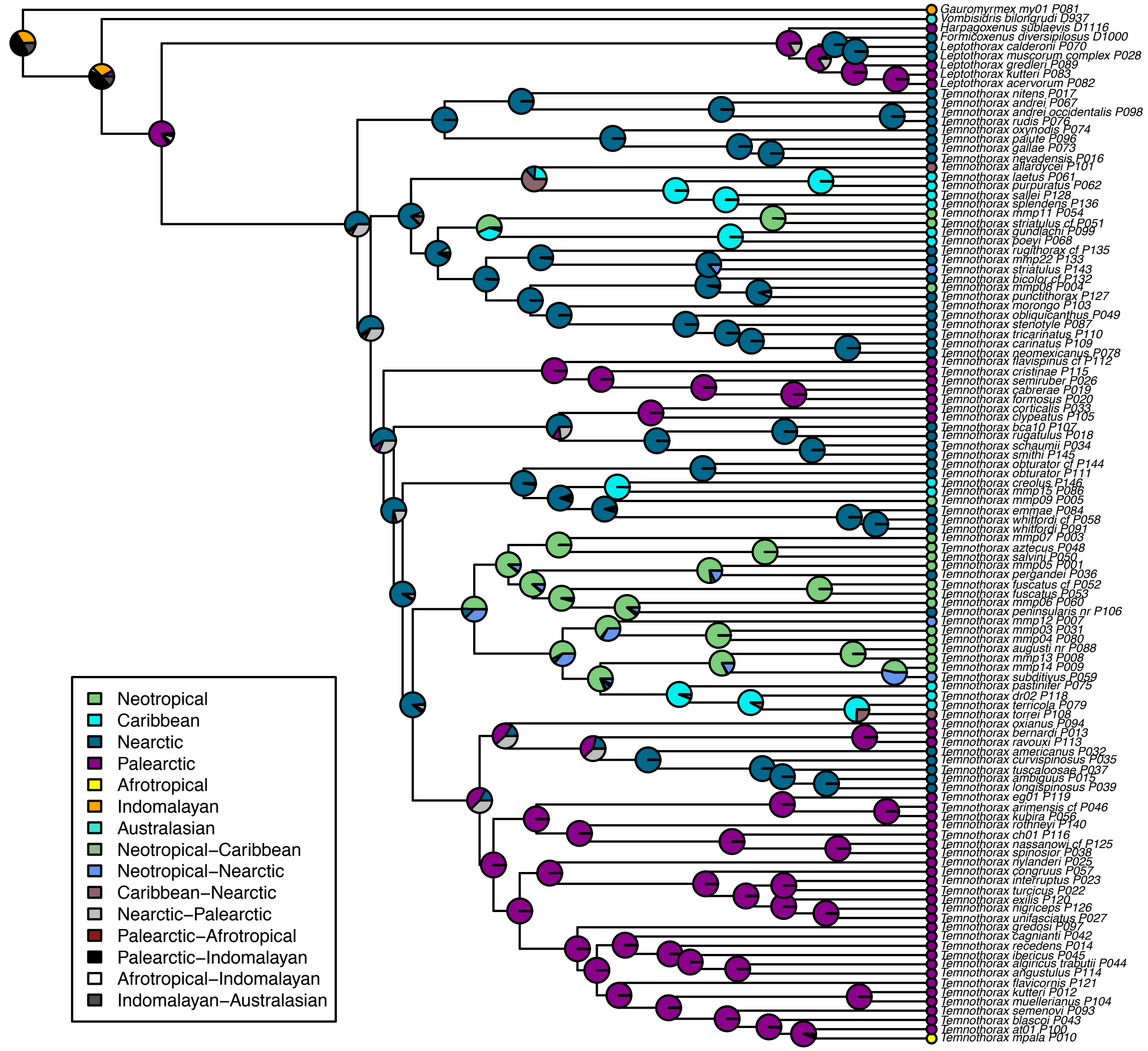

Figure A. Model-averaged biogeographic reconstruction of *Temnothorax* inferred using BioGeoBEARS with empirical data.

Figure B: Outgroup sensitivity

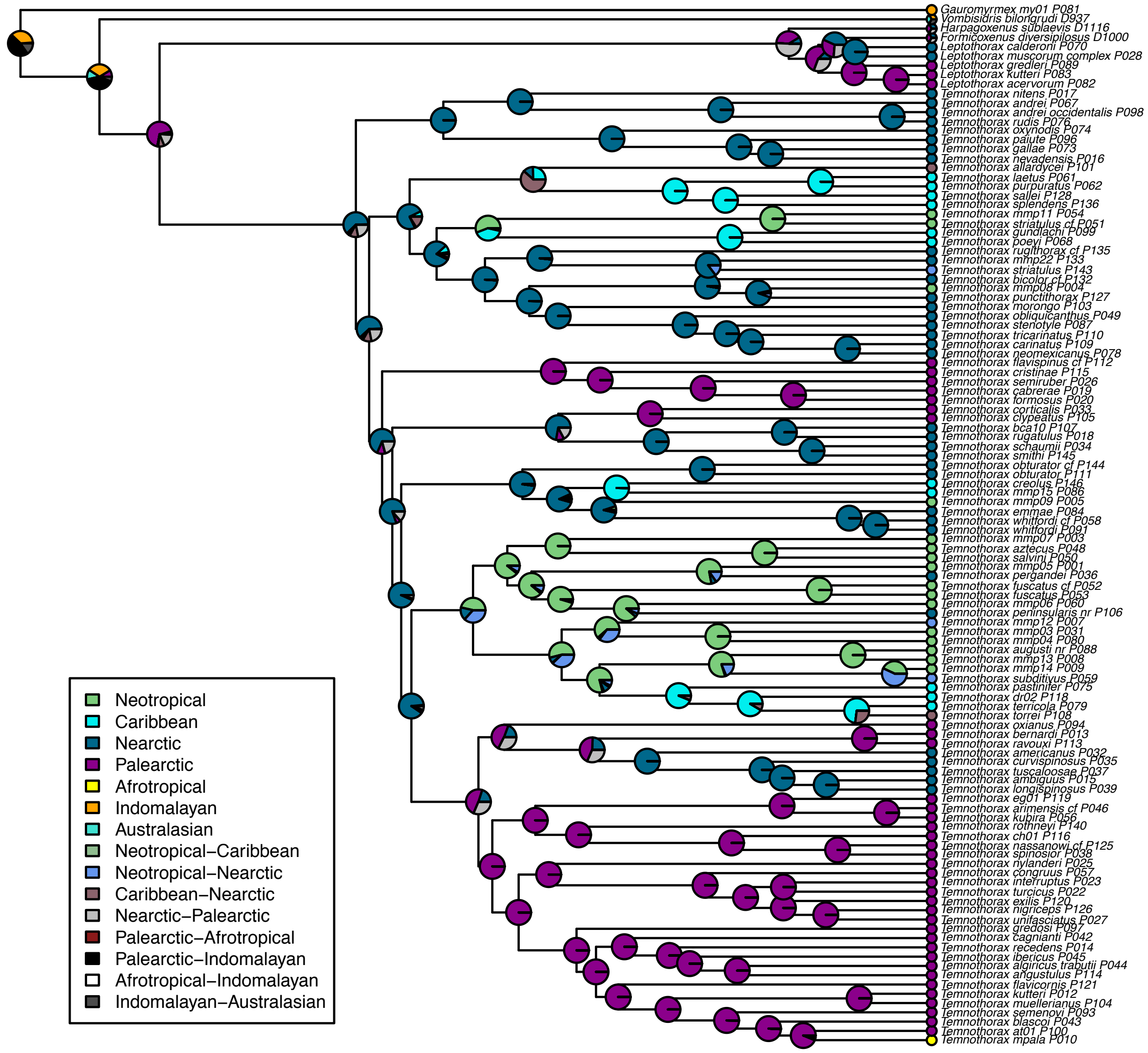

Figure B. Model-averaged biogeographic reconstruction of *Temnothorax* inferred using BioGeoBEARS with tip data permutations to test the sensitivity of the ingroup reconstruction to incomplete outgroup sampling.
