# Supplementary material for: Insights into the evolution, biogeography and natural history of the acorn ants, genus Temnothorax Mayr (hymenoptera: Formicidae)
Source: BMC Evol Biol. 2017 Dec 13;17:250. doi: 10.1186/s12862-017-1095-8 (PMC5729518; doi:10.1186/s12862-017-1095-8)
Supplement: Supplementary file 22 — Biogeographic reconstructions inferred from BioGeoBEARS anaylsis of empirical data and sensitivity analyses. (PDF 376 kb) [file 12862_2017_1095_MOESM22_ESM.pdf]

Figure A: morphology

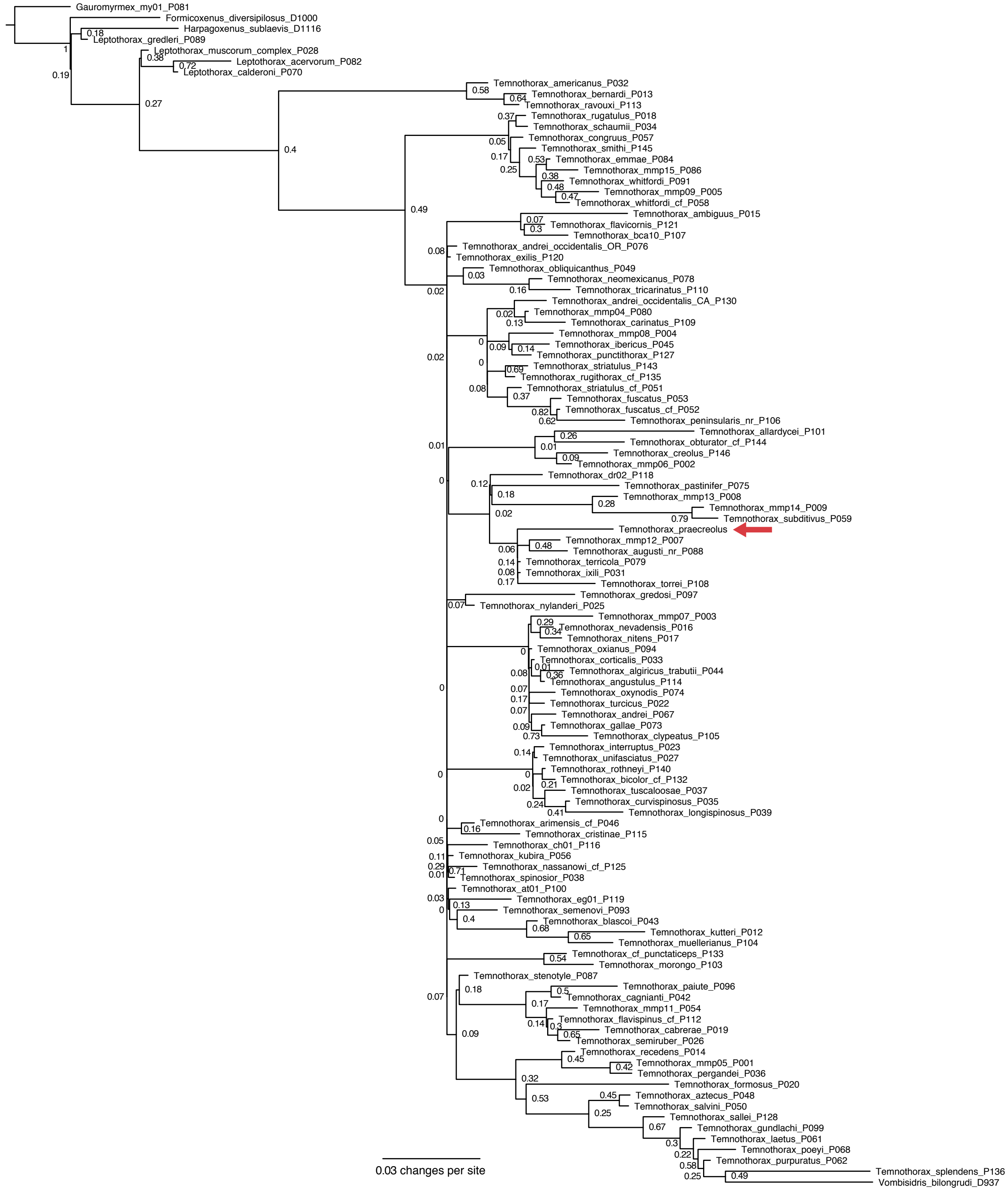

Figure B: morphology & molecular data

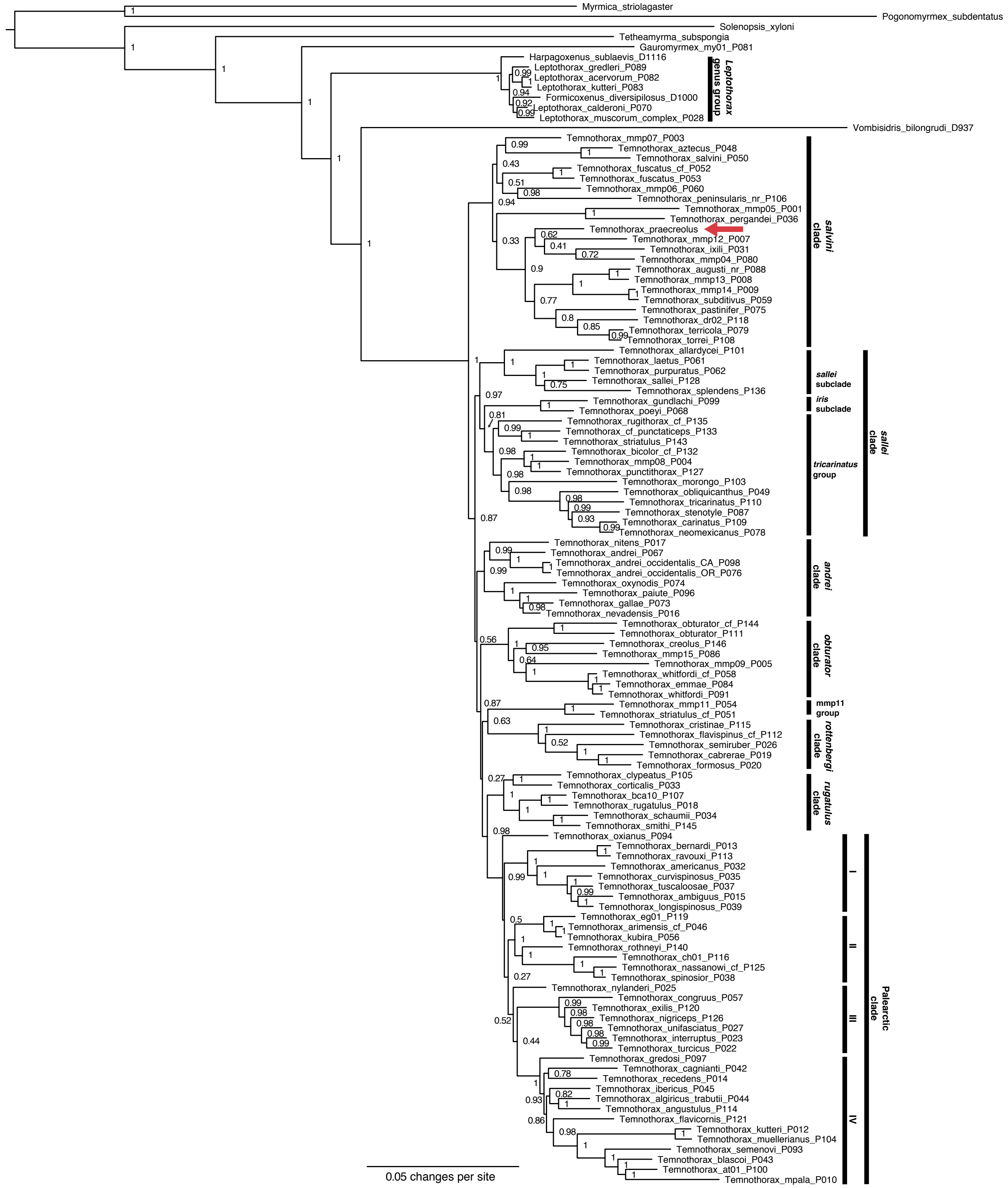

Additional file 22 Bayesian inference trees estimated with MrBayes. Red arrow indicates position of the Dominican Amber fossil species *Temnothorax praecreolus*. Node support values shown as posterior probabilities. **A:** morphology only dataset. **B:** morphology and molecular data combined.
