# Supplementary material for: Insights into the evolution, biogeography and natural history of the acorn ants, genus Temnothorax Mayr (hymenoptera: Formicidae)
Source: BMC Evol Biol. 2017 Dec 13;17:250. doi: 10.1186/s12862-017-1095-8 (PMC5729518; doi:10.1186/s12862-017-1095-8)
Supplement: Supplementary file 23 — Character dependent diversification rates and ancestral state reconstructions inferred with HiSSE models. (PDF 1361 kb) [file 12862_2017_1095_MOESM23_ESM.pdf]

Figure A: Arboreality full dataset rates

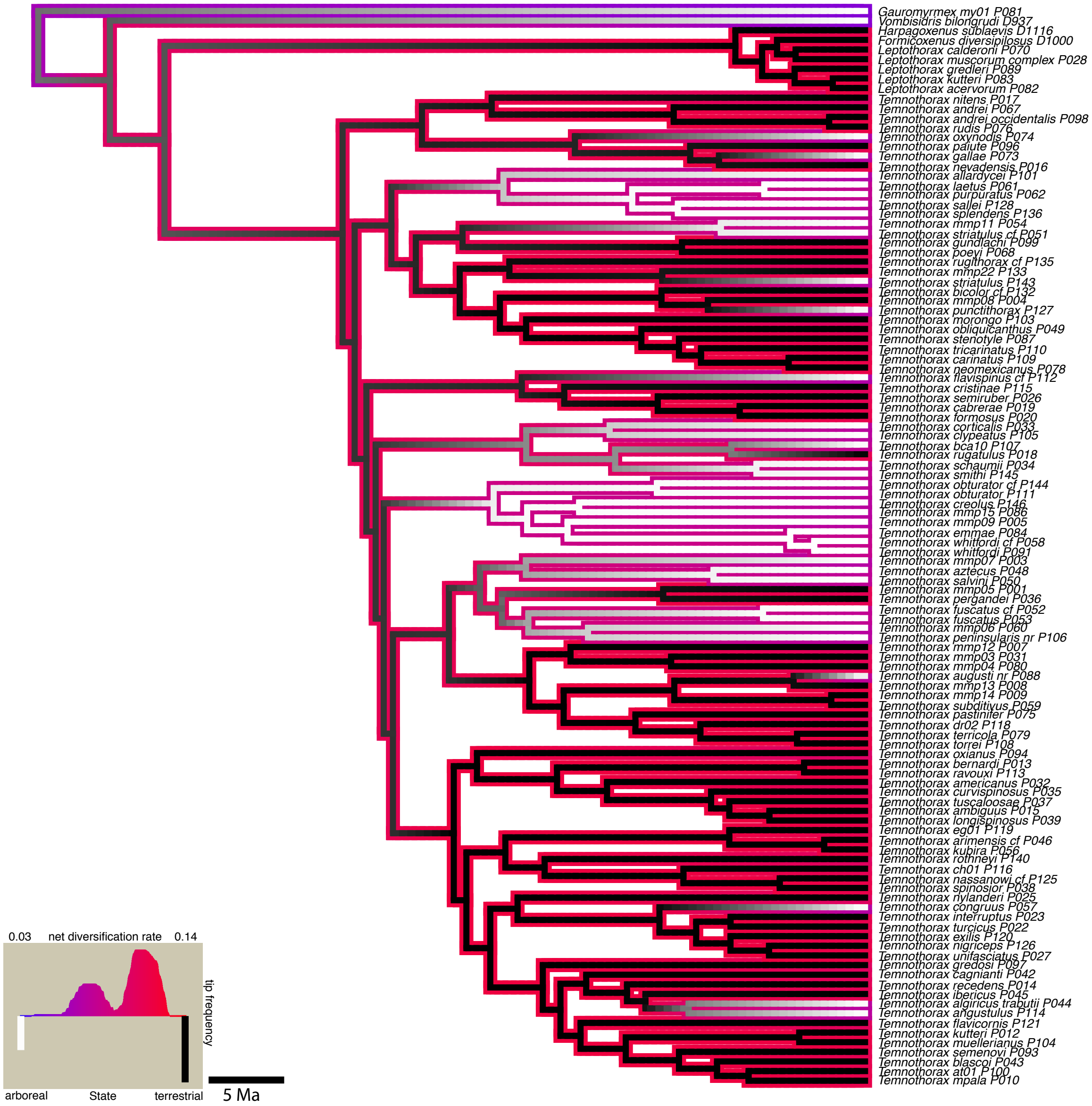

Figure B: Arboreality full dataset ASR

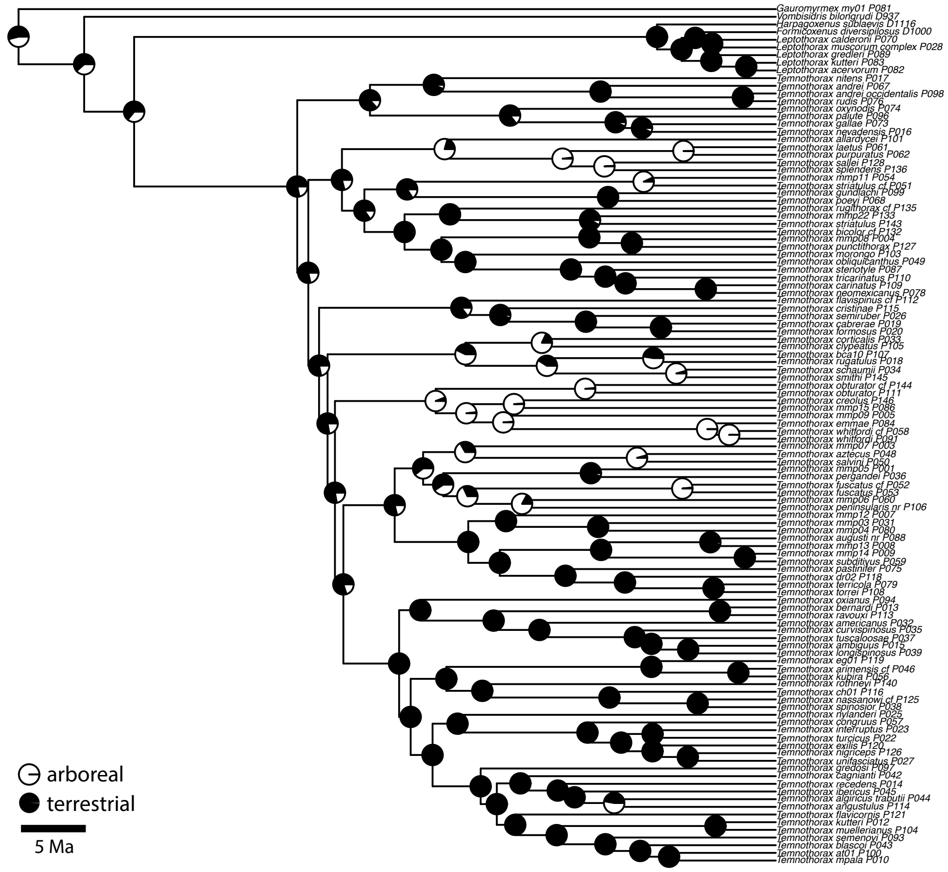

Figure C: Arboreality reduced dataset rates

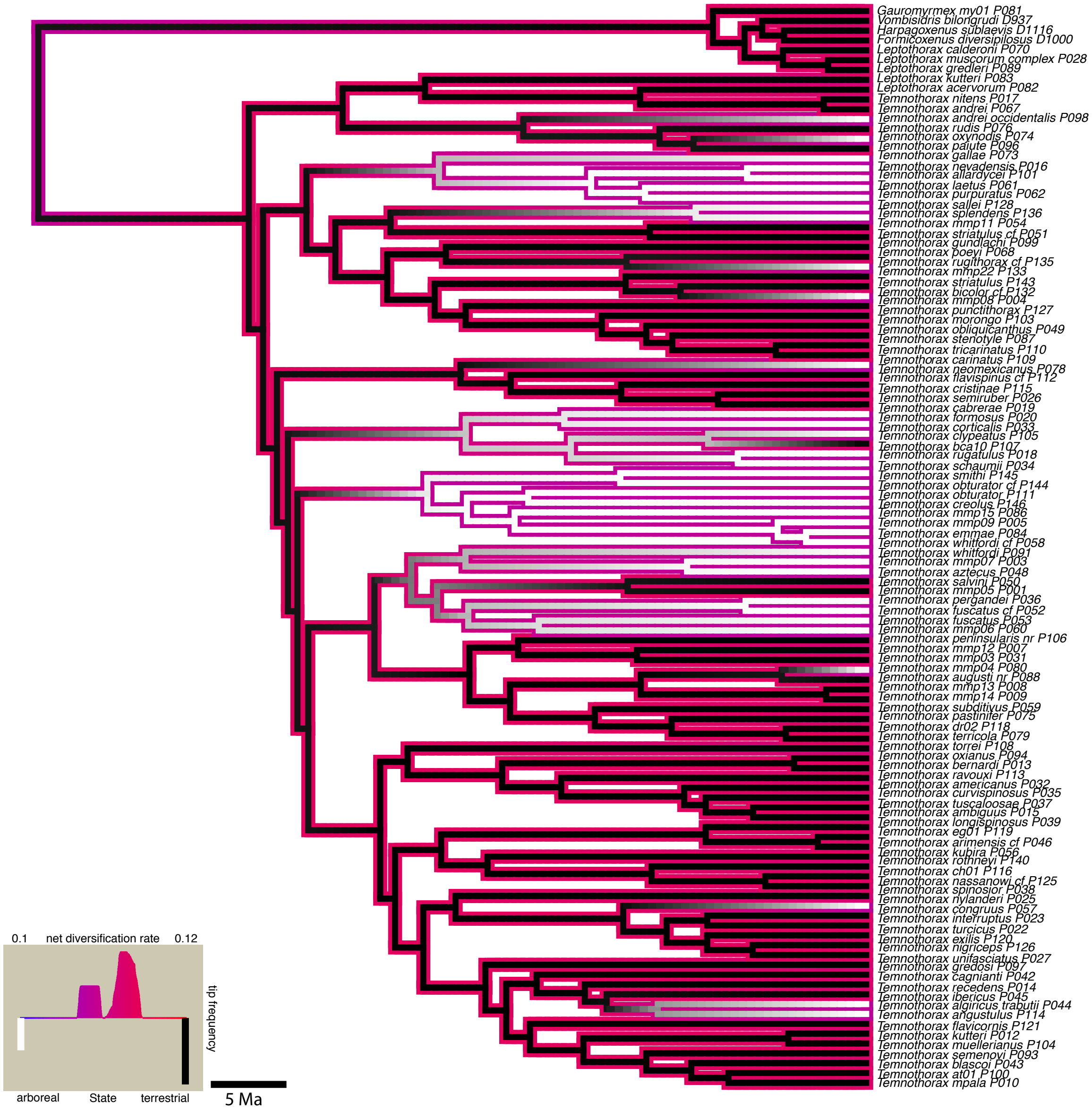

Figure D: Arboreality reduced dataset ASR

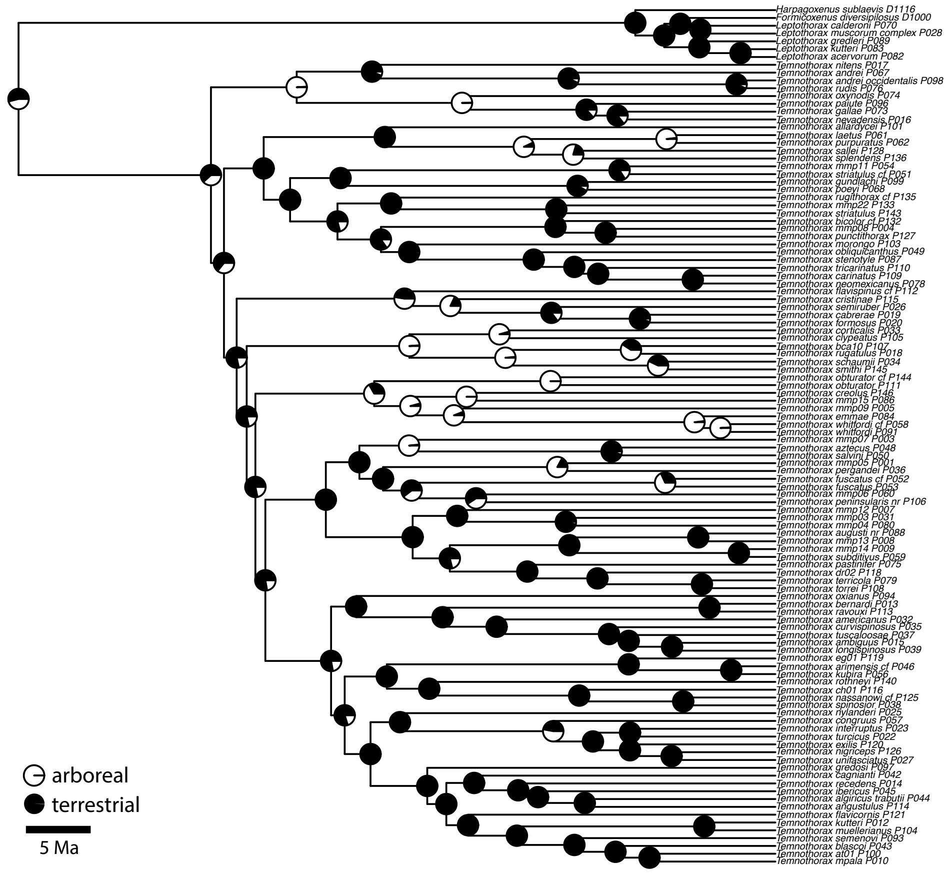

Figure E: Parasitism full dataset rates

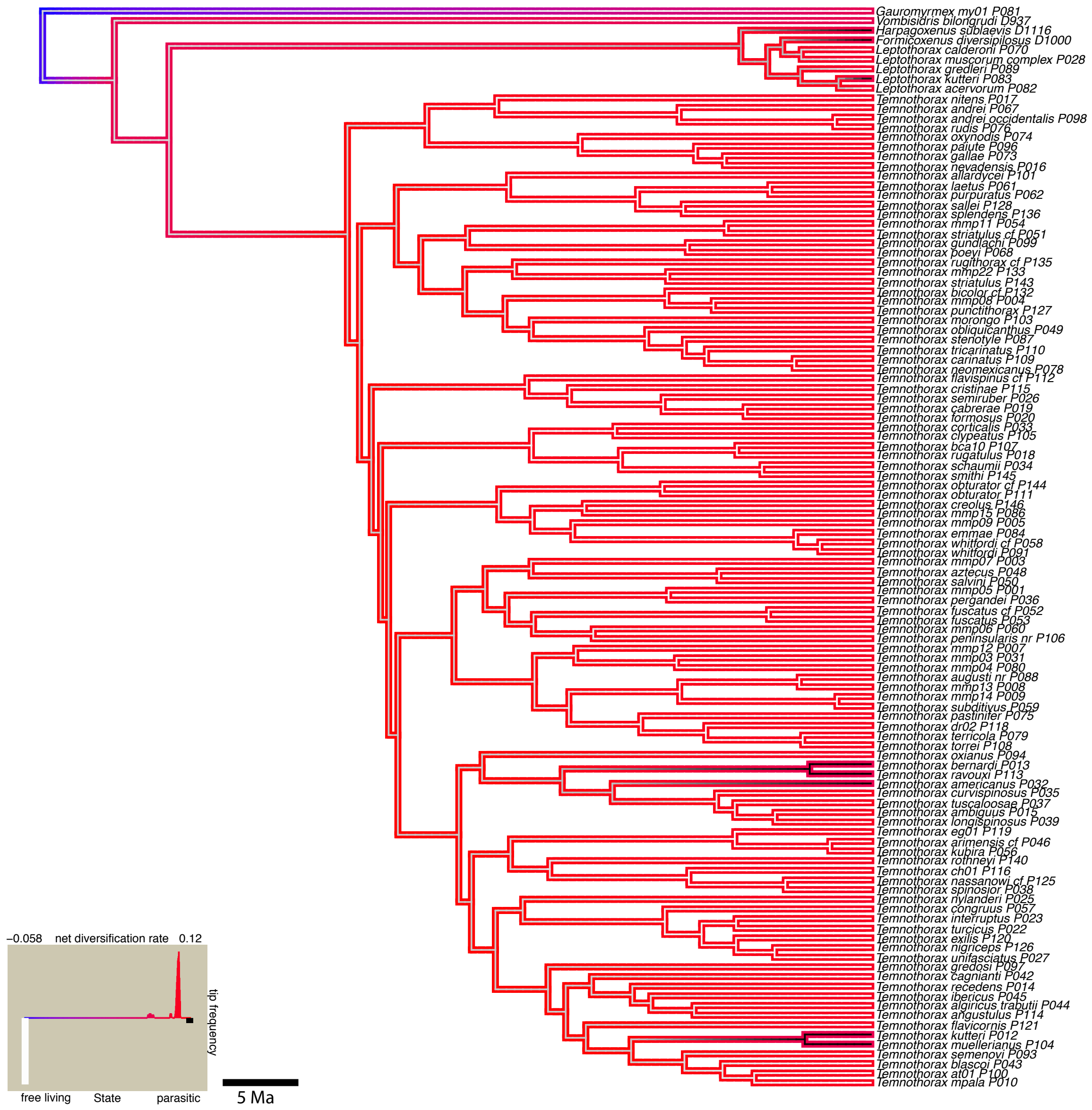

Figure F: Parasitism full dataset ASR

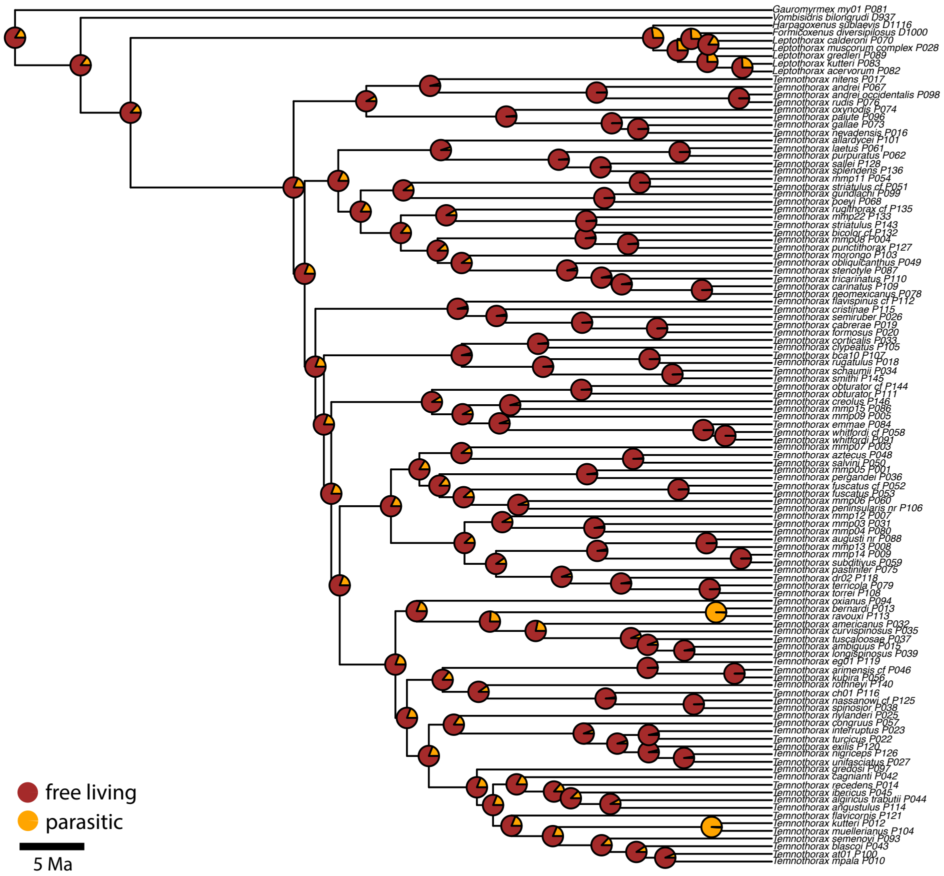

Additional file 23 Character dependent diversification rates and ancestral state reconstructions inferred with HiSSE models. All figures are of model-averaged reconstructions. **A.** Diversification rate reconstruction of the full arboreality dataset, including long branched outgroups. **B.** Ancestral state reconstruction of the full arboreality dataset, including long branched outgroups. **C.** Diversification rate reconstruction of the reduced arboreality dataset. **D.** Ancestral state reconstruction of the reduced arboreality dataset. **E.** Diversification rate reconstruction of the parasitism dataset. **F.** Ancestral state reconstruction of the parasitism dataset.
